# Corporate Social Responsibility: A Real Options Approach to the Challenge of Financial Sustainability

**DOI:** 10.1371/journal.pone.0125972

**Published:** 2015-05-04

**Authors:** Maria-Teresa Bosch-Badia, Joan Montllor-Serrats, Maria-Antonia Tarrazon-Rodon

**Affiliations:** 1 Department of Economics, Universitat de Girona, Campus de Montiliví, 17071, Girona, Spain; 2 Business Department, Universitat Autònoma de Barcelona, Campus de Bellaterra, 08193, Bellaterra (Cerdanyola del Vallès), Spain; Kingston University London, UNITED KINGDOM

## Abstract

**Background:**

In contemporary complex societies, social values like ethics, corporate social responsibility, and being respectful with the environment, among others, are becoming social requirements. Corporations are expected to fulfill them and, according to empirical evidence, an overwhelming majority aspires to good social valuation. At the same time, the maximization of market share value in the long run continues to be the central corporate goal. Making environmental and social expenses compatible with value creation is a central challenge for corporations since it implies the financial sustainability of Corporate Social Responsibility (CSR).

**Methods and Results:**

The value creation capacity of CSR projects, mainly through innovation, is widely acknowledged in economic literature and corporate practice. This fact arouses the need of having a quantitative framework capable of summarizing the value creation capacity of the variables involved in CSR projects. With this aim we build up a sensitivity analysis of real option ratios that studies and quantifies the value creation capacity of CSR projects connected with innovation. Ratio analysis has the advantage of being scale independent. Hence, it furnishes a homogeneous framework to express the interaction of value creation variables and, thus, supports strategic thinking quantitatively. Often, CSR expenses can be regarded as preliminary projects that create the opportunity to undertake a full future project. For them, we obtain the minimum expectations scenario that makes financially sustainable a preliminary project that can be interpreted as a call option. We propose a classification of CSR projects from the decision analysis perspective following a two-fold approach: Their relationship with value creation and their links with existing corporate activities. This classification of CSR projects aims at contributing to choose the best capital budgeting method to study the financial sustainability of the project and identifying those CSR projects that fulfill the required features to be studied from the real options perspective.

## Introduction

A reliable strategy for the corporate core business cannot be conceived without a reliable Corporate Social Responsibility (CSR) strategy. Stakeholders demand an efficient interaction of the three sustainability pillars: environmental, financial and social. In the long run, financial value creation is not possible without fulfilling environmental and social requirements. But the reverse is also true. CSR long run decisions often involve a high degree of complexity. They must make the core business environmentally and socially sustainable, being, at the same time, financially sustainable themselves. In the roots of this complexity there is the fact that financial sustainability is traded in the stock market, while there are no explicit market indicators for environmental and social sustainability.

Often CSR has been regarded as a policy that reduces profits in the short run. Nevertheless, when it is well grounded in improving the corporate environmental and social sustainability in a long run perspective, CSR may become a value creation source for the society and the corporation. Then, the CSR expenses are turned into investments pursuing the goal that Husted and Allen [1] assign to CSR. This is the win-win perspective of CSR pointed out by Benabou and Tirole [2]. It agrees with the shared value approach proposed by Porter and Kramer [3] who hold that the sustainability of capitalism must come from the cooperation among corporations and their stakeholders with the aim of creating value together. Improvements in sustainability can hardly be conceived without innovation. Thus, we can say the same about CSR when we link it to sustainability. Porter and Van der Linde [4] argue that the improvement of corporate environmental sustainability is a source of innovation and competitiveness, which is stressed by Porter and Kramer, [3] and [5]. Vilanova, Lozano and Arenas [6] analyse the links between CSR, corporate learning and innovation. Adopting a long run perspective that, first, integrates innovation, sustainability and shared value and, next, undertakes the corresponding courses of action, requires quantitative support techniques that are rigorous, flexible and with a wide scope at the same time. Real options are rigorous because of their basis and flexible because they can be adapted to a wide array of situations. For these reasons they are acknowledged as a key instrument for unveiling new opportunities and valuing them. But their application to one-to-one individual decisions does not generate quantitative indicators that serve as reference points for decision analysis. Trigeorgis [7] proves the capacity of real options to value flexibility. Fichman, Keil and Tiwana [8], focusing on innovation technology (IT) project management, display the main kinds of real options embedded in IT projects. They draw the conclusion that the analysis of investment projects through real options "can guide decision makers in how to actively create and extract value". This conclusion can be extended to the analysis of CSR through real options. Husted [9] points out that often "CSR projects provide strategic flexibility in the form of real options by the indirect benefits they generate from goodwill fostered by CSR investments within the community and among consumers" [9], p. 178. Bekefi and Epstein [10] relate CSR and real options signalling that real options are the proper tool to integrate social and political risks, both clearly connected with CSR, into financial decision making. The practical applications of real options have been widely developed, as shown, among others, by Smit and Trigeorgis [11], Willigers and Hansen [12],Avanzi, Bicer, Treveille and Trigeorgis [13], and Eckhause and Herold [14].

The aim of this paper is to analyse CSR decisions from a real options perspective in order to build real options based indicators that can be used to support decision analysis on CSR, and to study in terms of binomial trees the minimum expectations scenario that makes a CSR project financially sustainable. The work is structured as follows: In the Second Section we examine the nature of CSR projects and propose a classification to facilitate their analysis. The Third Section is centred in the analysis of the capability of CSR to create value through real option ratios by applying classical options to new projects and exchange options to substitution projects. The Fourth Section focuses on the minimum expectations scenario, which is followed by an empirical illustration in the Fifth Section. The Sixth Section summarizes and discusses the results. We close the article in the Seventh Section. The calculations of the tables and figures of this paper have been performed by Mathematica. The supporting files, denoted as S_, show the corresponding Mathematica code.

## Classifying CSR Projects from the Decision Analysis Perspective

The aim of any classification consists of facilitating the thought about its elements. CSR projects may have rather different features that create a complex map because they have multiple interactions at the same time. The features that we regard as relevant for the classification are: the relationship that the projects under analysis have with the current production activities, the origin of the innovation they incorporate, the immediacy or delay of their impact on the corporate production activities, and, finally, their relationship with stakeholders. On this basis, we propose the following classification:

1. According to the relationship with the current production activities:
  - Substitution projects: they substitute the technique of a current production line for a new one that is more sustainable from the environmental or social viewpoint or both.
  - New projects: they incorporate a sustainable new production line.
  - Auxiliary projects: they contribute to make more sustainable a current production line.
2. According to the origin of the innovation they incorporate:
  - Internal projects: the innovation contained in the project has its origin in the corporate research or capabilities.
  - External projects: they do not incorporate innovation created by the corporation. Instead, the whole innovation they incorporate comes from purchasing tangible or intangible assets to other corporations.
3. According to the productive immediacy of internal projects:
  - Full projects: after their implementation they have an immediate impact on corporate production.
  - Preliminary projects: their goal is to make other projects possible if they are successful. It is the usual case of research projects.
4. According to their relationship with stakeholders:
  - Pure corporate projects: the corporation receives the whole value created by the project.
  - Shared value projects: the value created by the project is shared with some stakeholders beyond the shareholders.

**Fig 1:**
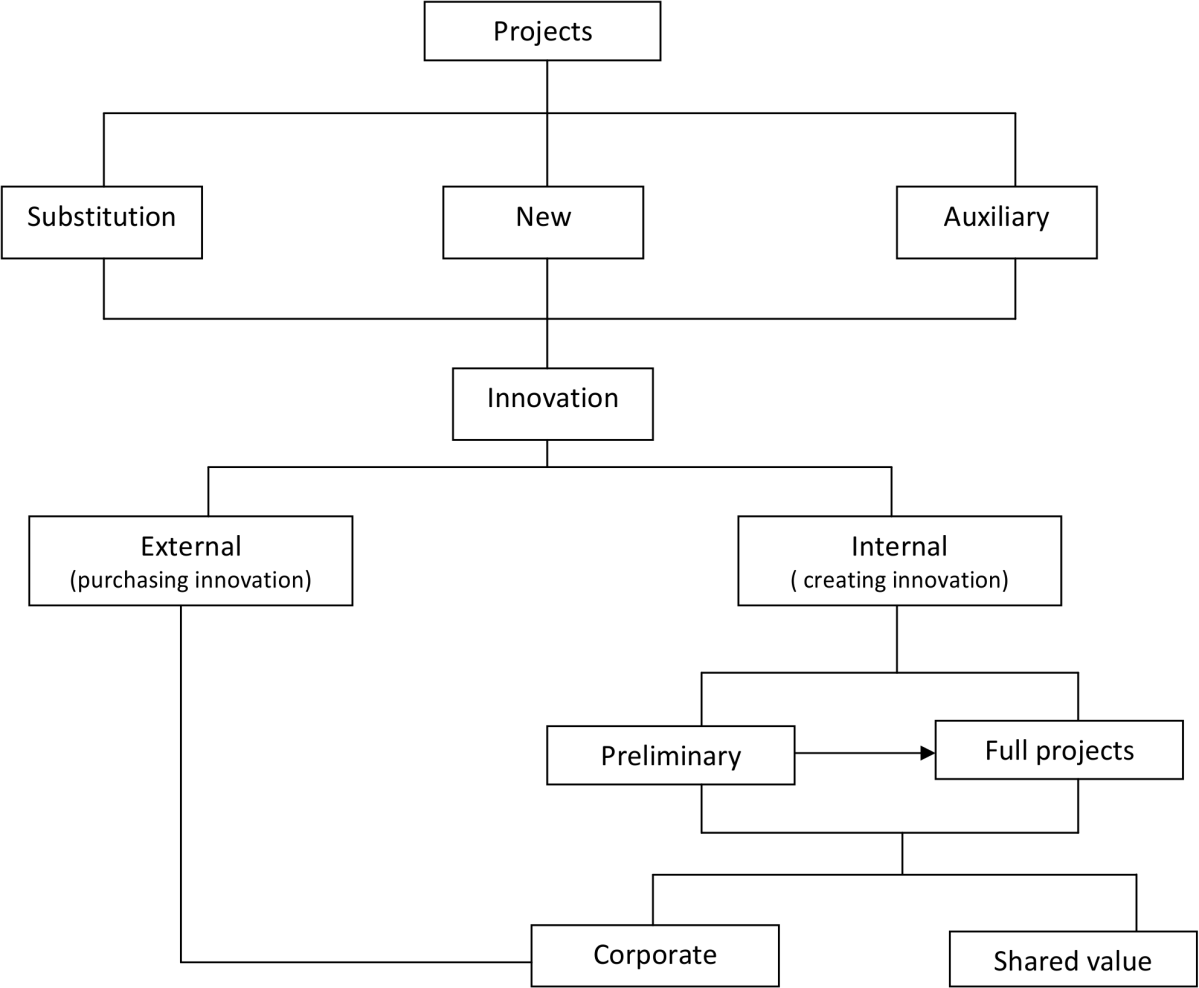
Classification of CSR projects.

We can regard the distinction among substitution, new and auxiliary projects as the basic classification. Any of these categories is at some degree connected with innovation, because innovation is in the root of CSR. Thus, anyone of these projects is, at the same time, internal or external. Internal projects are full or preliminary according to the immediacy of their impact on production. Any preliminary project enables the corporation to undertake a full project if it is successful. Internal projects may be pure corporate or shared value, while external projects as necessarily pure. Fig 1 displays a conceptual map of the relationships embedded in this classification.

The usefulness of this systematization of the central features of CSR projects lies in the fact that it contributes to choosing the best capital budgeting method to study the financial sustainability of the project, to identifying its relationships with other projects, to clarifying the corporate investment strategy, and also to helping in a better definition of the communication policy with respect to shareholders and stakeholders.

## Approaching CSR Value Creation through Real Option Ratios

As stated, real options are acknowledged as a central tool and even as *the* central tool for thinking on CSR projects. This principle is supported by the fact that a wide array of CSR projects belong to the category of preliminary projects. Obviously, the direct outcome of a real option consists of expressing the estimated value creation in monetary units. This fact arouses the question of whether this is the more productive way of exploiting the real options potential to express value creation. If the question is about a single decision, the answer is clearly positive. Nevertheless, to capture the value creation capacity of the variables involved in real options, we need a more general framework to express the consequences of their interaction. This framework is furnished by real options ratios as shown next. Ratios provide the intuition of numerical values, and, at the same time, they are scale free indicators. Furthermore, their sensitivity analysis facilitates a first quantitative approach to real option supported decision analysis. We start with the analysis of preliminary projects and, next, we study new full projects and substitution projects.

### Real Options and CSR Preliminary Projects

In this generic setting that aims to capture the value creation potentially embedded in CSR, we approach preliminary projects as European calls. What is expected from a preliminary project is the opportunity of undertaking a full project in the future. For this reason, we can regard them as calls on a future full project. Although an analysis through American options would enable us to incorporate the variability of the horizon at which the preliminary project may give way to the full project, we have opted for European calls in order to relate the variables through the Black and Scholes [15] formula. Next, we perform a sensitivity analysis for the variables involved, including horizon and volatility. Black and Scholes formula, although it is thought to value financial options, provides an adequate framework to explore interactions among the central variables of option valuation. The facts that the underlying assets of real options cannot be traded and their market values cannot be observed in a standardized way are the two main drawbacks that prevent Black and Scholes formula from being an exact tool to value real options. Nevertheless, Trigeorgis [16] points out that, according to Mason and Merton [17] and Kasanen and Trigeorgis [18], real options can be valued using financial option formulae if the goal of the analyst is to estimate their contribution to the market value of a publicly traded firm. Trigeorgis [7], chapter 3, develops a general approach to value traded and non-traded assets. Merton [19] extends the Black and Scholes formula to take into account non-tradibility and non-observability. Any of the specific formulae and methods for real option valuation requires additional information hardly available in the preliminary stages of the analysis. Besides, an exact real option valuation implies in almost all cases a customized model. The ultimate aim of our approach is to give a quantitative support to decision analysis on CSR, making this intangible asset to look as tangible as possible by helping decision makers to seek for the investment beyond the expense in any case. In this context, the call expresses the value of the opportunity created by the CSR project while the value of the asset is the Gross Present Value (GPV) of the future investment project and the strike (K) price is its capital outlay. As known, the remaining variables of the formula are volatility (standard deviation, *σ*), time (T) and the risk free interest rate (*r*). The necessary condition for the financial sustainability of a preliminary project is that the gross value it creates, i.e. the option value, is greater than the capital outlay required for undertaking this preliminary project. Thus, the NPV created by the preliminary project (PNPV) can be written as the difference between the option value (*c*) and the preliminary capital outlay (PI):

PNPV=c−PI (1)

To analyse the value creation potential of preliminary projects through real option ratios we transform the Black and Scholes formula by taking, respectively, as the dependent variable the ratio between the call and the strike price first, and, next, the ratio between the call and the underlying asset. These changes are straightforward because they simply require dividing both sides of the formula by the aforementioned variables. We call Opportunity Ratio (OPR) the quotient between the call and the strike price. New Project Ratio (NPR) is how we denominate the quotient between the call and the GPV of the future full project. The former expresses the present value of the opportunity to invest 1$ in the full project at the option maturity, i.e. the maximum amount that is worth investing in the preliminary project for each dollar of the future capital outlay. The latter expresses the same amount for each dollar of the GPV of the future full project.

In order to obtain the OPR formula, we divide both sides of the Black and Scholes formula for calls by the strike price. Applied to this case, the formula can be written as:

$$c=GPV\cdot N\left(d_{1}\right)-K\cdot e^{-r\cdot T}\cdot N\left(d_{2}\right)$$ (2)

where:

$$d_{1}=\frac{\text{ln}\left(\frac{GPV}{K}\right)+\left(r+\frac{\sigma^{2}}{2}\right)T}{\sigma \cdot \sqrt{T}}$$ (3)

$$d_{2}=d_{1}-\sigma \cdot \sqrt{T}$$ (4)

Thus, after defining the GPV index (*a*) as the quotient between the asset and the strike price (*GPV* / *K*), we can write the *OPR* formula as:

$$OPR=a\cdot N\left(d_{1}\right)-e^{-rT}N\left(d_{2}\right)$$ (5)

Where:

$$d_{1}=\frac{\text{ln}\;a+\left(r+\frac{\sigma^{2}}{2}\right)T}{\sigma \cdot \sqrt{T}}$$ (6)

The New Project Ratio (NPR) is obtained by dividing both sides of (2) by the GPV:

$$NPR=N\left(d_{1}\right)-\frac{1}{a}N\left(d_{2}\right)$$ (7)

An alternate approach, that we have discarded, could consist of defining the OPR as the quotient between the call the present value of the strike price (*Ke* ^−*rT*^). Nevertheless, this approach would prevent from developing the sensitivity analysis for the interest rate and the horizon because it eliminates them from the formula.

We can observe that the OPR and the NPR are functions of the GPV index, volatility, time and risk free interest rate. On this basis, we can ask and answer questions that relate some of the central issues in CSR decisions about new projects:

- How many dollars can be invested in a CSR project for each dollar of the amount to be invested in the full project in the future? (OPR).
- How many dollars can be invested in a CSR preliminary project for each dollar of the full project GPV? (NPR).
- Which is the value of the GPV index that justifies a specific value of the OPR?
- How many years an opportunity must last in order to justify the investment that creates it?

Questions (a) and (b) are answered through the Black and Scholes formula turned into ratios. To answer questions (c) and (d) we need to apply numerical methods. The answers to these questions can be regarded as a sensitivity analysis and as a breakeven analysis at the same time A sensitivity analysis because we can obtain their answers for different sets of the independent variables. A breakeven analysis because the answer to each question gives a threshold value over which (OPR and NPR) or under which (GPV index and time) the preliminary project is not financially sustainable. Concretely, if the amount to be invested now in a CSR project for each dollar to be invested in the future project is greater than the OPR threshold value, the CSR project is not financially sustainable. If a CSR project shows a GPV index lower than its threshold, the project is not financially sustainable as well. Below we obtain the minimum volatility required by financial sustainability. Sensitivity analysis has a relevant role in real option analysis due to the elusive nature of the values of its variables that hardly have market references. CSR projects make this feature even more relevant. In Tables 1–4 and their corresponding figures we build up a sensitivity analysis based on questions (a), (b), (c) and (d), while Table 5 focuses on the sensitivity of minimum volatility. These tables create a framework that enables a straightforward approximation to the quantitative impact of CSR decisions. For a wide array of values of the independent variables, they facilitate the corresponding maximum values of the OPR, the NPR, and the minimum values of GPV index, horizon and volatility.

**Table 1:** Opportunity Ratio.

*a*	0.5	0.75	1	1.25	1.5
*T*	*σ* =0.10				
3	0.0000	0.0064	0.1001	0.3122	0.5585
5	0.0002	0.0206	0.1407	0.3530	0.5962
10	0.0051	0.0679	0.2267	0.4446	0.6850
15	0.0189	0.1191	0.3006	0.5242	0.7646
20	0.0393	0.1694	0.3663	0.5946	0.8359
25	0.0638	0.2172	0.4253	0.6576	0.8999
50	0.1965	0.4114	0.6467	0.8899	1.1365
	*σ* =0.15				
3	0.0004	0.0228	0.1321	0.3279	0.5627
5	0.0034	0.0494	0.1800	0.3767	0.6063
10	0.0230	0.1151	0.2757	0.4793	0.7059
15	0.0513	0.1750	0.3535	0.5638	0.7916
20	0.0823	0.2289	0.4201	0.6362	0.8660
25	0.1135	0.2775	0.4784	0.6992	0.9312
50	0.2480	0.4594	0.6868	0.9226	1.1630
	*σ* =0.20				
3	0.0031	0.0443	0.1646	0.3517	0.5751
5	0.0127	0.0814	0.2202	0.4096	0.6283
10	0.0490	0.1621	0.3271	0.5249	0.7426
15	0.0887	0.2293	0.4103	0.6157	0.8360
20	0.1269	0.2868	0.4793	0.6909	0.9143
25	0.1622	0.3368	0.5381	0.7549	0.9813
50	0.2964	0.5110	0.7374	0.9705	1.2078
	*σ* =0.30				
3	0.0188	0.0934	0.2294	0.4103	0.6195
5	0.0451	0.1480	0.3004	0.4863	0.6938
10	0.1099	0.2529	0.4291	0.6265	0.8379
15	0.1653	0.3316	0.5227	0.7291	0.9458
20	0.2119	0.3943	0.5960	0.8095	1.0309
25	0.2513	0.4456	0.6554	0.8746	1.1001
50	0.3768	0.6029	0.8350	1.0709	1.3093
	*σ* =0.50				
3	0.0763	0.1966	0.3551	0.5393	0.7411
5	0.1309	0.2777	0.4527	0.6461	0.8521
10	0.2313	0.4139	0.6130	0.8227	1.0397
15	0.2995	0.5018	0.7152	0.9356	1.1608
20	0.3482	0.5633	0.7862	1.0139	1.2452
25	0.3838	0.6078	0.8374	1.0706	1.3062
50	0.4670	0.7101	0.9545	1.1999	1.4458

Table 1 shows the values of the Opportunity Ratio per the indicated values of the *GPV* index (*a*), horizon (*T*) and volatility (*σ*). The risk free interest rate is set up to 2%.

**Table 2:** New Project Ratio.

*a*	0.5	0.75	1	1.25	1.5
*T*	*σ* = 0.10				
3	0.0000	0.0085	0.1001	0.2498	0.3723
5	0.0004	0.0274	0.1407	0.2824	0.3975
10	0.0102	0.0905	0.2267	0.3557	0.4566
15	0.0377	0.1588	0.3006	0.4194	0.5097
20	0.0786	0.2258	0.3663	0.4757	0.5573
25	0.1276	0.2896	0.4253	0.5260	0.6000
50	0.3931	0.5486	0.6467	0.7119	0.7577
	*σ* = 0.15				
3	0.0009	0.0304	0.1321	0.2623	0.3751
5	0.0069	0.0658	0.1800	0.3014	0.4042
10	0.0460	0.1535	0.2757	0.3835	0.4706
15	0.1026	0.2333	0.3535	0.4511	0.5277
20	0.1647	0.3052	0.4201	0.5089	0.5773
25	0.2271	0.3700	0.4784	0.5593	0.6208
50	0.4960	0.6125	0.6868	0.7380	0.7753
	*σ* = 0.20				
3	0.0063	0.0591	0.1646	0.2813	0.3834
5	0.0254	0.1086	0.2202	0.3277	0.4189
10	0.0979	0.2161	0.3271	0.4199	0.4951
15	0.1774	0.3058	0.4103	0.4925	0.5573
20	0.2537	0.3825	0.4793	0.5527	0.6096
25	0.3244	0.4491	0.5381	0.6039	0.6542
50	0.5927	0.6813	0.7374	0.7764	0.8052
	*σ* = 0.30				
3	0.0376	0.1246	0.2294	0.3283	0.4130
5	0.0901	0.1974	0.3004	0.3890	0.4625
10	0.2198	0.3371	0.4291	0.5012	0.5586
15	0.3307	0.4422	0.5227	0.5833	0.6305
20	0.4239	0.5258	0.5960	0.6476	0.6873
25	0.5026	0.5942	0.6554	0.6997	0.7334
50	0.7536	0.8038	0.8350	0.8568	0.8729
	*σ* = 0.50				
3	0.1527	0.2621	0.3551	0.4315	0.4941
5	0.2617	0.3703	0.4527	0.5169	0.5681
10	0.4626	0.5519	0.6130	0.6582	0.6932
15	0.5990	0.6691	0.7152	0.7485	0.7739
20	0.6963	0.7510	0.7862	0.8112	0.8301
25	0.7677	0.8104	0.8374	0.8564	0.8708
50	0.9340	0.9468	0.9545	0.9599	0.9639

Table 2 shows the values of the New Project Ratio per the indicated values of the GPV index (*a*), volatility (*σ*), and horizon (*T*). The risk free interest rate is set up to 2%.

**Table 3:** GPV index.

σ	0.1	0.2	0.3	0.4	0.5
*r*	T = 1				
0.02	1.2298	1.2144	1.1771	1.1265	1.0695
0.03	1.2200	1.2051	1.1685	1.1188	1.0626
0.05	1.2009	1.1869	1.1517	1.1036	1.0490
0.07	1.1821	1.1690	1.1352	1.0887	1.0356
0.09	1.1637	1.1514	1.1190	1.0739	1.0223
0.1	1.1546	1.1427	1.1110	1.0667	1.0158
	T = 2				
0.02	1.2074	1.1674	1.0982	1.0179	0.9363
0.03	1.1887	1.1505	1.0835	1.0053	0.9256
0.05	1.1523	1.1175	1.0547	0.9808	0.9048
0.07	1.1172	1.0857	1.0270	0.9570	0.8846
0.09	1.0835	1.0549	1.0001	0.9339	0.8649
0.1	1.0672	1.0400	0.9870	0.9226	0.8553
	T = 3				
0.02	1.1838	1.1226	1.0313	0.9336	0.8400
0.03	1.1568	1.0992	1.0118	0.9176	0.8271
0.05	1.1051	1.0542	0.9742	0.8868	0.8020
0.07	1.0562	1.0113	0.9383	0.8572	0.7779
0.09	1.0100	0.9707	0.9041	0.8290	0.7548
0.1	0.9879	0.9511	0.8875	0.8153	0.7436
	T = 4				
0.02	1.1601	1.0813	0.9740	0.8654	0.7660
0.03	1.1255	1.0523	0.9507	0.8471	0.7516
0.05	1.0601	0.9972	0.9063	0.8118	0.7239
0.07	0.9994	0.9456	0.8645	0.7785	0.6976
0.09	0.9430	0.8973	0.8251	0.7470	0.6726
0.1	0.9164	0.8744	0.8063	0.7319	0.6606
	T = 5				
0.02	1.1367	1.0432	0.9242	0.8088	0.7069
0.03	1.0952	1.0094	0.8979	0.7887	0.6915
0.05	1.0176	0.9459	0.8481	0.7504	0.6623
0.07	0.9468	0.8873	0.8019	0.7146	0.6347
0.09	0.8822	0.8332	0.7590	0.6811	0.6089
0.1	0.8521	0.8078	0.7387	0.6652	0.5966

Table 3 shows the values of the GPV index for the given values of standard deviation, risk-free interest rate, and time. The Opportunity Ratio is set up to 0.25.

**Table 4:** Horizon.

σ	0.1	0.2	0.3	0.4	0.5
r	*a* = 0.5				
0.02	61.5407	39.8998	24.8221	16.2054	11.2004
0.03	43.3122	31.6593	21.4134	14.6901	10.4576
0.05	27.0228	22.3140	16.7675	12.3642	9.2286
0.07	19.5635	17.1726	13.7534	10.6640	8.2538
0.09	15.3051	13.9285	11.6427	9.3678	7.4619
0.1	13.7975	12.7190	10.8087	8.8290	7.1193
	*a* = 0.75				
0.02	28.6126	16.7129	9.8407	6.2464	4.2508
0.03	20.6202	13.6885	8.7091	5.7699	4.0243
0.05	13.1588	10.0388	7.0806	5.0068	3.6372
0.07	9.6267	7.9141	5.9637	4.4222	3.3184
0.09	7.5745	6.5238	5.1493	3.9597	3.0511
0.1	6.8415	5.9948	4.8195	3.7628	2.9330
	*a* = 1				
0.02	11.5058	6.2407	3.5280	2.1935	1.4759
0.03	8.4131	5.2319	3.1818	2.0544	1.4115
0.05	5.4310	3.9480	2.6598	1.8233	1.2983
0.07	3.9888	3.1641	2.2842	1.6390	1.2021
0.09	3.1432	2.6358	2.0007	1.4885	1.1191
0.1	2.8400	2.4314	1.8835	1.4231	1.0818
	*a* = 1.1				
0.02	6.6228	3.5367	1.9747	1.2197	0.8177
0.03	4.8395	2.9841	1.7915	1.1474	0.7845
0.05	3.1107	2.2662	1.5099	1.0256	0.7257
0.07	2.2756	1.8205	1.3035	0.9268	0.6750
0.09	1.7879	1.5173	1.1456	0.8452	0.6309
0.1	1.6136	1.3995	1.0798	0.8094	0.6108
	*a* = 1.2				
0.02	2.3159	1.3069	0.7336	0.4531	0.3036
0.03	1.6426	1.0938	0.6646	0.4263	0.2914
0.05	1.0178	0.8149	0.5571	0.3806	0.2696
0.07	0.7314	0.6423	0.4773	0.3431	0.2506
0.09	0.5697	0.5262	0.4160	0.3117	0.2339
0.1	0.5128	0.4816	0.3904	0.2980	0.2263

Table 4 shows the values of the horizon for the given values of the standard deviation, the risk-free interest rate and the GPV index. The Opportunity Ratio is set up to 0.25.

**Table 5:** Minimum volatitlity.

PIK = 0.10						
*a*↓ T→	2	3	4	5	6	7
0.3	1.1442	0.9283	0.7988	0.7099	0.6438	0.5921
0.5	0.7070	0.5702	0.4877	0.4307	0.3880	0.3545
0.6	0.5656	0.4542	0.3866	0.3397	0.3045	0.2766
0.75	0.3940	0.3128	0.2630	0.2281	0.2015	0.1802
0.9	0.2454	0.1890	0.1535	0.1276	0.1072	0.0900
PIK = 0.20						
0.3	1.8198	1.4805	1.2774	1.1384	1.0353	0.9549
0.5	1.0665	0.8639	0.7421	0.6583	0.5960	0.5471
0.6	0.8633	0.6972	0.5971	0.5281	0.4765	0.4359
0.75	0.6315	0.5067	0.4310	0.3784	0.3388	0.3075
0.9	0.4438	0.3516	0.2948	0.2548	0.2243	0.1997
PIK = 0.25						
0.3	2.3458	1.9105	1.6503	1.4723	1.3406	1.2379
0.5	1.2608	1.0228	0.8799	0.7818	0.7088	0.6517
0.6	1.0184	0.8241	0.7072	0.6267	0.5667	0.5196
0.75	0.7530	0.6062	0.5174	0.4558	0.4097	0.3733
0.9	0.5456	0.4350	0.3675	0.3203	0.2844	0.2558
PIK = 0.30						
0.5	1.4772	1.1997	1.0335	0.9193	0.8347	0.7685
0.6	1.1840	0.9596	0.8248	0.7320	0.6631	0.6090
0.75	0.8793	0.7095	0.6071	0.5364	0.4835	0.4418
0.9	0.6501	0.5208	0.4422	0.3874	0.3462	0.3134
PIK = 0.50						
0.6	2.1259	1.7302	1.4936	1.3315	1.2115	1.1179
0.75	1.4791	1.2007	1.0337	0.9191	0.8340	0.7674
0.9	1.1153	0.9023	0.7741	0.6858	0.6199	0.5682
PIK = 0.75						
0.8	2.6908	2.1921	1.8941	1.6902	1.5394	1.4219
0.9	1.9807	1.6111	1.3899	1.2383	1.1260	1.0383

Table 5 shows the values of the minimum volatilities for the indicated values of GPV index, horizon and PIK ratio. The risk free interest rate is set out at 2%.

**Fig 2:**
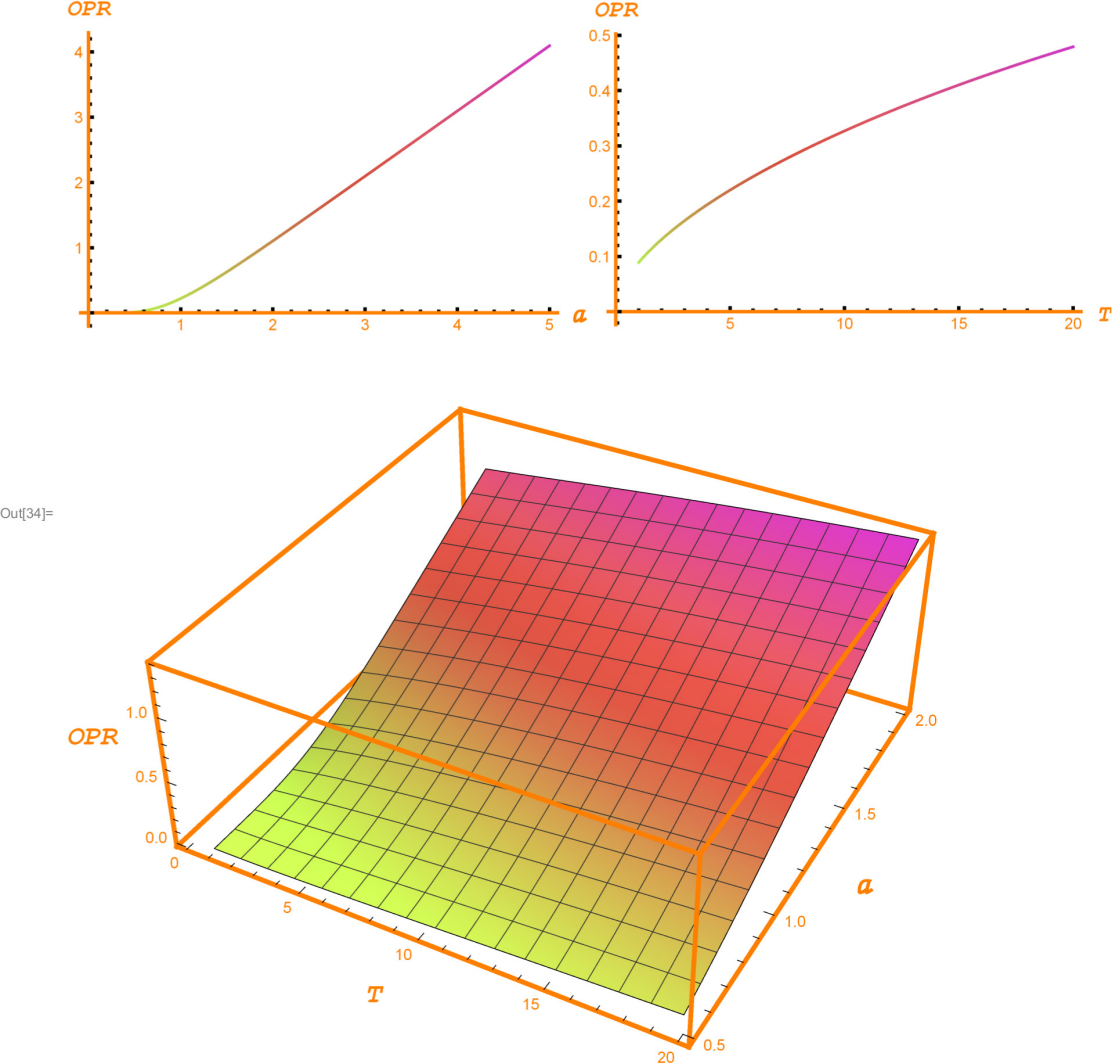
Opportunity Ratio. The two bi-dimensional plots depict the evolution of the Opportunity Ratio (OPR) according, respectively, to the GPV index (*a*) and the horizon (*T*). In the left hand side plot, the horizon is set up to 5 years, and, in the right hand side plot the GPV index is set up to 1. The three-dimensional plot shows the conjoint evolution of the Opportunity Ratio with respect to the horizon and the GPV index. The risk-free interest rate is set up to 2% and volatility to 20%.

Table 1 shows the values of the OPR for the given values of the GPV index, the volatility and a 2% risk free interest rate. For instance, assuming a volatility of 20%, a 5 year horizon and a GPV index equal to 1, we obtain an OPR of 22%. Thus, under these assumptions, the maximum amount to be spent to create the opportunity of investing 100$ in the full in five years is 22$. Fig 2 shows a graphical depiction of Table 1.

**Fig 3:**
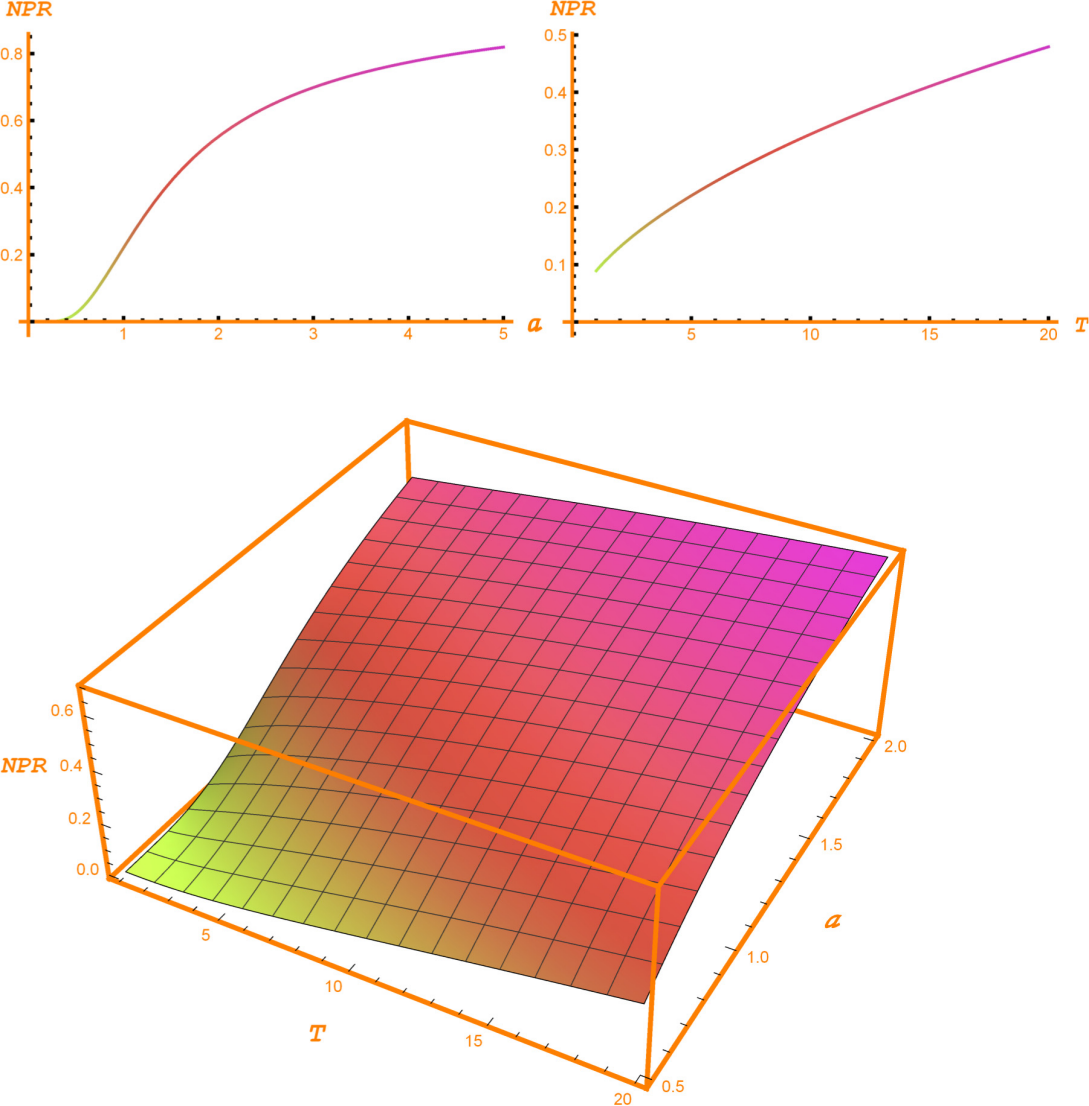
New Project Ratio. The two bi-dimensional plots depict the evolution of the New Project Ratio (NPR) according, respectively, to the GPV index (*a*) and horizon (*T*). In the left hand side plot, the horizon is set up to 5 years, and, in the right hand side plot the GPV index is set up to1. The three-dimensional plot shows the conjoint evolution of the New Project Ratio with respect to the horizon and the GPV index.

Table 2 shows the values of the NPR for the same values of GPV index, volatility, interest rate and horizon. For a GPV index equal to 1, the OPR and the NPR have the same value. For 20% volatility, 5 years horizon, 2% interest rate and a GPV index of 0.75, the NPR is 0.1086. In this case, the new project has a negative NPV equal to the 25% of its capital outlay. Nevertheless, this negative value can change into a positive one in the future when the decision whether to start the project is to be made. For this reason, it is worth investing in CSR now the aforementioned 10.86% to create this opportunity. It is like purchasing an out-of-the-money financial option. Fig 3 depicts Table 2.

**Fig 4:**
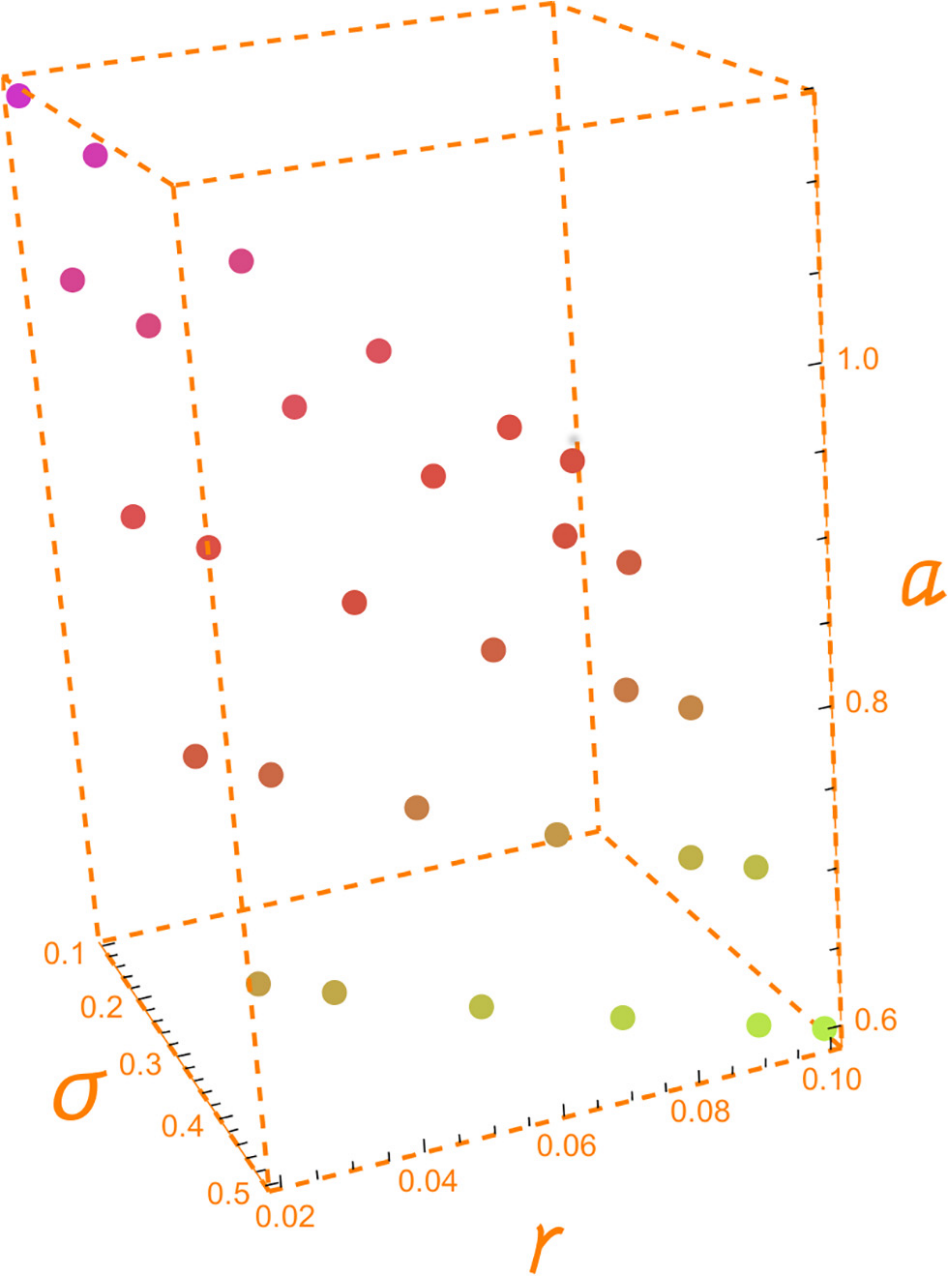
GPV index. Fig 4 depicts the values of the GPV index shown in Table 3 for a five year horizon in the last six rows of this table. Each one of the five sets of six decreasing points corresponds to each one the five columns under T = 5 for the set of volatilities: 0.10, 0.20, 0.30, 0.40 and 0.50. We can observe how the required GPV index decreases as volatility and interest rate increase.

Table 3 displays the values of the GPV index for an OPR of 25%, horizons from 1 to 5 years, five values of the volatility from 10% to 50%, and the indicated values of the risk free interest rate (2%, 3%, 5%, 7%, 9% and 10%). For a 5 year horizon, a volatility of 20% and a risk free interest rate of 2%, we need a GPV index approximately equal to 1.043185 in order to have an OPR of 25%. The GPV index becomes 0.92 for a volatility of 30%. These are the required GPV indices that make reasonable to spend 25$ in the creation of the opportunity to undertake the project for each 100$ of the future capital outlay and the given values of horizon, volatility and interest rate. The 0.92 GPV index corresponds to a negative ratio between the NPV and the future capital outlay (-0.08). It is again a case of an out-of-the-money option. Fig 4 illustrates Table 3.

**Fig 5:**
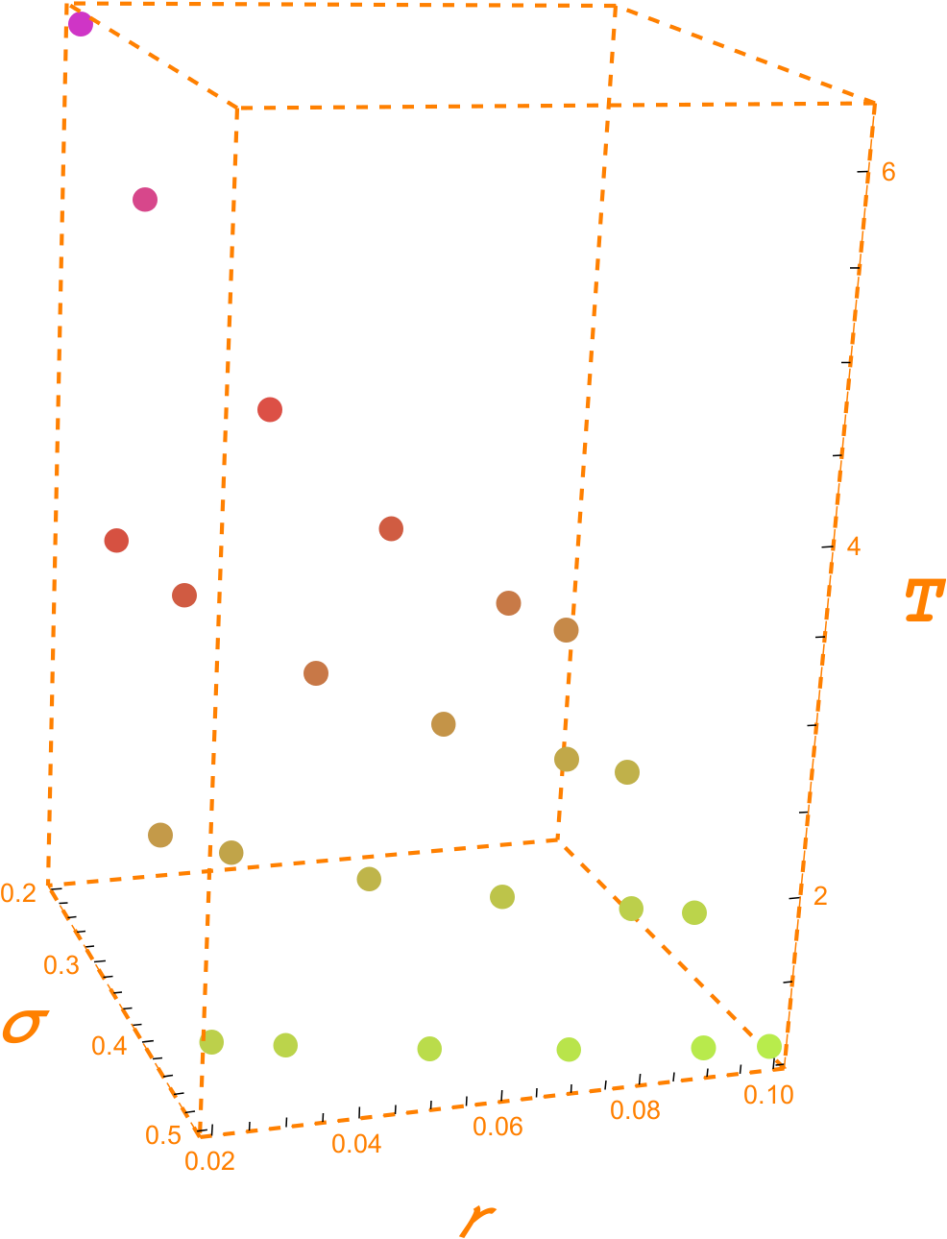
Horizon. Fig 5, following Table 4, shows the evolution of the minimum necessary horizon at which an opportunity must last in order to justify an Opportunity Ratio of 25%. The GPV index is set up to 1 (i.e. *a* = 1); the risk free interest rate values are 2%, 3%, 5%, 7%, 9% and 10%, while the volatility values are0.20, 0.30, 0.40 and 0.50. The column for 0.10 volatility is not included in the table for space reasons. We can observe how the minimum required horizon decreases as volatility and interest rate increase.

In Table 4, we calculate the horizons that fit with an OPR of 25% and the indicated values of GPV index, volatility and risk free interest rate. These horizons are the minimum number of years that the opportunity to start the new project must last so that it makes sense to spend the 25% of the future capital outlay to create the opportunity. The minimum horizon that makes feasible the investment in the call option can be regarded as: a) the minimum maturity for an European call, or b) the minmum horizon that makes financially feasible, although not optimal, the early exercise of an American call on an asset that does not generate explicit yields asit is the case of the preliminary CSR project. For a risk free interest rate of 3%, a volatility of 20% and a GPV index equal to 1 at the present moment, the opportunity must last for a minimum period of approximately 5 years and 3 months (5.23). Fig 5 depicts the values of Table 4 for a GVP index equal to 1.

The preliminary project is financially sustainable provided it fulfils the classical financial sustainability condition of having a non-negative NPV. As it stems from (1), this condition means that the minimum acceptable value of the call is the capital outlay required by the preliminary investment (*c* = *PI*). The minimum acceptable volatility is, thus, the one that leads to this equality. In order to express the financial sustainability condition of the preliminary investment in ratio terms, let us define the Preliminary Investment/Strike Price Ratio (PIK) as the quotient between the capital outlays required by the preliminary project (PI) and the full project:

$$PIK=\frac{PI}{K}$$ (8)

Hence, after equating the call to the preliminary investment (*c* = *PI*) and dividing both sides of (1) by the strike price, we can write:

$$PIK=a\;N\left(d_{1}\right)-e^{-r\cdot T}\cdot N\left(d_{2}\right)$$ (9)

**Fig 6:**
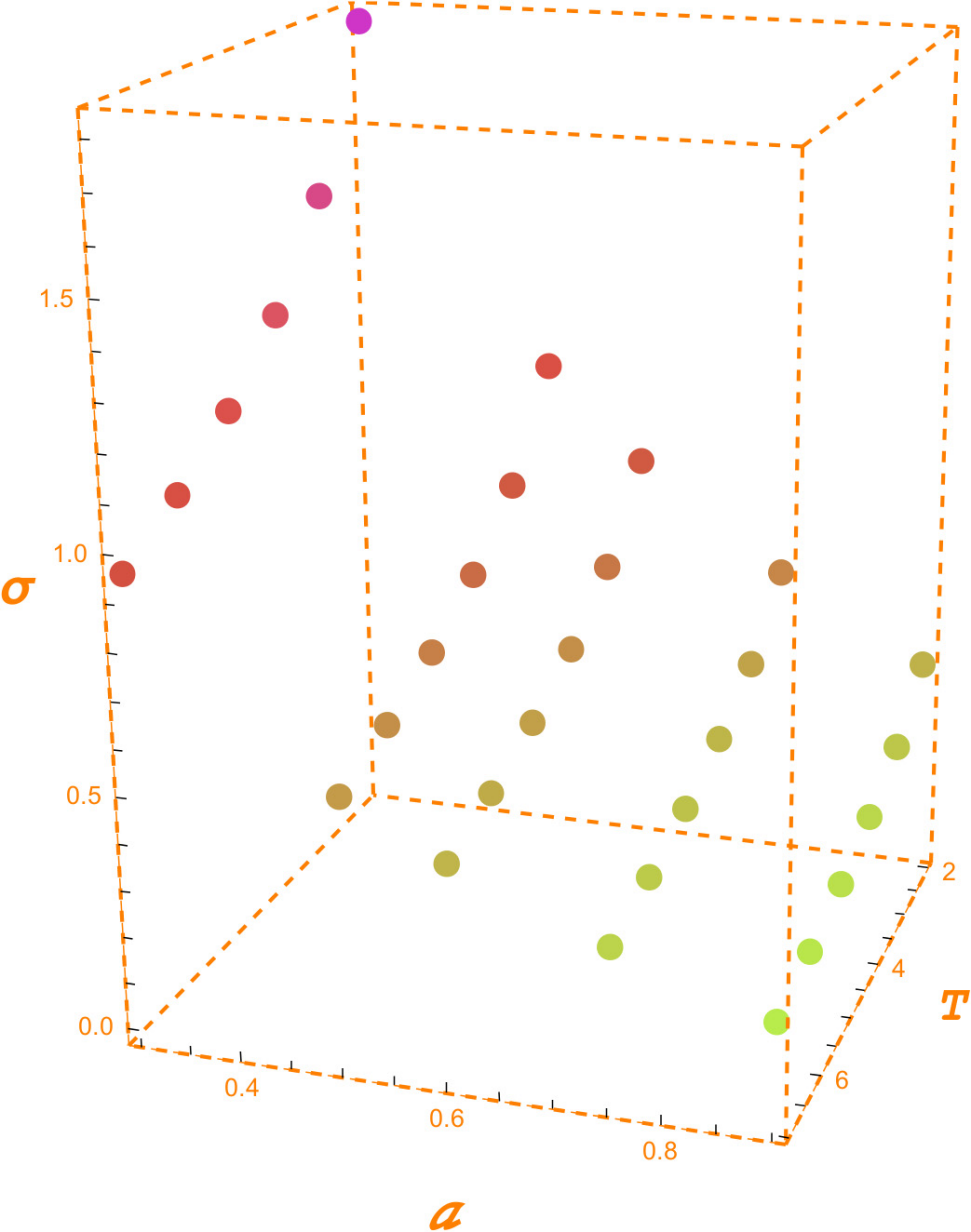
Minimum volatility. Fig 6, following Table 5, shows the evolution of the minimum volatility for a GPV indexes 0.3, 0.5, 0.6, 0.75 and 0.9, and horizons from 2 to 7 years. The PIK ratio is set out at 0.20. We can observe how the minimum volatility decreases as the GPV index and horizon increase.

Solving this equation by numerical methods we obtain the minimum volatility required to undertake the preliminary project. Table 5 presents the minimum volatilities required by different PIK ratios, GPV indices, and horizons. The values shown in this table can be regarded as the breakeven volatilities for the financial sustainability of the project. Nicholis, Lewis, Zhang and Jiang [20] introduce the breakeven volatily in real options analysis. Fig 6 depicts Table 5.

The reading of Table 5 requires to remember that options' values are an increasing function of volatility. For a risk free interest rate of 2%, a 5 years horizon and a GPV index of 0.60, we need a 33.97% volatility to obtain a 10% PIK ratio. This volatility rises to 52.30% and 73.20% if we seek a PIK ratio of, respectively, 20% and 30%. In other words, these are the minimum volatilities that justify to invest the 10%, 20% and 30% of the full project capital outlay to start the preliminary project.

### Real Options and CSR New Full Projects

The direct approach to full projects is the traditional discounted cash flow technique. If the outcome is a positive NPV, the project is undertaken, while if the outcome is a negative NPV the project is rejected. Nevertheless, often is worth examining if there are value creation opportunities embedded in the project that initially has a negative NPV. In effect, it could be the case that an excellent project from the environmental or social sustainability point of view presents a negative NPV. The immediate decision that stems from the financial goal of shares value maximization is to reject the project. Nevertheless, real options open the possibility of reconsidering it. Deeping into the analysis of the project leads to study whether there are unveiled opportunities in it that can be evaluated through option theory. We call them embedded real options. Although they could be calls when the opportunity consists of expanding the project, they also could be puts if the embedded opportunity consists of abandoning the project, i.e. they are abandonment options. In fact, CSR full projects may contain any of the options depicted in the real options literature as, for instance, in Trigeorgis [7], and Copeland and Antikarov [21]. From the capital outlay perspective, there is a central difference between a preliminary project and a real option embedded in a full project. To have access to the future choices created by a preliminary project requires to undertake this project by spending the corresponding capital outlay. Conversely, the capital outlay required by embedded real options is also embedded in the capital outlay necessary for undertaking the future project. In other words, to have access to the future choices of embedded real options does not require a capital outlay additional to the one of the full project. Examining this scenario in terms of the OPR, the project becomes financially sustainable if the addition of this ratio (*c* / *K*) with the GPV index (*GPV* / *K*) is greater than 1.

### Real Options Applied to CSR Substitution Projects

Substitution projects can be approached through exchange options. Financially, an exchange option enables its holder to exchange one asset for another. From the call viewpoint, the asset to be received, if the option is exercised, is the underlying asset, while the asset to be delivered is the strike price. When a corporation, through a preliminary investment, puts the basis to undertake a substitution project, it creates the opportunity to exchange a present state for a new state. Exchange options are valued through Margrabe formula [22]. Its independent variables are the present values of the asset to be received and the asset to be delivered, maturity and the differential volatility between the continuously compounded rates of return of both assets, henceforth differential volatility. Interest rate is excluded. Bouasker and Prigent [23] generalize the Margrabe formula for different production models. Copeland and Antikarov [21], chp. 6, apply binomial trees to the analysis of switching from one mode of operation to another. Let us centre in the substitution of a production technique that must be studied from the viewpoint of costs. Then, in the exchange option, the asset to be received consists of the present value of the future savings that the corporation would obtain by abandoning the technique that is currently using. We name them Savings Present Value (SPV). The asset to be delivered is, in turn, the present value of the costs of the new technique to be implemented, that we name New Technique Costs (NTC). The former incorporates the salvage value of the old technique and the latter the capital outlays required by the new one.

Any CSR substitution project focused on production techniques is aimed at improving their environmental or their social sustainability. Often, the creation of such opportunities requires investing in a preliminary project that may be successful or not. The motivation that triggers these preliminary investments is the concern that the corporation has with sustainability, i.e. its CSR. The question that the analysis of the financial sustainability of these CSR investments must answer is: Which is the maximum percentage of the SPV that can be spent in order to create the opportunity of developing a more sustainable production technique?

The answer to this question can be obtained by dividing both sides of the Margrabe formula by the SPV. Then, the dependent variable turns out to be the quotient between the value of the opportunity, i.e. the exchange option value, and the SPV. We name it Opportunity Savings Ratio (OSR):

$$OSR=\frac{X}{SPV}$$ (10)

The independent variables are time, differential volatility and the ratio between the NTC and the SPV, that we call Cost Savings Ratio (CSV):

$$CSV=\frac{NTC}{SPV}$$ (11)

Therefore, this approach balances the OSR against the CSV. For any given value of the CSV, the OSR is the maximum percentage we are looking for. Denoting by RC the asset to be received and by DL the asset to be delivered, the formula is:

$$X=RC\cdot N\left(d_{{}_{1}}^{'}\right)-DL\cdot N\left(d_{2}^{'}\right)$$ (12)

$$d_{{}_{1}}^{'}=\frac{\text{ln}\left(\frac{RC}{DL}\right)+\frac{\upsilon^{2}}{2}\cdot T}{\upsilon \cdot \sqrt{T}}$$ (13)

$$d_{{}_{2}}^{'}=d_{{}_{1}}^{'}-\upsilon \cdot \sqrt{T}$$ (14)

Where the differential volatility is:

$$\upsilon =\sqrt{\sigma_{RC}^{2}+\sigma_{DL}^{2}-2\cdot \rho \cdot \sigma_{RC}\cdot \sigma_{DL}}$$ (15)

*σ*_*RC*_ and *σ*_*DL*_ stand for the volatilities of both assets, while *ρ* denotes their correlation.

Substituting *RC* by *SPV* and *DL* by *NTC*, the exchange option for substitution project turns out to be:

$$X=SPV\cdot N\left(d_{{}_{1}}^{'}\right)-NTC\cdot N\left(d_{{}_{2}}^{'}\right)$$ (16)

$$d_{{}_{1}}^{'}=\frac{\text{ln}\frac{SPV}{NTC}+\frac{\upsilon^{2}}{2}\cdot T}{\upsilon \cdot \sqrt{T}}=\frac{\text{ln}\frac{1}{CSV}+\frac{\upsilon^{2}}{2}\cdot T}{\upsilon \cdot \sqrt{T}}$$ (17)

Where the CSV, as we have defined in (11), stands for the quotient *NTC* / *SPV*.

Dividing both sides of (16) by the SPV, we obtain the formula for the OSR:

$$OSR=N\left(d_{{}_{1}}^{'}\right)-CSV\cdot N\left(d_{{}_{2}}^{'}\right)$$ (18)

A complementary insight into the financial sustainability of CSR substitution decisions can be obtained by reversing the roles of OSR and CSV ratios in the previous analysis. Then, the question to be asked is: Which is the maximum CSV that justifies a given value of the OSR? The answer requires to solve the Margrabe formula for CSV through numerical methods.

**Fig 7:**
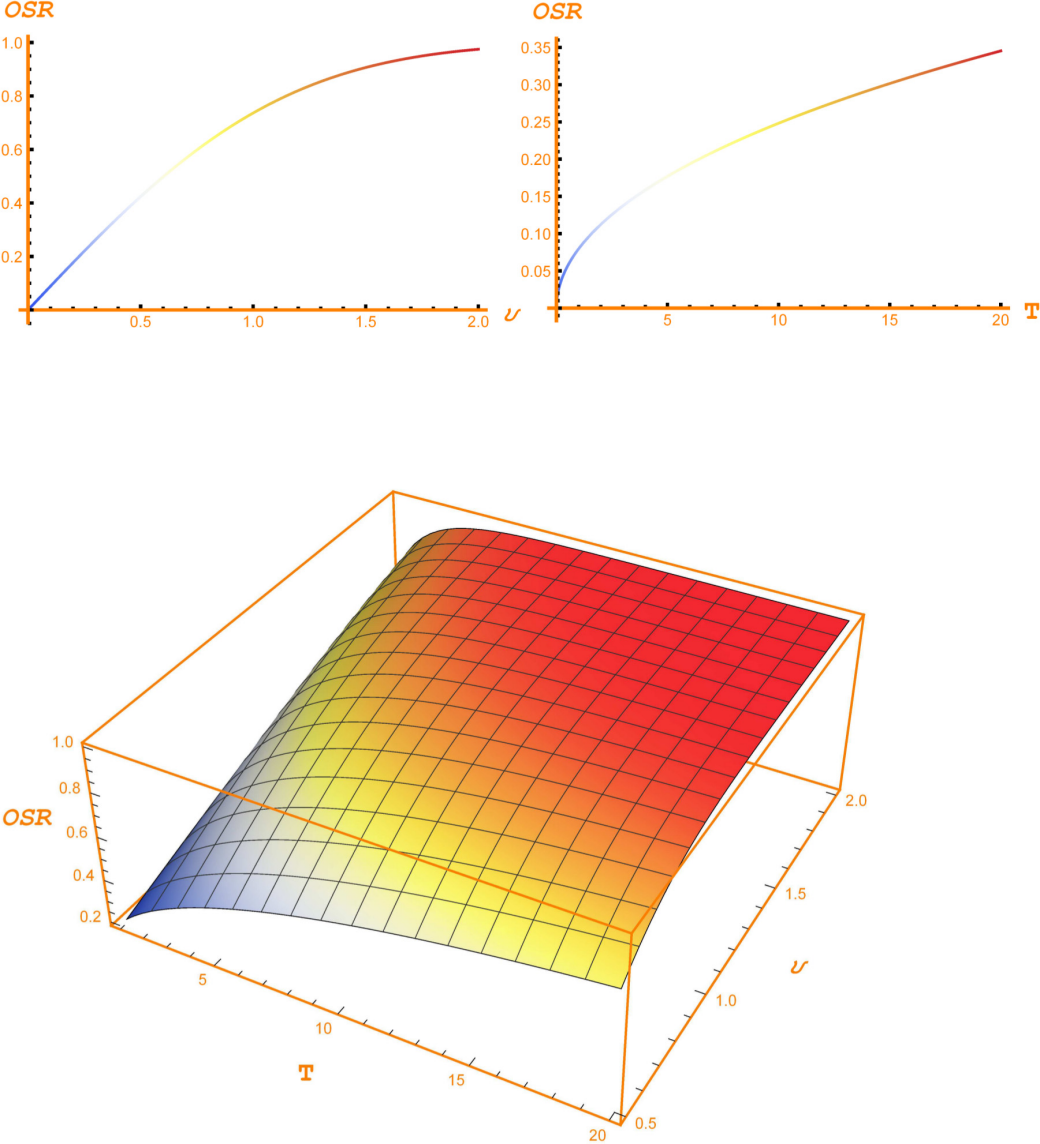
Opportunity Savings Ratio. The two bi-dimensional plots depict the evolution of the Opportunity Savings Ratio (OPR) according, respectively, to differential volatility (*υ*) and horizon (*T*). In the left hand side plot, the horizon is set up to 5 years, and, in the right hand side plot the differential volatility is set up to 0.20. The three-dimensional plot shows the conjoint evolution of the New OSR with respect to the horizon and differential volatility.

**Fig 8:**
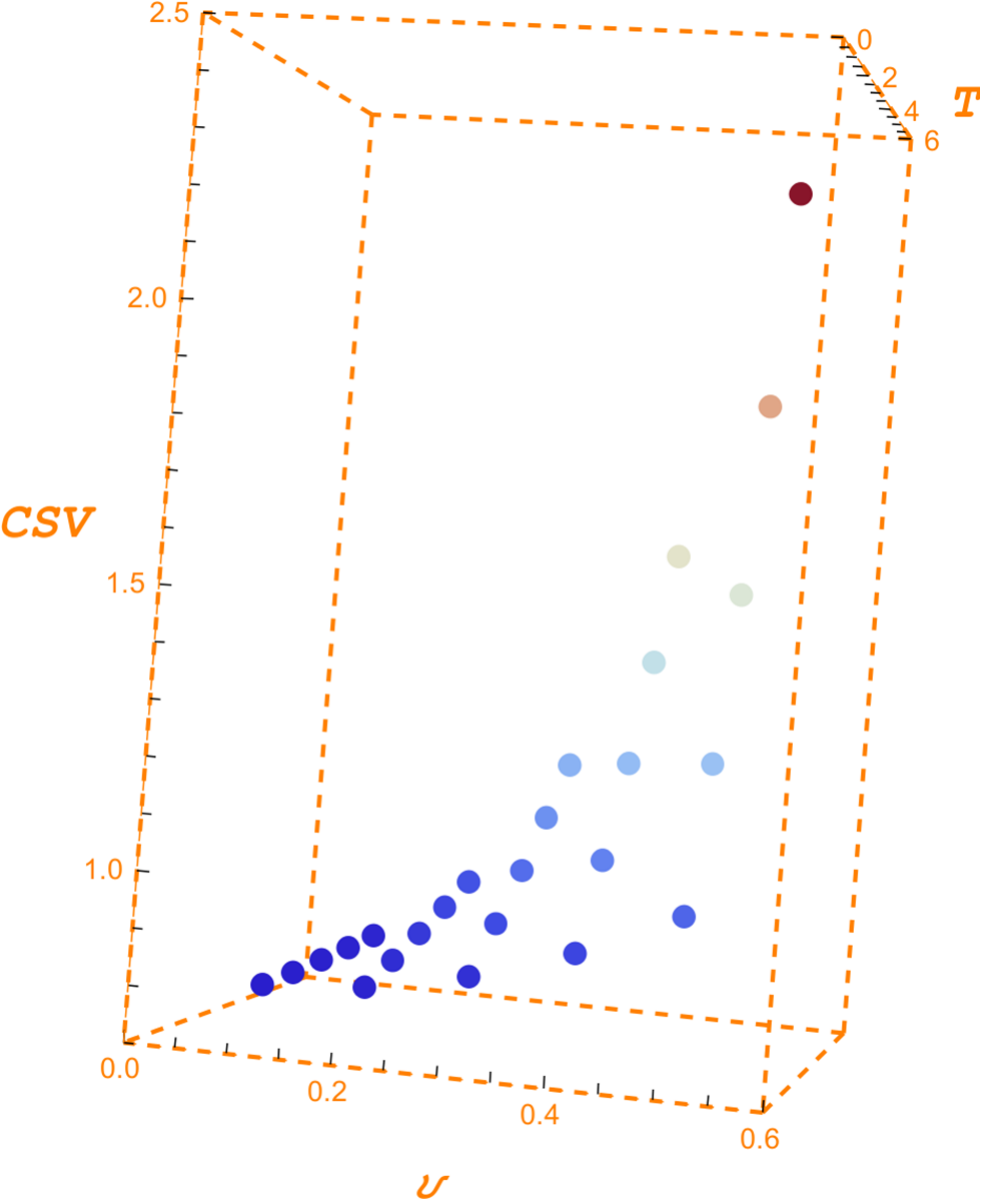
Cost Savings Ratio. Fig 8 depicts the shows the evolution of the Cost Savings Ratio (CSV) according to differential volatility and time for an Opportunity Savings Ratio equal to 0.20. Horizons are 1–5 years and differential volatilities: 0.1, 0.2, 0.3, 0,4 and 0.5. We can observe how the CSV increases as volatility and horizon increase.

Tables 6 and 7 display a sensitivity analysis for the OSR and the CSV. Their values are plotted, respectively, in Fig 7 and Fig 8. Table 6 shows, for instance, that for a differential volatility of 20%, a 5 year horizon and a CSV equal to 1, it is worth spending the 17.69% of the expected savings from a new substitution technique in order to develop it. This percentage grows to 31% if the CSV goes down to 75%. Nevertheless, if the CSV raises to 125%, then the maximum percentage to be spent in the new opportunity decreases until 9.78%. A CSV greater than 1 means that, at the present moment, the expected costs are greater than the expected savings. Nevertheless, the option, i.e. the opportunity, has a positive value because the sign of this difference can be reversed at the moment at which the change must be decided. In short, in this case we have an exchange out-of-the-money option.

**Table 6:** Opportunity Savings Ratio.

*a*	0.25	0.5	0.75	1	1.25
T	*υ* = 0.10				
3	0.7500	0.5000	0.2530	0.0690	0.0090
5	0.7500	0.5000	0.2590	0.0890	0.0208
10	0.7500	0.5011	0.2768	0.1256	0.0497
15	0.7500	0.5040	0.2941	0.1535	0.0752
20	0.7501	0.5081	0.3100	0.1769	0.0978
25	0.7502	0.5131	0.3248	0.1974	0.1183
50	0.7532	0.5416	0.3856	0.2763	0.2005
	*υ* = 0.15				
3	0.7500	0.5002	0.2651	0.1034	0.0313
5	0.7500	0.5017	0.2812	0.1332	0.0564
10	0.7501	0.5105	0.3175	0.1875	0.1083
15	0.7508	0.5227	0.3481	0.2285	0.1502
20	0.7523	0.5358	0.3747	0.2627	0.1860
25	0.7545	0.5488	0.3984	0.2923	0.2176
50	0.7716	0.6073	0.4903	0.4041	0.3387
	*υ* = 0.20				
3	0.7500	0.5021	0.2839	0.1375	0.0603
5	0.7501	0.5081	0.3100	0.1769	0.0978
10	0.7515	0.5299	0.3633	0.2482	0.1707
15	0.7554	0.5530	0.4057	0.3015	0.2274
20	0.7609	0.5750	0.4415	0.3453	0.2746
25	0.7673	0.5953	0.4726	0.3829	0.3156
50	0.8023	0.6773	0.5883	0.5205	0.4667
	*υ* = 0.30				
3	0.7503	0.5152	0.3304	0.2050	0.1260
5	0.7523	0.5358	0.3747	0.2627	0.1860
10	0.7640	0.5853	0.4575	0.3647	0.2958
15	0.7795	0.6274	0.5192	0.4387	0.3766
20	0.7954	0.6631	0.5689	0.4977	0.4415
25	0.8107	0.6939	0.6106	0.5467	0.4957
50	0.8713	0.8016	0.7511	0.7112	0.6780
	*υ* = 0.50				
3	0.7594	0.5696	0.4330	0.3350	0.2635
5	0.7760	0.6186	0.5068	0.4238	0.3603
10	0.8187	0.7093	0.6311	0.5708	0.5223
15	0.8538	0.7722	0.7133	0.6671	0.6291
20	0.8816	0.8186	0.7728	0.7364	0.7062
25	0.9036	0.8540	0.8177	0.7887	0.7643
50	0.9635	0.9462	0.9334	0.9229	0.9139

Table 6 shows the values of the Opportunity Savings Ratio per the indicated values of differential volatility (*υ*), Cost Savings Ratio (CSV) and horizon (T).

**Table 7:** Cost Savings Ratio.

*υ*	0.1	0.2	0.3	0.4	0.5
T	OSR = 0.10				
1	0.9086	0.9594	1.0469	1.1695	1.3318
2	0.9247	1.0294	1.2050	1.4637	1.8342
3	0.9419	1.0993	1.3689	1.7905	2.4432
4	0.9594	1.1695	1.5419	2.1601	3.1910
5	0.9769	1.2408	1.7263	2.5807	4.1115
	OSR = 0.20				
1	0.8004	0.8142	0.8514	0.9110	0.9936
2	0.8033	0.8434	0.9289	1.0616	1.2520
3	0.8082	0.8763	1.0127	1.2297	1.5582
4	0.8142	0.9110	1.1020	1.4170	1.9220
5	0.8209	0.9470	1.1968	1.6260	2.3548
	OSR = 0.30				
1	0.7000	0.7026	0.7170	0.7462	0.7908
2	0.7002	0.7135	0.7556	0.8288	0.9375
3	0.7011	0.7287	0.8014	0.9247	1.1128
4	0.7026	0.7462	0.8517	1.0321	1.3187
5	0.7047	0.7653	0.9059	1.1514	1.5593
	OSR = 0.40				
1	0.6000	0.6003	0.6045	0.6172	0.6399
2	0.6000	0.6032	0.6217	0.6607	0.7228
3	0.6001	0.6092	0.6457	0.7154	0.8255
4	0.6003	0.6172	0.6736	0.7781	0.9463
5	0.6007	0.6266	0.7045	0.8482	1.0866
	OSR = 0.50				
1	0.5000	0.5000	0.5008	0.5050	0.5151
2	0.5000	0.5005	0.5069	0.5254	0.5588
3	0.5000	0.5021	0.5179	0.5547	0.6170
4	0.5000	0.5050	0.5321	0.5899	0.6867
5	0.5000	0.5090	0.5487	0.6300	0.7679

Table 7 shows the values of the Cost/Savings ratio per the indicated values of differential volatility (*υ*), horizon (T) and Opportunity Savings Ratio.

Table 7, in turn, shows that for a 20% differential volatility and 3 year horizon we need a CSV of 87.63% to justify expending the 20% of the SPV in the creation of the new technique. With a differential volatility of 30%, even with a present value of expected costs greater than the present value of the expected savings (101.27% CSV) the 20% expense is justified. It is again the case of an out-of-the-money option.

## Real Options Valuation and CSR: the Minimum Expectations Scenario

The scale-free sensitivity analysis developed in the previous sections is a first step about analyzing the CSR capacity to create value through the real option lenses. Nevertheless, when a corporation considers to undertake a specific course of action to be valued as a real option two further steps are required: choosing the appropriate valuation method, and estimating the parameters required by it. Favato and Print [24] perform a sensitivity analysis for valuation models and parameters estimation at the same time. They show that the real options value is more sensitive to the estimation of the parameters than to the election of the most appropriate valuation model, concluding that (p. 66) " So 80% of the time, the error in just one input of the model could have been ten times more relevant than the choice of the real option pricing model". Any course of action, unless it is deterministic, can be regarded as a set of random expectations. The estimation of the nature of their evolution, i.e. the random walk they follow, leads to choose the most appropriate valuation method. The estimation of the parameters in which the valuation method summarizes the random expectations is the next step. As known, the main difference with respect to parameters between financial and real options is the lack of market data for real options. Cobb and Charnes[25] compare the variables of financial and real options in the Black-Scholes framework summarizing, the main sources of uncertainty that real options analysis has to face. Copeland and Antikarov [21], chapters 8–10, make detailed proposals to value real options, and, thus, to estimate their parameters. These authors consider the subjective estimates provided by management when historical data are impossible to obtain. Technically, the Monte Carlo analysis combined with binomial trees is the most usual approach to estimate the parameters of real options as shown by Copeland and Antikarov [21], Cobb and Charnes [25], Herath and Park [26], and Godinho [27], among others. Uncertainty is even more relevant for CSR projects than for ordinary projects. Our contribution with respect to parameters estimation in this paper is not to propose a new method or to enlarge the existing ones. Instead, we develop the analysis of the minimum expectations scenario that a real option must fulfil in order to become a feasible investment project. Thus, we obtain the benchmark with respect to the objective and the managerial subjective estimates can be compared before making a decision. The more uncertain the parameters estimation is, the more relevant becomes the estimation of the minimum expectations scenario, because it formalizes the minimum sets of goals that managers must feel able to achieve it they decide to undertake the project. A basic aim of the minimum expectations scenario is to help managers to think in terms of real options even when uncertainty pervades the project and to estimate the parameters on basis of objective data becomes highly difficult. We divide our approach to the minimum expectations scenario into the estimation of its parameters and their analysis from the perspective of financial sustainability.

### Estimating the Parameters of the Minimum Expectations Scenario

Be a corporation that is considering to undertake a preliminary project that will make feasible a main project in the future. At this stage, the main project is unfeasible because its capital outlay is greater than its GPV. The preliminary project may make feasible the main project at a certain horizon. In this context, the preliminary project can be regarded as a call option on the main project. Expectations are uncertain and the corporation asks itself which are the minimum expectations that justify to undertake the preliminary project. The procedure to follow can be summarized in three steps:

1. Estimating the minimum value of the call that makes feasible the preliminary project.
2. Calculating the volatility embedded in this minimum value of the call, i.e. the breakeven volatility.
3. Estimating the expectations implied by this volatility through a binomial tree.

In this analysis we have to bear in mind that the role of the preliminary project is to improve the expectations of the main project in order to make it financially sustainable in a certain horizon. In order to justify to invest in the preliminary project the capital outlay necessary for undertaking it, the question that we must answer, is:

- Which is the set of minimum expectations that the preliminary project must create for the main project?

The minimum value of the call that makes financially sustainable the preliminary project is the capital outlay required by it. Then, its NPV equates zero. The amount to be invested in the main project in the future, i.e. at the call maturity, is the call strike price. The GPV of the main project at the current moment is the present value of the underlying asset. The risk free interest rate is supposed to be known. Having estimated these parameters, we can calculate the value of the volatility embedded in the option.

As a first approach, we can apply the Black and Scholes formula and calculate the volatility embedded in it by numerical methods. Nevertheless, for some assets it may be necessary to apply a different model based on a stochastic process that, for the case under analysis, better captures the evolution of its price as, for instance, a mean reverting process. Customized models could be very appropriate once their features have been identified. These limitations are nonetheless compatible with the essence of our proposition: first, identify the minimum volatility that makes the project feasible and, next, analyse the expectations that it embeds by means of a binomial tree.

The breakeven volatility encapsulates the less optimistic state of the world or, in other words, the minimum expectations scenario that justifies to undertake a project. This scenario can be made explicit by means of binomial trees, which have become the standardized way for the valuation and analysis of real options. Binomial trees, according to Copeland and Tuffano [28], p. 93, "are ideally suited to real option valuation" because the transparency of their models enables managers to control them and even to adapt them to specific situations. Their simplified mathematics enables managers to concentrate on the strategic courses of action analyzed through real options. As Chance [29], p. 38, points out the central quality of binomial trees is "its ability to illustrate the essential ideas behind option pricing theory with a minimum of mathematics and to value many complex options". In the same line, Favato and Print [24] underline the importance of releasing managers from the complex world of option valuation mathematics in order to help them to concentrate on real options thinking, i.e. on the strategic facet of real options analysis. As known, the original purpose of binomial trees, as developed by Cox, Ross and Rubinstein [30], was to simplify the Black and Scholes model. Since then, many techniques for applying binomial trees to different stochastic processes have been built up. Chance [29] presents a synthesis of them. Binomial trees have also been extended to trinomial and quadrinomial trees.

Adopting the technique of binomial trees and taking into account the relationship between volatility and the tree drivers (Cox, Ross and Rubinstein [30]), the possible values of the asset at the maturity goes from the lowest one *A*_0_ *d* ^*n*^ to the highest *A*_0_ *u* ^*n*^, with *A*_0_ in the centre:

$$\left\{A_{0}d^{T},A_{0}d^{T-1}u,\ldots ,A_{0}d^{\frac{T+1}{2}}u^{\frac{T-1}{2}},A_{0},A_{0}u^{\frac{T+1}{2}}d^{\frac{T-1}{2}},\ldots ,A_{0}u^{T-1}d,A_{0}u^{T}\right\}$$ (19)

where $u=e^{\sigma \sqrt{\Delta T}}$ and *d* = 1 / *u*.

In these expressions, *T* designates the number of steps in the tree, *u* the upside factor, *d* the downside factor, while Δ*T* stands for the length of each step

This is the minimum set of expectations that justifies to undertake the preliminary project, because the value of the call option that stems from them equates the capital outlay necessary for this preliminary project. Therefore, it makes the project feasible from the point of view of the present value. From the point of view of final value, the condition that makes the project successful is a gross final value (GFV) equal or greater than the addition of the exercise price and the final value of the preliminary investment (PI):

$$GFV\geq K+PIe^{rT}$$ (20)

In terms of upside and downside factors, the GFV can be expressed as:

$$GFV=A_{0}u^{w}d^{{}^{T-w}}=A_{0}u^{2w-T}$$ (21)

where *w* denotes the number of upside jumps.

From a risk averse perspective the decision maker may opt for capitalizing the preliminary investment at a required rate of return that includes a risk premium. Then, in (20)the risk free rate must be substituted by the required rate and the minimum number of upside jumps must be recalculated accordingly. Below we develop the corresponding details.

Since options value increases with volatility and the set of expectations displayed by this tree corresponds to the breakeven or minimum volatility, it represents the minimum scenario that justifies to undertake the preliminary project in which the call option consists of. In the next section, we proceed to analyse its features and to relate it with real options thinking.

### Analysing the Minimum Expectations Scenario

We develop the analysis of the minimum expectations scenario in three steps: a) measuring risk from the proportion of upside vs. downside jumps in the binomial tree, b) comparing risk neutral, objective and subjective probabilities, and c) summarizing the results of the previous steps in a rule for comparing scenarios and making decisions.

#### Risk

We define the break even node as the last period node that leads to the minimum *GFV* that fulfils (21). The number of upside jumps of this node is:

$$w*\equiv Min\;w\Rightarrow A_{0}u^{2w-T}\geq K+PIe^{r\;\text{T}}$$ (22)

Hence *w* ^*^ is the result of rounding up *w* in:

$$w=\frac{1}{2}\left(\frac{\text{ln}\left[\left(K+PIe^{r\;\text{T}}\right)/A_{0}\right]}{\text{ln}\left(u\right)}+T\right)$$ (23)

The ratio between the minimum number of upside jumps to avoid a negative GFV and the maximum possible number of upside jumps (*w* ^*^ / T) can be regarded as a risk indicator for the course of action, complementary to volatility. The use of this ratio requires to define the jumps for the time measure, month for instance, that managers regard as most meaningful.

In order to obtain the breakeven node that corresponds to a risk averse required rate of return, we substitute in (23) the risk free rate by the risk averse one, and, next, we round the result according to (22).

#### The Probabilities of the Minimum Expectations Scenario

Once we have estimated the minimum volatility and its corresponding upside and downside factors, we can obtain the probabilities associated to the binomial tree for risk neutrality and risk aversion. A core point of risk neutral valuation is the is the relationship among the up and down factors and the risk free interest rate with the risk free interest rate Cox, Ross, Rubinstein [30] write as:

$$p_{u}=\frac{1+r-d}{u-d}$$ (24)

Risk averse probabilities are obtained (Brooks and Chance [31]) by substituting in (24) the risk free interest rate by the risk adjusted required rate of return (*R*):

$$p_{u}^{*}=\frac{1+R-d}{u-d}$$ (25)

that, after taking into account that *d* is the inverse of *u*, and *u* is equal to $e^{\sigma \sqrt{\Delta T}}$, the upside risk averse probability can be written as:

$$p_{u}^{*}=\frac{u\left(1+R\right)-1}{u^{2}-1}=\frac{e^{\sigma \sqrt{\Delta T}}\left(1+R\right)-1}{e^{2\sigma \sqrt{\Delta T}}-1}$$ (26)

About this relationships, Copeland and Antikarov [21] argue that the option values, probabilities, and the discounting rate constitute a triad that enables us to obtain the value of each one of them from the remaining two. The basis of this assertion is that option valuation is independent from the risk attitude when the replying portfolio holds. Copeland and Tufano [28] underline the relevance of relating probabilities to the decision making processes that stem from binomial trees, because managers can associate changes in value directly with probabilities. A relevant difference in the applicability of risk averse valuation to primitive assets, as the underlying project, and option is the set of values taken by the required rate of return: while for primitive assets, as the underlying asset, it is constant, in option valuations it changes from node to note, because options' risk depends on the evolution of the underlying asset (Copeland and Antikarov [21]). Therefore, from (25) we can obtain the up and down probabilities for the main project that are part of the set of expectations that justifies to undertake the preliminary project.

In this context, $p_{u}^{*}$ can be regarded as the required upside probability for a manager or an investor who requires an expected rate of return equal to *R* for the given volatility. Thus, $p_{u}^{*}$ expresses the required expectation by this investor. To this extent, $p_{u}^{*}$ is a subjective probability. The project is accepted if the objective probability $\left({\hat{p}}_{u}\right)$, i.e. the probability that stems from the analysis of the market forecasts and corporate skills to undertake the project, is equal or greater than the required probability:

$${\hat{p}}_{u}\geq p_{u}^{*}$$ (27)

The minimum expectation probabilities are not embedded in the main project itself. Instead, they are the minimum probabilities that the preliminary project must be able to create for the main project in order to make itself feasible.

#### Making Decisions Comparing Scenarios

The minimum expectations scenario can be taken as a basis for making decisions. In fact, it is much more explicit that a single required rate of return, but at the same time it is compact enough to put aside minor details. It can be summarized at two levels. First from the risk neutral perspective:

{u,d,p,w,r} (28)

and, second, from the risk averse perspective:

$$\left\{u,d,p^{*},{\text{w}}^{*},R_{A}\right\}$$ (29)

The probabilities of the minimum expectations scenario should be compared with the objective probabilities what may stem from an accurate forecast and to the subjective probabilities that stem from managerial experience. As known, the theoretical foundations of subjective or personal probabilities are mainly due to Savage [32]. Copeland and Antikarov [21], pp. 259–264, develop a framework to identify subjective probabilities, and to infer the volatilities and stochastic processes implied in the subjective estimates provided by managers.

Subjective probabilities can be considered in real options because, in most cases, the owner of a real option is an active subject, instead as a the passive subject that is the owner of a financial option. The value of financial options depends on the evolution of an underlying asset traded in the market. Thus, the financial option's owner is passive with respect to this evolution. Nevertheless, the evolution of the value of a real option is an interaction between the management of the project to which it is associated and the evolution of the market. As Mills, Weinstein and Favato [33], p. 55, point out, real options have an active management feature that "*is* a response to a scenario and its outcomes". For instance, the evolution of the value of a CSR option that consists of a research to create a cleaner production technique depends at the same time on the success of this research and on the changes in the industry and the economy.

To sum up, from the point of view subjective probabilities, the question that a manager must answer in real options analysis is:

- Can we reach at least *w* ^*^ upside jumps with probability *p* ^*^?

This means to capture the required rate of return to the preliminary project with the minimum nonnegative NTV.

## Empirical Illustration

Be a car maker that now has not any electrical model in its range of products. Currently the corporation has been criticized for producing cars that only use fossil energies. This has been the reason for not being included in a CSR index. Furthermore, its shares have been excluded from the portfolios of several responsible investment funds. The marketing department holds that the corporation cannot survive in the medium future without a reasonable share in the electric car market. Since its market share in this subsector is nil now, the launching a standard electric car will have a negative NPV unless it incorporates a relevant novelty that makes it a substantially better model. With this aim, the research department of this corporation is studying a new electric engine that, if successful, will enable its users to make trips substantially longer than the existing models without recharging the batteries. The greater the autonomy of the new engine, the greater the GPV of the new model at the end of the research. According to the classification presented in the Second Section of this paper, the research of the new engine is a new, internal, preliminary and pure corporate project.

The estimations of the financial department show that launching a new electric car requires a capital outlay of 1000$, while its estimated GPV is 750$. Thus, it would have a negative NPV of 250$ and a GPV Index of 0.75. The autonomy of the new engine depends on the results of the research. The research would last 3 years and the present value of its expenses is 250. The risk free interest rate is 2%. At this initial stage, the managers do not have an accurate estimation of the project volatility. They provisionally decide to assume a volatility of 50%, although, according their past experience on research projects, it could be greater.

The managers submit the project to a preliminary analysis on basis of the OPR, the NPR, the minimum GPV Index and the minimum horizon. From Table 1, the OPR for this case is 0.1966, while, from Table 2, the corresponding NPR is 0.2621. Table 3, in turn, shows that to reach an OPR of 0.25, the dataset we have would need a GPV index of 0.84. The horizon for reaching the same value of the OPR with the GPV index that we have, i.e. 0.75, is 4.25 years. All these figures put into question the financial sustainability of the preliminary project. According to the amount to be invested in it, the minimum OPR should be 0.25 (250/1000) and the minimum NPR 0.33 (2250/750). Furthermore, to reach an OPR of 0.25 we would need a GPV index of 0.84 instead of the 0.75 that we provisionally have. Finally, the horizon should be 4.25 years instead of 3.

After this preliminary analysis, the managers wonder about the financial sustainability of the research project. For this reason, they revise the dataset. They regard all data as reliable except volatility, and proceed to interpret it in terms of binomial trees. The upside factor for a yearly volatility of 0.50 is 1.6486 (*e* ^0.50^), while its monthly equivalent is 1.1553 $\left(e^{0.50\sqrt{1/12}}\right)$. Their corresponding downside factors are 0.6065 and 0.8656. Managers think that their action can improve these factors. As a first step, they decide to study the minimum expectations scenario.

The minimum value of the call that makes the CSR project financially sustainable is 250$. Thus, the Preliminary Investment/Strike Price Ratio (PIK ratio) is 0.25. From Table 7, they obtain that the corresponding minimum volatility is 0.6062. The financial manager decides to display the corresponding binomial tree in monthly periods, i.e. 36 months. The upside factor, calculated as $e^{0.6062\sqrt{1/12}}$ is 1.8334 while the downside factor is 0.5454. Their monthly equivalents are 1.1912 and 0.8394. The upside and downside risk neutral probabilities calculated from (24) respectively are 0.4622 and 0.5389. The minimum GFV to make the project successful is:

$$250e^{0.02*3}+1000=1265.50$$

**Fig 9:**
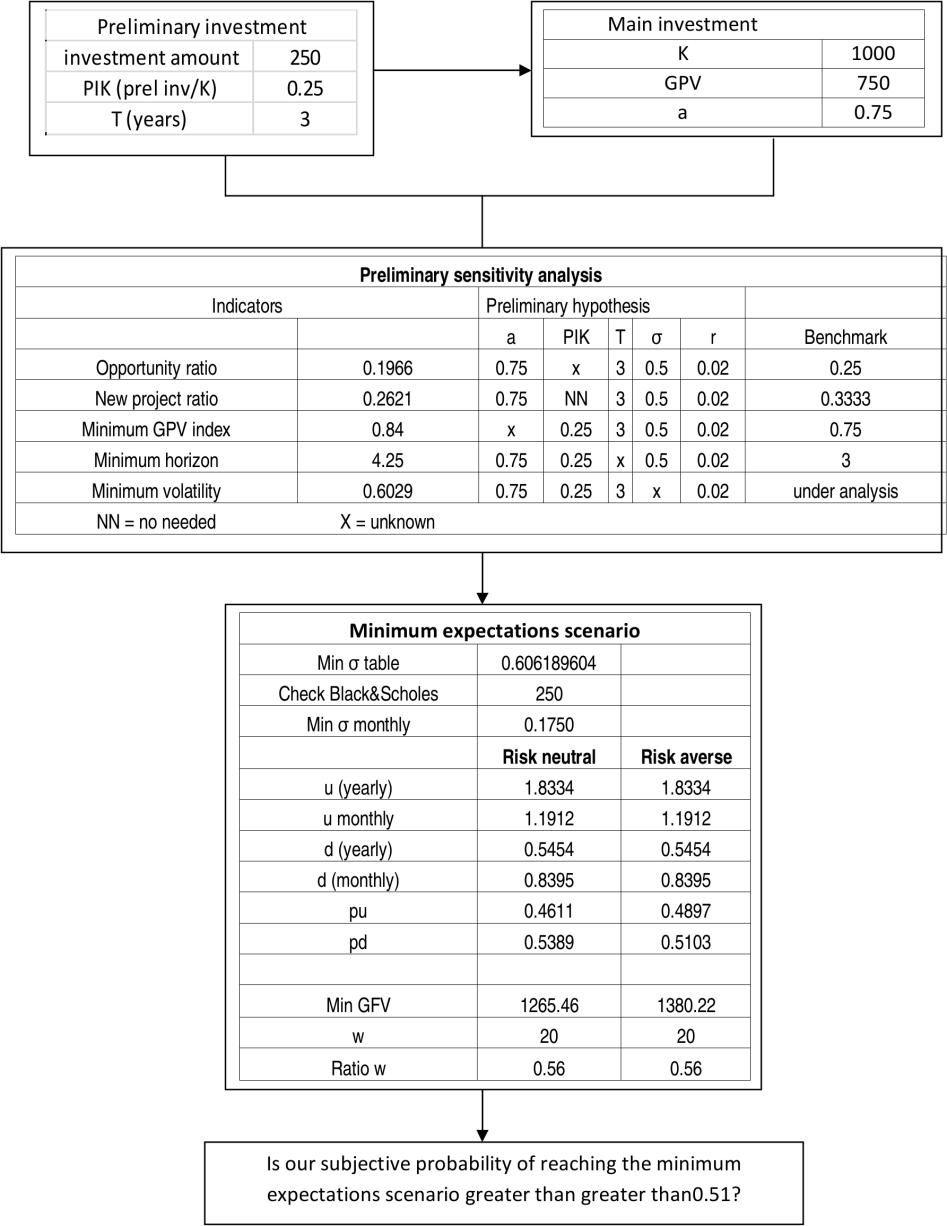
Synthesis of the empirical illustration.

**Fig 10:**
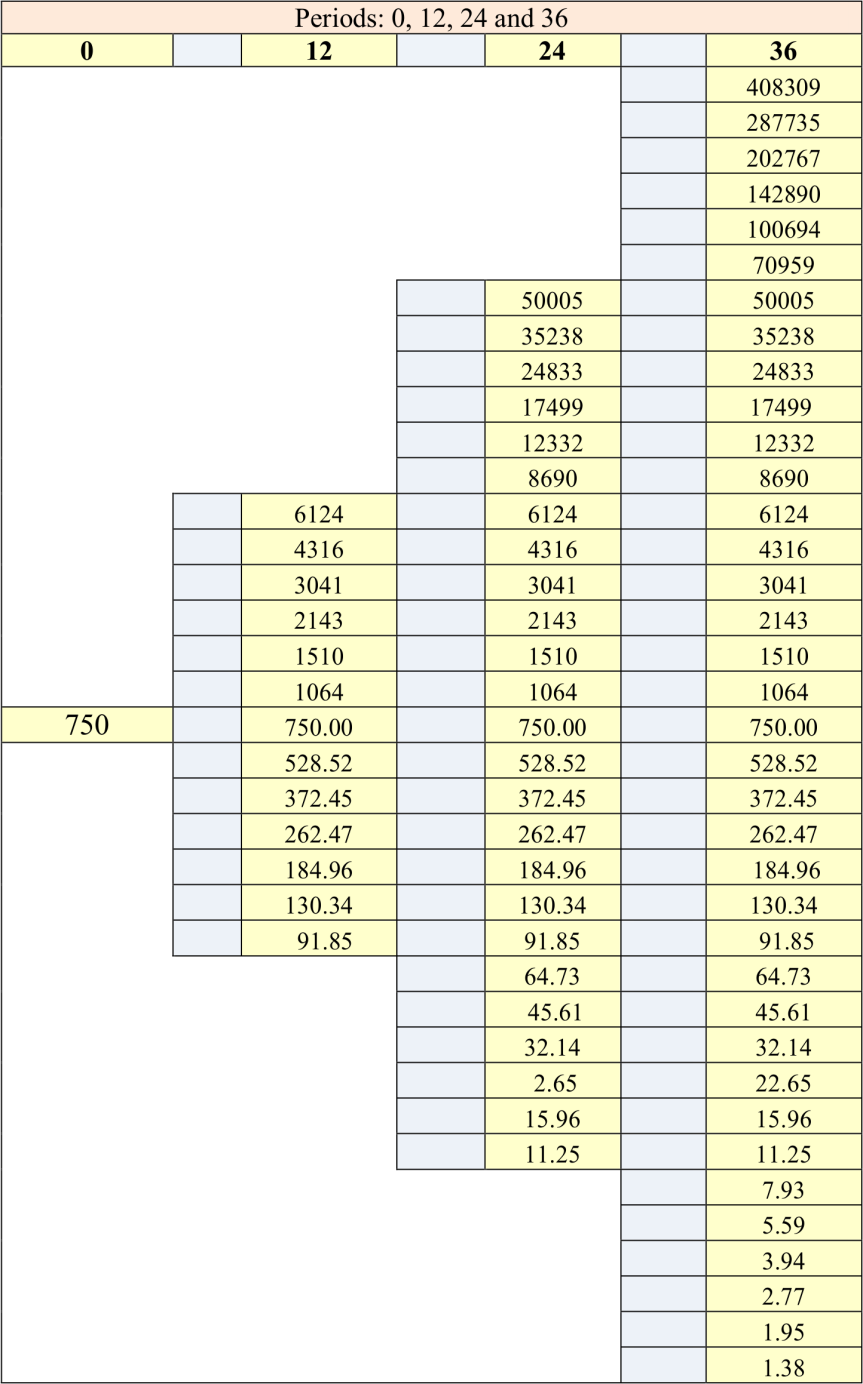
Asset tree.

**Fig 11:**
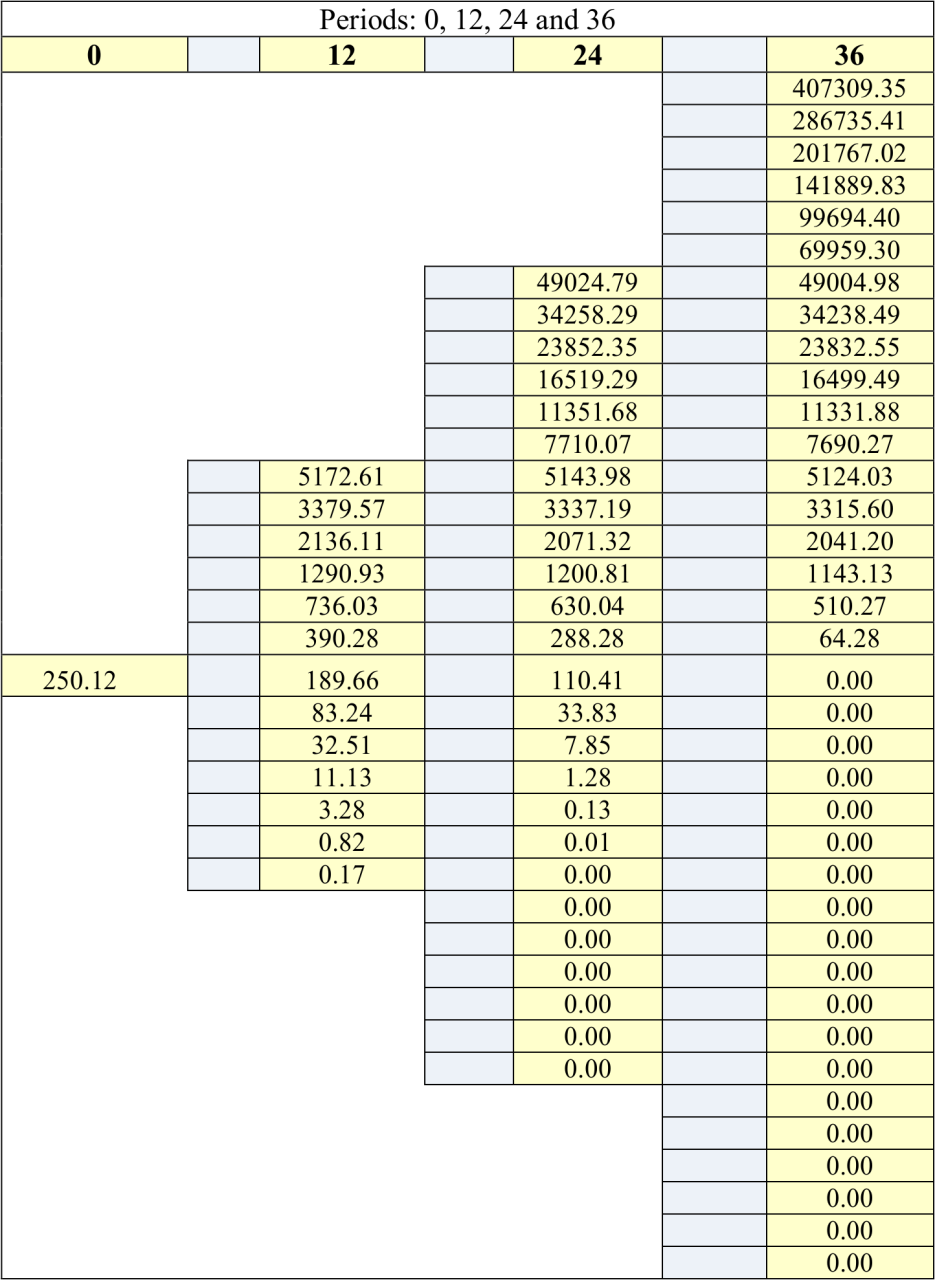
Call option tree.

The minimum number of upside jumps, calculated from (22) is 20, which means that the *w* ratio is 0.5556 (20/36). Managers know that corporate shareholders are risk averse. They consider that the appropriate required rate of return for the new engine is 15%. On this basis, they calculate the corresponding upside and downside probabilities applying (25) and obtain 0.4897 and 0.5103. Let us underline that the fact that the risk averse probability is greater than the risk neutral one, does not mean that a risk averse manager is more optimistic than a risk neutral manager. Conversely, it means that the risk averse manager requires a greater upside probability in order to accept the project. Fig 9 displays the synthesis of these calculations, while Fig 10 and Fig 11 display the evolutions of the asset and the call option.

From the analysis of the minimum expectations scenario, the managers become aware of the fact that, as it stems from their required rate of return, in order to be feasible, the preliminary project needs at least a monthly upside factor of 1.1912 with a minimum of 20 upside jumps. The probability of an upside jump at each month should be 0.4897. The managers feel able to reach these goals. Their subjective probability is over 0.50 for all of them. Then, they proceed to study the actual scenario and begin to design the courses of action that they regard as the most appropriate to make successful the research on the new engine. They will make their final decision after this analysis.

## Results and Discussion

The central results of this paper are the real option ratios and the scenario of minimum expectations for the analysis of CSR decisions. Real option ratios as value creation indicators present two generic advantages: They are scale free and they are related to the central variables of value creation through the rigorous quantitative framework of option theory. The OPR and the NPR express, respectively, the value of the opportunity, i.e. the option, on basis of the future capital outlay and the current value of the project to be developed in the future if the opportunity becomes successful. In any initial state we should expect a NPR greater than the OPR, which means that the main project shows now a negative NPV, because if (*c* / *GPV*) < (*c* / *K*), then *NPV* = *GPV* – *K* < 0. The feasibility of the preliminary project requires, in turn, a positive NPV that, in this case, means that the value of the call option in which it consists of is greater than the capital outlay necessary for undertaking it. Volatility is the key to understand the opposite signs of both NPVs. When the main project shows a negative NPV the part of its volatility that cannot be eliminated through diversification makes its required rate of return greater than its expected rate of return. Nevertheless the same volatility that makes the future project unfeasible now makes feasible the preliminary project because it consists of an option. In fact, these effects mirror the twofold and opposite impact of volatility on the values of basic assets, as shares and bonds, and options. The greater the volatility the lower the values of basic assets and, at the same time, the greater the values of options on them. The role of volatility is enlightened by the calculation of its breakeven value. Once we know the amount to be invested in a preliminary project, we can obtain the volatility that justifies this investment by making the call option in which the preliminary investment consists of equal to it.

The extension of the options based ratios to substitution projects has lead us to switch from classical option to exchange options where the reference ratio becomes the quotient between the value of the opportunity with the present value of the estimated savings that would be obtained through the new technique, i.e. the OSR. The decision about starting the preliminary substitution project is to be made by comparing the value of the exchange option with the capital outlay necessary for developing the preliminary project.

**Fig 12:**
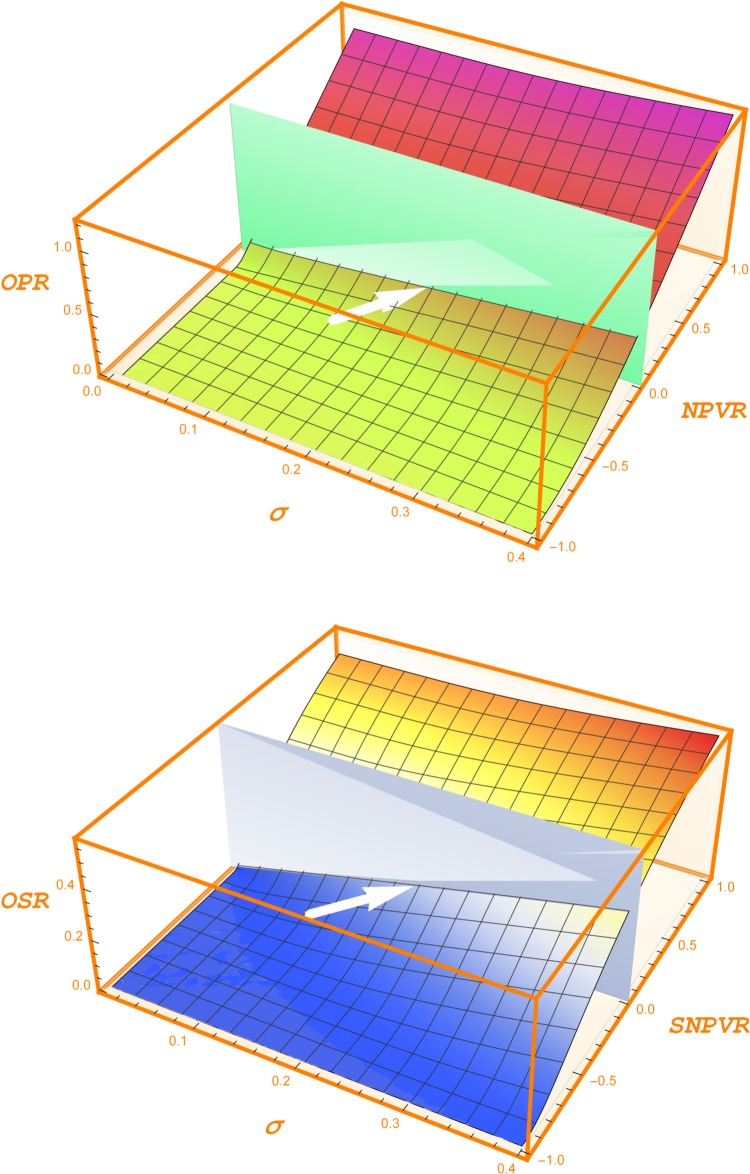
Value creation. The upper plot summarizes the meaning of the Opportunity Ratio. In it we make use of the quotient between the net present value and the future capital outlay, i.e. the NPV ratio (*NPVR*). It equates the GPV index minus 1. If a CSR decision arouses the opportunity to start a new project in about 5 years with a volatility of 20% and a nil net present value ratio now, it is worth spending 22% of the project’s capital outlay to be invested in the future to create this opportunity (being the risk-free interest rate 2%). The white arrow signals this point. The vertical plane separates the out-of-the money and the in-of-the money option values. The bottom plot summarizes the meaning of the Opportunity Savings Ratio. In it we use the quotient between the Savings Net Present Value (Savings Present Value minus New Technique Costs) and the New Technique Costs. It equates the inverse of the Cost Savings Ratio minus 1. We call this quotient Savings Net Present Value Ratio (*SNPVR*). For a substitution project with nil SPVR now, 20% differential volatility and 5 year horizon, it is worth expending the 17% of the expected savings to create the new technique. This point is also signalled by the white arrow of this plot.

Real option ratios cannot be regarded as static results. Not only the values of their independent variables are subjected to changes, but also some of them are very difficult to estimate. For this reason, the consideration of different scenarios becomes compulsory. In fact, before making a decision, a sensitivity analysis is highly recommendable. A remarkable advantage of option ratios is that they enable us to systematize the sensitivity analysis by constructing tables that, in addition, can be turned into figures. Certainly, the sensitivity of option ratios to their independent variables can be approached as well by means of differential calculus. Nevertheless, the numerical nature of sensitivity analysis creates a framework that arouses intuition. To this extent, we can compare their intuitive value to the information contained in the tables of standardized statistical distributions: they contribute to capturing the information embedded in the mathematical functions that lay behind them. Looking with this perspective to the sensitivity analysis we have performed in the previous section, we can conclude that investing in building up future opportunities creates value at the present moment under quite reasonable assumptions. A long run horizon and high volatility are the best allies to create value. The challenge that decision makers face in order to make CSR expenses financially sustainable is to identify the investment opportunities associated to them. To bear in mind the value creation capacity that new opportunities have, we can retain the following results that encapsulate the essence of the sensitivity analysis we have performed. If a CSR decision arouses the opportunity to start a new project in about 5 years with a volatility of 20% and a nil NPV today, it is worth spending 22% of the project’s future capital outlay to create this opportunity, being the risk free interest rate 2%. For a substitution project with 20% differential volatility and 5 year horizon, it is worth expending 17% of the expected savings to create the new technique. These results show that, for volatility, horizon and interest rate values usual in financial markets, value creation through new opportunities is far away from being difficult. Fig 12 depicts them. The difficulty lies in identifying the opportunities, but the quantitative framework is essential in order to capture the value creation features of each opportunity.

**Fig 13:**
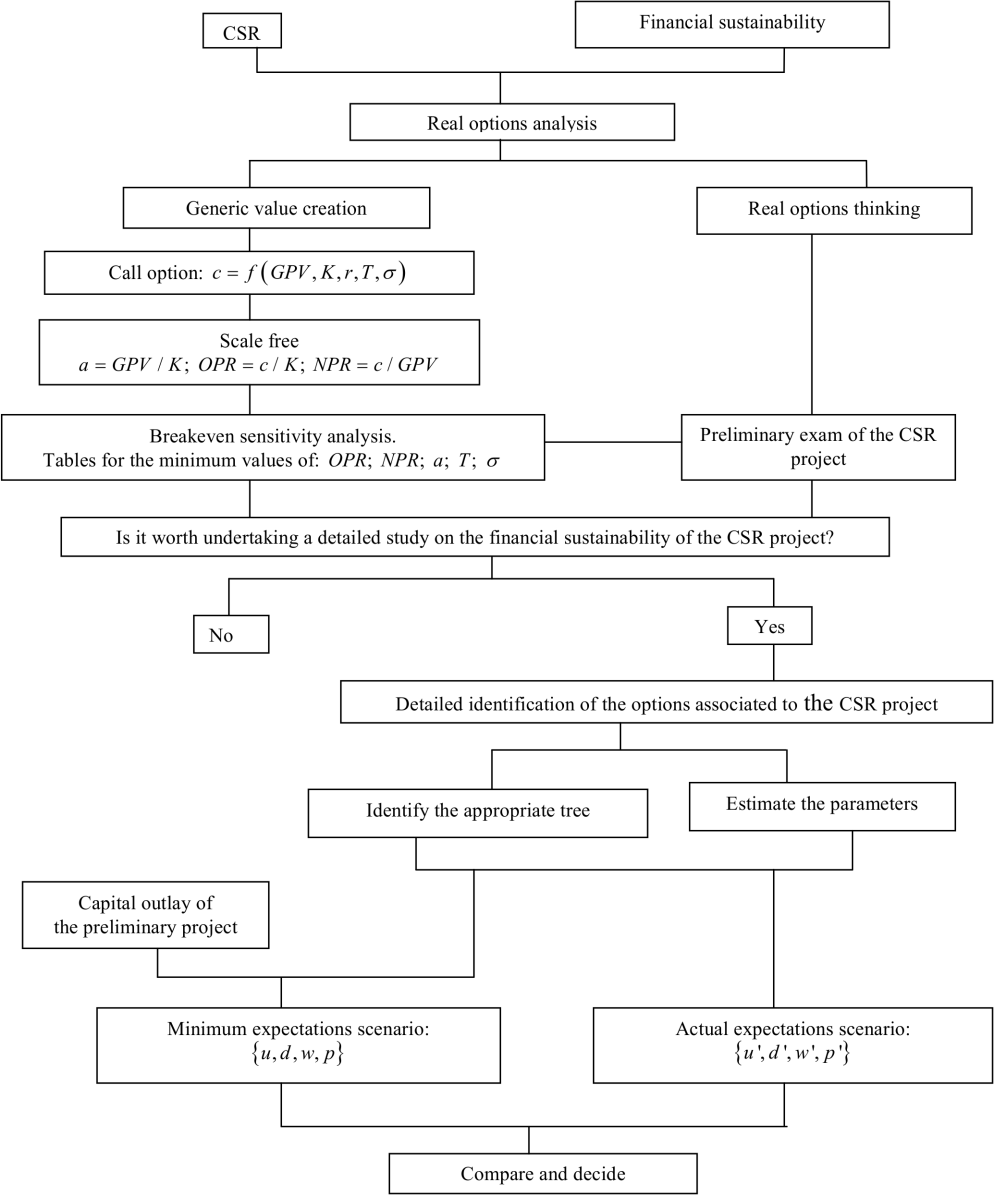
Conceptual map of the analysis of the financial sustainability of CSR projects.

Among the different scenarios that is worth considering, the minimum expectations scenario plays a central role. It enables the analyst to know the minimum volatility, the minimum upside factor and its symmetrical downside factor, the minimum number of upside jumps necessary to obtain a nonnegative NTV, the risk neutral probabilities and the risk averse probabilities associated to the project's required rate of return. The minimum expectations scenario can be taken as the benchmark to which the estimated actual expectations scenario is to be compared. In particular, managers answer if they regard themselves able of overcoming the minimum expectations scenario with a probability greater than the risk averse one that fits with the project's required rate of return. Fig 13 synthesizes the financial sustainability analysis for CSR projects that we have presented in this paper.

Beyond the opportunities that we have characterized as preliminary projects, other kinds of real options can be found embedded in full projects, internal of external. They simply consists of the well known catalogue of real options: Option to expand, option to abandon, option to delay among others. Embedded options have a crucial difference with preliminary projects. Any preliminary project requires a capital outlay to be undertaken, but embedded options do not require an additional capital outlay because they are part of the full project or, in other words, they have been created by it.

Although real options can be confirmed as the central tool for CSR thinking, they cannot be applied to all CSR projects. The classification of CSR projects that we have developed in Section 2 enlightens this issue. In fact, this classification can be regarded as a tool to decide the most adequate approach for each kind of projects, because the knowledge of the nature of the problem logically precedes the election of the technique to solve it.

The analysis we have developed and the results we have obtained have, of course, limitations. Mainly, it cannot be constrained to classical and exchange options. Instead, the wide array of exotic options can be used in specific scenarios. A case in point are compound options, i.e. options that have another option as their underlying asset, that are often used in real option analysis because they serve to capture second order opportunities embedded in other opportunities. The appropriate trees for these cases are, of course, more complex, which can be extended to the estimation of the minimum expectations scenario

## Conclusions

A necessary condition for the financial sustainability of CSR projects is value creation. Nevertheless, the way through which CSR creates value is often indirect because it usually comes from the new opportunities for corporations that CSR arouses. For this reason, real options can be regarded as an essential tool to identify, at both the qualitative and quantitative level, the value creation capacity that stems from CSR initiatives. Once this premise has been accepted, the decision analysis on CSR must incorporate real options. A particularly relevant fact for CSR projects, comes from the out-of-the-money real options. The value of the real options in these cases expresses the amount that is worth investing now in order to create the opportunity to undertake a new project in the future that would have a negative NPV if started now, but that may have this negative NPV turned into a positive one at the moment of its inception.

The contribution of real options to decision analysis does not lie only on real options capacity to unveil hidden opportunities, but also on their capacity to interact variables in the precise framework of option valuation formulae. To take full profit from option theory to show the consequences of the mutual impact of the variables involved in option value, we need to switch from valuation in monetary units to option ratios. Then, we can perform sensitivity analysis without being subjected to the size impact on option value, because size creates a barrier that prevents the generalization of sensitivity analysis. In effect, the theoretical relationships of option valuation formulae are normalized in the tables that generalize options sensitivity analysis through ratios, which, by their own nature, are scale-free. They can be used as value creation indicators that enlighten strategic analysis before going into the capital budgeting level. But, if size is a barrier, the complexity of mathematics used in continuous time options' valuation is a barrier as well for the analysis of the implications of real options. Through real options analysis we do not only aim to obtain a single numerical figure, the option value, but to depict the scenario that leads to it. Binomial trees are the right tool to achieve this goal. Their application to the minimum expectations scenario shows the detailed features of the benchmark that managers must consider in making the decision of whether the preliminary project is worth undertaking.

Nevertheless, often the application of real option analysis to CSR needs to identify the complex relationships involved in CSR projects, because neither all CSR projects can be approached from the option theory perspective, nor the same kind of options are appropriate for the analysis of any CSR project that presents option features. The classification of CSR projects developed in the Second Section is aimed at smoothing the difficulties of dealing with the complex CSR setting. In this paper, this classification has enabled us to approach new projects by means of classical options, but substitution projects through exchange options; apart from, in the new projects setting, to distinguish between the projects that are options in themselves, i.e. preliminary projects, and projects that have embedded options in them.

To sum up, real option analysis proves it is a powerful tool to analyze CSR decisions, but a systematic framework for this analysis is needed. This paper has made an attempt in this direction through the classification of CSR projects, through the sensitivity analysis of real option ratios that enlightens the interactions of their variables from the value creation perspective, i.e. from the financial sustainability viewpoint, and, finally, through the minimum expectations scenario that can be taken as the benchmark for any preliminary project.

## Supporting Information

S1 Fig Mathematica code for Fig 2. (PDF)

S2 Fig Mathematica code for Fig 3. (PDF)

S3 Fig Mathematica code for Fig 4. (PDF)

S4 Fig Mathematica code for Fig 5. (PDF)

S5 Fig Mathematica code for Fig 6. (PDF)

S6 Fig Mathematica code for Fig 7. (PDF)

S7 Fig Mathematica code for Fig 8. (PDF)

S8 Fig Excel file for Figs 9, 10 and 11. (XLSX)

S9 Fig Mathematica code for Fig 12. (PDF)

S1 Table Mathematica code for Table 1. (PDF)

S2 Table Mathematica code for Table 2. (PDF)

S3 Table Mathematica code for Table 3. (PDF)

S4 Table Mathematica code for Table 4. (PDF)

S5 Table Mathematica code for Table 5. (PDF)

S6 Table Mathematica code for Table 6. (PDF)

S7 Table Mathematica code for Table 7. (PDF)
