# Supplementary material for: Corporate Social Responsibility: A Real Options Approach to the Challenge of Financial Sustainability
Source: PLoS One. 2015 May 4;10(5):e0125972. doi: 10.1371/journal.pone.0125972 (PMC4418608; doi:10.1371/journal.pone.0125972)

S1 Fig.: *Mathematica* code for Fig.2

```
ndist = NormalDistribution[0, 1]
```

NormalDistribution[0, 1]

```
Clear[K, A, σ, v, u, T, r, a, c]
```

$$d1 = \frac{Log[a] + \left(r + \frac{\sigma^2}{2}\right) * T}{\sigma * \sqrt{T}}$$

$$\frac{T\left(r + \frac{\sigma^2}{2}\right) + Log[a]}{\sqrt{T}\ \sigma}$$

$$d2 = d1 - \sigma * \sqrt{T}$$

$$-\sqrt{T}\ \sigma + \frac{T\left(r + \frac{\sigma^2}{2}\right) + Log[a]}{\sqrt{T}\ \sigma}$$

```
OPR = a * CDF[ndist, d1] - Exp[-r * T] * CDF[ndist, d2]
```

$$\frac{1}{2} a\,Erfc\left[-\frac{T\left(r + \frac{\sigma^2}{2}\right) + Log[a]}{\sqrt{2}\ \sqrt{T}\ \sigma}\right] - \frac{1}{2} e^{-r\,T}\,Erfc\left[\frac{\sqrt{T}\ \sigma - \frac{T\left(r+\frac{\sigma^2}{2}\right)+Log[a]}{\sqrt{T}\ \sigma}}{\sqrt{2}}\right]$$

```
r = 0.02
```

0.02

```
σ = 0.20
```

0.2

```
p3D2 = Plot3D[{OPR}, {T, 1, 20},
  {a, 0.5`, 2}, AxesLabel → {Style["T", FontSize → 16, Bold],
    Style["a", FontSize → 16, Bold], Style["OPR", FontSize → 16, Bold]},
  PlotPoints → 50, PlotRange → Automatic, BaselinePosition → Center,
  ImageSize → {500, 400}, ColorFunction → "NeonColors",
  BoxStyle → Directive[Orange, Thick], AxesStyle → Directive[Orange]]
```

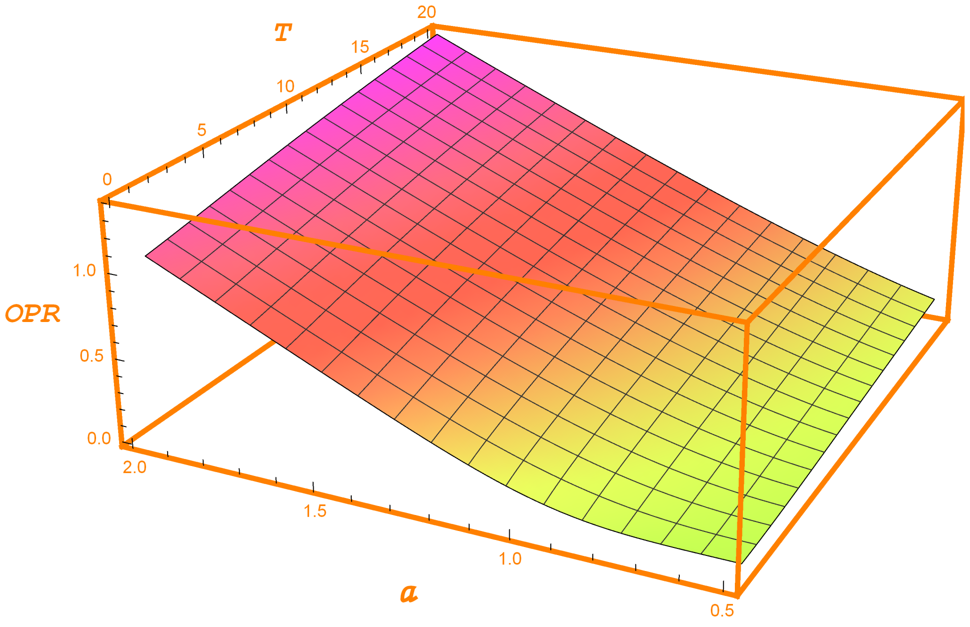


```
T = 5
```

5

```
p2D12 = Plot[{OPR}, {a, 0, 5}, AxesLabel →
   {Style["a", FontSize → 14, Bold], Style["OPR", FontSize → 14, Bold]},
  AxesOrigin → {0, 0}, AxesStyle → Directive[Orange, Thick],
  ImageSize → {300, 250}, ColorFunction → "NeonColors"]
```

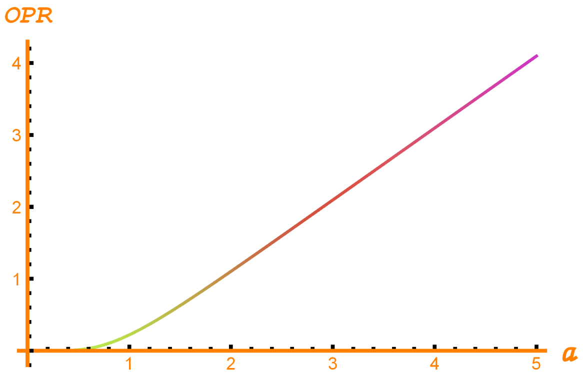

```
Clear[T]
```

```
a = 1
```

```
1
```

```
p2D22 = Plot[OPR, {T, 1, 20}, AxesLabel →
    {Style["T", FontSize → 14, Bold], Style["OPR", FontSize → 14, Bold]},
   AxesOrigin → {0, 0}, AxesStyle → Directive[Orange, Thick],
   ImageSize → {300, 250}, ColorFunction → "NeonColors"]
```

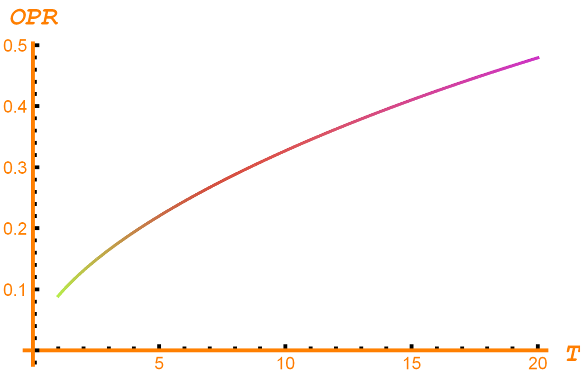


```
RowPlus2 = Row[{p2D12, p2D22}]
```

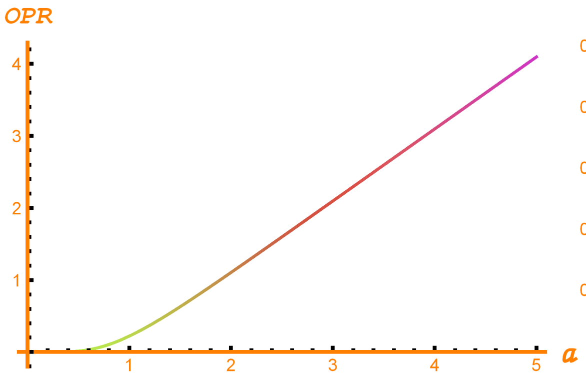

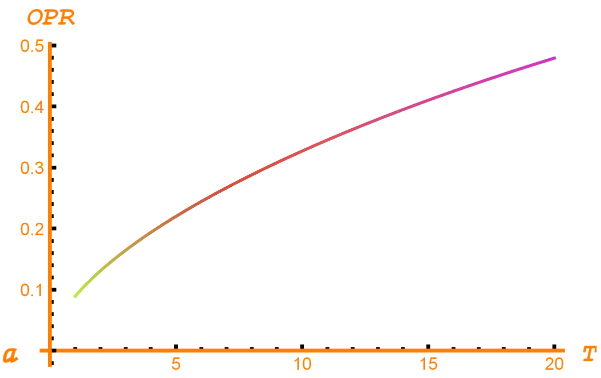

```
Col = Column[{RowPlus2, p3D2}]
```

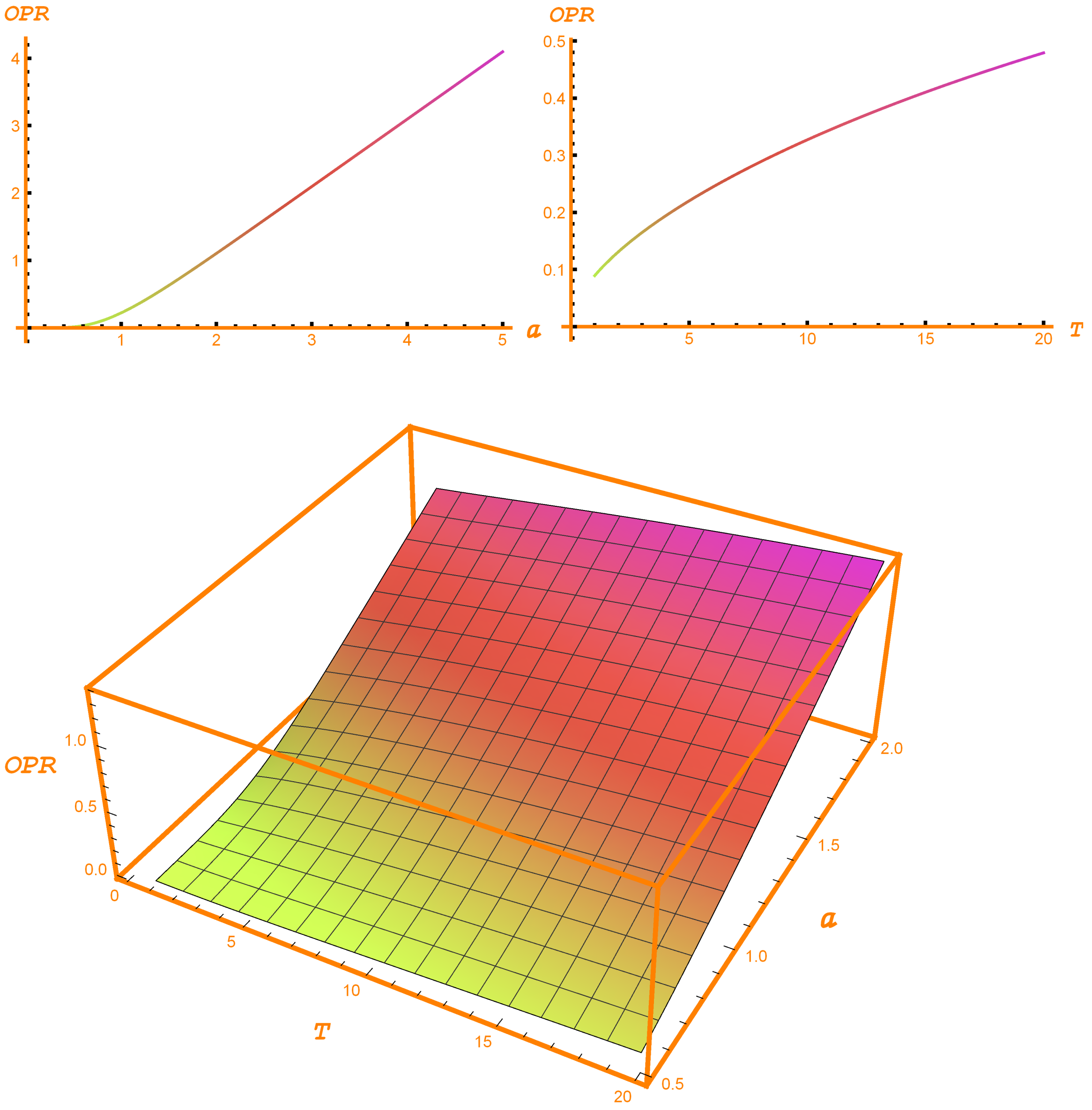

Supplement: S1 Fig — (PDF) [file pone.0125972.s001.pdf]
