# Supplementary material for: Corporate Social Responsibility: A Real Options Approach to the Challenge of Financial Sustainability
Source: PLoS One. 2015 May 4;10(5):e0125972. doi: 10.1371/journal.pone.0125972 (PMC4418608; doi:10.1371/journal.pone.0125972)

S2Fig.: *Mathematica* code for Figure 3

ndist = NormalDistribution[0, 1]

NormalDistribution[0, 1]

Clear[K, A, σ, v, u, T, r, a, c]

$$\mathbf{d1} = \frac{\mathrm{Log}[a] + \left(r + \frac{\sigma^2}{2}\right) * T}{\sigma * \sqrt{T}}$$

$$\frac{T\left(r + \frac{\sigma^2}{2}\right) + \mathrm{Log}[a]}{\sqrt{T}\,\sigma}$$

$$\mathbf{d2} = \mathbf{d1} - \sigma * \sqrt{T}$$

$$-\sqrt{T}\,\sigma + \frac{T\left(r + \frac{\sigma^2}{2}\right) + \mathrm{Log}[a]}{\sqrt{T}\,\sigma}$$

newprojectratio = CDF[ndist, d1] - (1 / a) * Exp[- r * T] CDF[ndist, d2]

$$\frac{1}{2}\,\mathrm{Erfc}\left[-\frac{T\left(r + \frac{\sigma^2}{2}\right) + \mathrm{Log}[a]}{\sqrt{2}\,\sqrt{T}\,\sigma}\right] - \frac{e^{-r\,T}\,\mathrm{Erfc}\left[\frac{\sqrt{T}\,\sigma - \frac{T\left(r + \frac{\sigma^2}{2}\right) + \mathrm{Log}[a]}{\sqrt{T}\,\sigma}}{\sqrt{2}}\right]}{2\,a}$$

r = 0.02

0.02

σ = 0.20

0.2

Clear[tableOP]

```
pr3d = Plot3D[{newprojectratio}, {T, 1, 20},
  {a, 0.5`, 2}, AxesLabel → {Style["T", FontSize → 16, Bold],
    Style["a", FontSize → 16, Bold], Style["NPR", FontSize → 16, Bold]},
  PlotPoints → 50, PlotRange → All, ImageSize → {500, 400},
  ColorFunction → "NeonColors", BoxStyle → Directive[Orange, Thick],
  AxesStyle → Directive[Orange]]
```

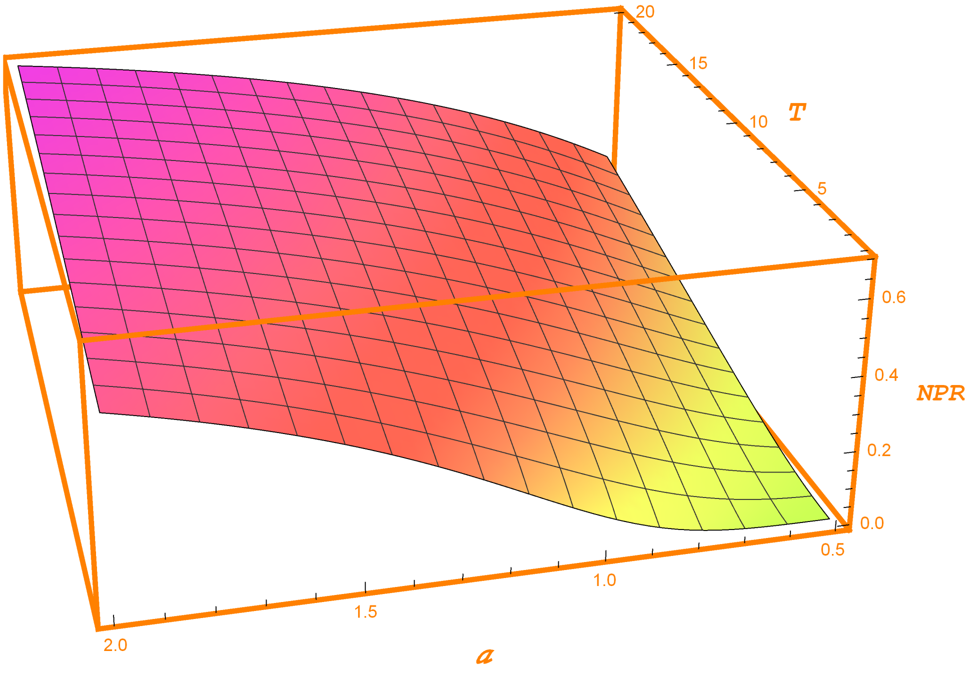


```
T = 5
```

5

```
pla = Plot[{newprojectratio}, {a, 0, 5},
  AxesLabel → {Style["a", FontSize → 14, Bold], Style["NPR", FontSize → 14, Bold]},
  AxesOrigin → {0, 0}, ImageSize → {300, 250},
  AxesStyle → Directive[Orange, Thick], ColorFunction → "NeonColors"]
```

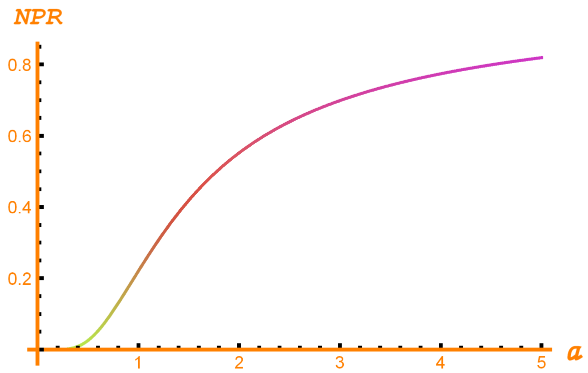

```
Clear[T]

a = 1

1

plT = Plot[newprojectratio, {T, 1, 20},
  AxesLabel → {Style["T", FontSize → 14, Bold], Style["NPR", FontSize → 14, Bold]},
  AxesOrigin → {0, 0},  ImageSize → {300, 250},
  AxesStyle → Directive[Orange, Thick], ColorFunction → "NeonColors"]
```

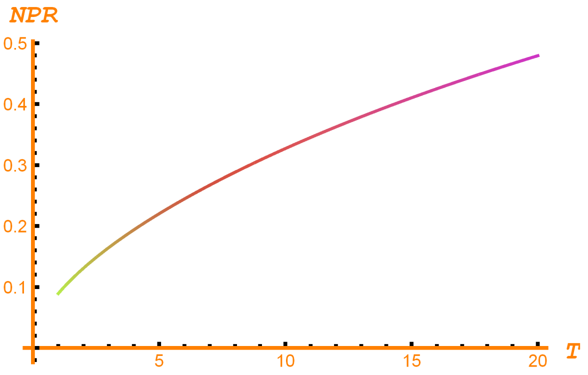


```
Rowplus3 = Row[{pla, plT}]
```

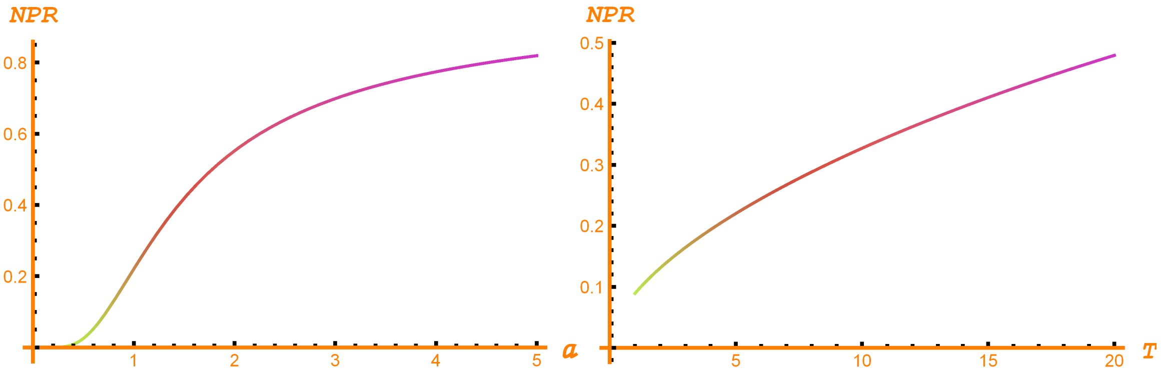

```
Col3 = Column[{Rowplus3, pr3d}]
```

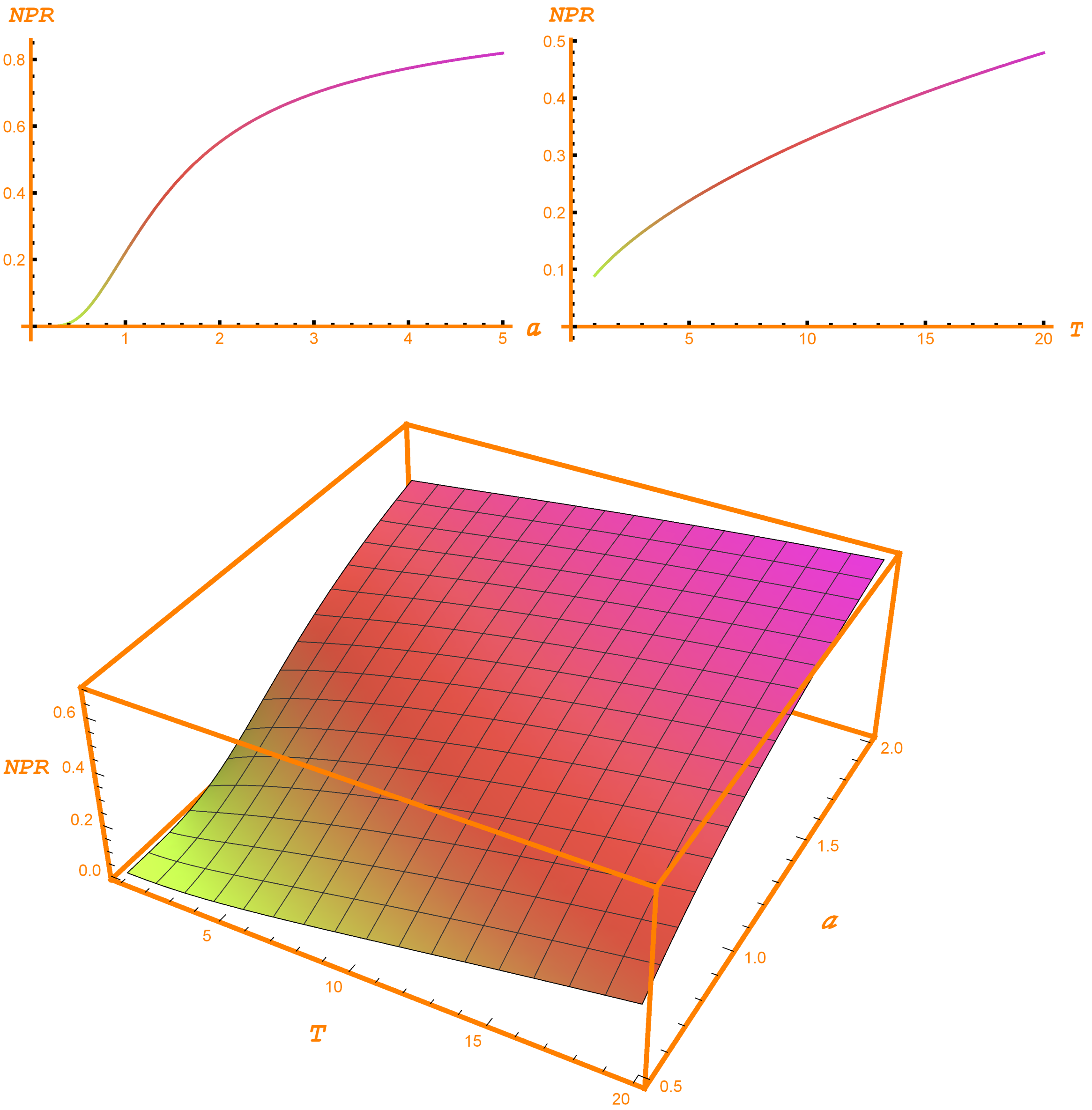

Supplement: S2 Fig — (PDF) [file pone.0125972.s002.pdf]
