# Supplementary material for: Corporate Social Responsibility: A Real Options Approach to the Challenge of Financial Sustainability
Source: PLoS One. 2015 May 4;10(5):e0125972. doi: 10.1371/journal.pone.0125972 (PMC4418608; doi:10.1371/journal.pone.0125972)

S3 Fig.: *Mathematica* code for Figure 4

```
Clear[A, K, σ, a, u, T, r, ck, z, d1, d2]

ndist = NormalDistribution[0, 1]
```

NormalDistribution[0, 1]

$$d1 = \frac{Log[a] + \left(r + \frac{\sigma^2}{2}\right) * T}{\sigma * \sqrt{T}}$$

$$\frac{T\left(r + \frac{\sigma^2}{2}\right) + Log[a]}{\sqrt{T}\ \sigma}$$

$$d2 = d1 - \sigma * \sqrt{T}$$

$$-\sqrt{T}\ \sigma + \frac{T\left(r + \frac{\sigma^2}{2}\right) + Log[a]}{\sqrt{T}\ \sigma}$$

```
ck = a * CDF[ndist, d1] - Exp[-r * T] CDF[ndist, d2]
```

$$\frac{1}{2}\, a\, Erfc\left[-\frac{T\left(r + \frac{\sigma^2}{2}\right) + Log[a]}{\sqrt{2}\ \sqrt{T}\ \sigma}\right] - \frac{1}{2}\, e^{-r\, T}\, Erfc\left[\frac{\sqrt{T}\ \sigma - \frac{T\left(r+\frac{\sigma^2}{2}\right)+Log[a]}{\sqrt{T}\ \sigma}}{\sqrt{2}}\right]$$

```
T = 5
```

5

```
Clear[u]

u = ck - 0.25
```

$$-0.25 + \frac{1}{2}\, a\, Erfc\left[-\frac{5\left(r + \frac{\sigma^2}{2}\right) + Log[a]}{\sqrt{10}\ \sigma}\right] - \frac{1}{2}\, e^{-5\, r}\, Erfc\left[\frac{\sqrt{5}\ \sigma - \frac{5\left(r+\frac{\sigma^2}{2}\right)+Log[a]}{\sqrt{5}\ \sigma}}{\sqrt{2}}\right]$$

```
z5 = Table[FindRoot[u, {a, 10}],
   {r, {0.02, 0.03, 0.05, 0.07, 0.09, 0.10}}, {σ, {0.10, 0.20, 0.30, 0.40, 0.5}}]
```

```
{{{a → 1.13674}, {a → 1.04319}, {a → 0.924159}, {a → 0.80881}, {a → 0.706875}},
 {{a → 1.0952}, {a → 1.00942}, {a → 0.897873}, {a → 0.788673}, {a → 0.691537}},
 {{a → 1.01759}, {a → 0.945857}, {a → 0.848117}, {a → 0.75038}, {a → 0.662256}},
 {{a → 0.946765}, {a → 0.887251}, {a → 0.801895}, {a → 0.714586}, {a → 0.634741}},
 {{a → 0.882172}, {a → 0.833225}, {a → 0.758959}, {a → 0.68113}, {a → 0.608888}},
 {{a → 0.852051}, {a → 0.807821}, {a → 0.738651}, {a → 0.665231}, {a → 0.596553}}}
```

```
T35 = a /. z5
```

```
{{1.13674, 1.04319, 0.924159, 0.80881, 0.706875},
 {1.0952, 1.00942, 0.897873, 0.788673, 0.691537},
 {1.01759, 0.945857, 0.848117, 0.75038, 0.662256},
 {0.946765, 0.887251, 0.801895, 0.714586, 0.634741},
 {0.882172, 0.833225, 0.758959, 0.68113, 0.608888},
 {0.852051, 0.807821, 0.738651, 0.665231, 0.596553}}
```

```
PointsList = {{0.10, 0.02, 1.1367374234634668`}, {0.20, 0.02, 1.043185084253485`},
  {0.30, 0.02, 0.9241591928260203`}, {0.40, 0.02, 0.8088096114670573`},
  {0.50, 0.02, 0.7068747602864467`}, {0.10, 0.03, 1.095199072781985`},
  {0.20, 0.03, 1.0094193289926612`}, {0.30, 0.03, 0.8978733164433204`},
  {0.40, 0.03, 0.7886726180927485`}, {0.50, 0.03, 0.6915370830099835`},
  {0.10, 0.05, 1.0175885402298623`}, {0.20, 0.05, 0.9458573766580637`},
  {0.30, 0.05, 0.848117296787566`}, {0.40, 0.05, 0.7503801507112292`},
  {0.50, 0.05, 0.6622561414748724`}, {0.10, 0.07, 0.9467648230894323`},
  {0.20, 0.07, 0.8872511411422819`}, {0.30, 0.07, 0.8018948745315063`},
  {0.40, 0.07, 0.7145864543891784`}, {0.50, 0.07, 0.6347414687885969`},
  {0.10, 0.09, 0.8821718100493979`}, {0.20, 0.09, 0.83322520144229`},
  {0.30, 0.09, 0.7589588229966231`}, {0.40, 0.09, 0.6811302694706995`},
  {0.50, 0.09, 0.6088879623267234`}, {0.10, 0.10, 0.8520511077924986`},
  {0.20, 0.10, 0.8078209832599433`}, {0.30, 0.10, 0.7386510951798643`},
  {0.40, 0.10, 0.6652314831592241`}, {0.50, 0.10, 0.5965532024267727`}}
```

```
{{0.1, 0.02, 1.13674}, {0.2, 0.02, 1.04319}, {0.3, 0.02, 0.924159},
 {0.4, 0.02, 0.80881}, {0.5, 0.02, 0.706875}, {0.1, 0.03, 1.0952},
 {0.2, 0.03, 1.00942}, {0.3, 0.03, 0.897873}, {0.4, 0.03, 0.788673},
 {0.5, 0.03, 0.691537}, {0.1, 0.05, 1.01759}, {0.2, 0.05, 0.945857},
 {0.3, 0.05, 0.848117}, {0.4, 0.05, 0.75038}, {0.5, 0.05, 0.662256},
 {0.1, 0.07, 0.946765}, {0.2, 0.07, 0.887251}, {0.3, 0.07, 0.801895},
 {0.4, 0.07, 0.714586}, {0.5, 0.07, 0.634741}, {0.1, 0.09, 0.882172},
 {0.2, 0.09, 0.833225}, {0.3, 0.09, 0.758959}, {0.4, 0.09, 0.68113},
 {0.5, 0.09, 0.608888}, {0.1, 0.1, 0.852051}, {0.2, 0.1, 0.807821},
 {0.3, 0.1, 0.738651}, {0.4, 0.1, 0.665231}, {0.5, 0.1, 0.596553}}
```

```
BoxPoints2 = ListPointPlot3D[PointsList,
  PlotStyle → {Blue, PointSize[0.03`], PlotPoints → 50},
  Axes → {True, True, True}, PlotRange → Automatic, BoxRatios → {5, 5, 8},
  AxesLabel → {Style["σ", FontSize → 25], Style["r", FontSize → 25],
    Style["a", FontSize → 25]}, BoxStyle → Directive[Orange, Dashed],
  ColorFunction → "NeonColors", AxesStyle → Directive[Orange, Dashed]]
```

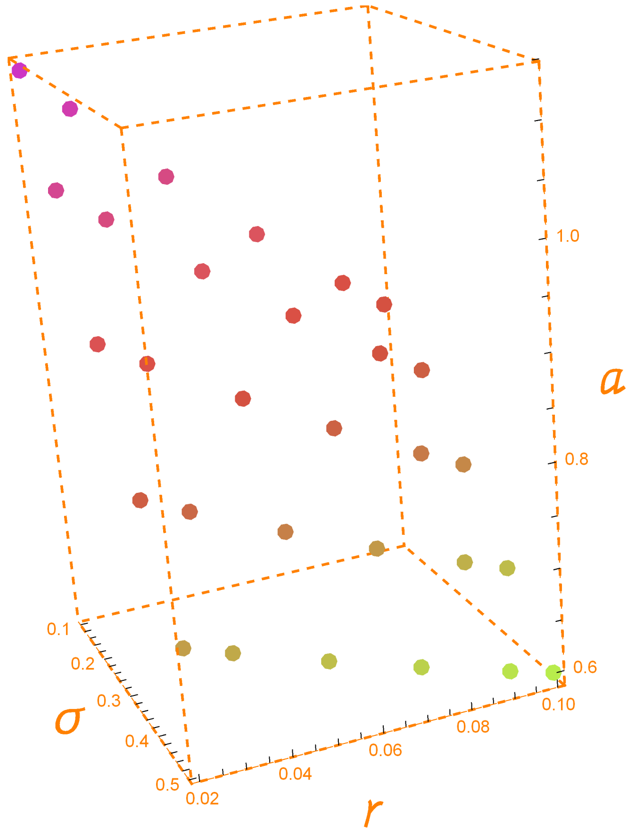

Supplement: S3 Fig — (PDF) [file pone.0125972.s003.pdf]
