# Supplementary material for: Corporate Social Responsibility: A Real Options Approach to the Challenge of Financial Sustainability
Source: PLoS One. 2015 May 4;10(5):e0125972. doi: 10.1371/journal.pone.0125972 (PMC4418608; doi:10.1371/journal.pone.0125972)

S4 Fig.; *Mathematica* code for Figure 5

```
Clear[A, K, a, d1, d2, ct, σ, v, y, T, r, ϖ, y, TableTime, ListTime]
```

```
ndist = NormalDistribution[0, 1]
```

```
NormalDistribution[0, 1]
```

$$d1 = \frac{\text{Log}[a] + \left(r + \frac{\sigma^2}{2}\right) * T}{\sigma * \sqrt{T}}$$

$$\frac{T\left(r + \frac{\sigma^2}{2}\right) + \text{Log}[a]}{\sqrt{T}\ \sigma}$$

$$d2 = d1 - \sigma * \sqrt{T}$$

$$-\sqrt{T}\ \sigma + \frac{T\left(r + \frac{\sigma^2}{2}\right) + \text{Log}[a]}{\sqrt{T}\ \sigma}$$

```
ct = a * CDF[ndist, d1] - Exp[-r * T] CDF[ndist, d2]
```

$$\frac{1}{2}\, a\, \text{Erfc}\left[-\frac{T\left(r + \frac{\sigma^2}{2}\right) + \text{Log}[a]}{\sqrt{2}\ \sqrt{T}\ \sigma}\right] - \frac{1}{2}\, e^{-r\,T}\, \text{Erfc}\left[\frac{\sqrt{T}\ \sigma - \frac{T\left(r + \frac{\sigma^2}{2}\right) + \text{Log}[a]}{\sqrt{T}\ \sigma}}{\sqrt{2}}\right]$$

```
Simplify[%]
```

$$\frac{1}{2}\left(-e^{-r\,T}\, \text{Erfc}\left[\frac{T\left(-2\,r + \sigma^2\right) - 2\,\text{Log}[a]}{2\,\sqrt{2}\ \sqrt{T}\ \sigma}\right] + a\, \text{Erfc}\left[-\frac{T\left(r + \frac{\sigma^2}{2}\right) + \text{Log}[a]}{\sqrt{2}\ \sqrt{T}\ \sigma}\right]\right)$$

```
a = 1
```

```
1
```

```
y = ct - 0.25
```

$$-0.25 + \frac{1}{2}\, \text{Erfc}\left[-\frac{\sqrt{T}\ \left(r + \frac{\sigma^2}{2}\right)}{\sqrt{2}\ \sigma}\right] - \frac{1}{2}\, e^{-r\,T}\, \text{Erfc}\left[\frac{\sqrt{T}\ \sigma - \frac{\sqrt{T}\ \left(r + \frac{\sigma^2}{2}\right)}{\sigma}}{\sqrt{2}}\right]$$

```
TableTime = Table[FindRoot[y, {T, 1}],
   {r, {0.02, 0.03, 0.05, 0.07, 0.09, 0.10}}, {σ, {0.1, 0.2, 0.3, 0.4, 0.5}}]
```

```
{{{T → 11.5058}, {T → 6.24066}, {T → 3.52799}, {T → 2.19353}, {T → 1.47593}},
 {{T → 8.41311}, {T → 5.23194}, {T → 3.18184}, {T → 2.05438}, {T → 1.41151}},
 {{T → 5.43099}, {T → 3.94797}, {T → 2.6598}, {T → 1.82332}, {T → 1.29834}},
 {{T → 3.98885}, {T → 3.16408}, {T → 2.2842}, {T → 1.63904}, {T → 1.20206}},
 {{T → 3.14321}, {T → 2.63577}, {T → 2.0007}, {T → 1.4885}, {T → 1.11911}},
 {{T → 2.84003}, {T → 2.43142}, {T → 1.88347}, {T → 1.42309}, {T → 1.08179}}}
```

```
TTcln = T /. TableTime

{{11.5058, 6.24066, 3.52799, 2.19353, 1.47593},
 {8.41311, 5.23194, 3.18184, 2.05438, 1.41151},
 {5.43099, 3.94797, 2.6598, 1.82332, 1.29834},
 {3.98885, 3.16408, 2.2842, 1.63904, 1.20206},
 {3.14321, 2.63577, 2.0007, 1.4885, 1.11911},
 {2.84003, 2.43142, 1.88347, 1.42309, 1.08179}}

TableForm[TTcln]
```

11.5058	6.24066	3.52799	2.19353	1.47593
8.41311	5.23194	3.18184	2.05438	1.41151
5.43099	3.94797	2.6598	1.82332	1.29834
3.98885	3.16408	2.2842	1.63904	1.20206
3.14321	2.63577	2.0007	1.4885	1.11911
2.84003	2.43142	1.88347	1.42309	1.08179

```
TableForm[TTcln,
 TableHeadings → {{"0.02", "0.03", "0.05", "0.07", "0.09", "0.10"},
   {"0.10", "0.20", "0.30", "0.40", "0.50"}}]
```

	0.10	0.20	0.30	0.40	0.50
0.02	11.5058	6.24066	3.52799	2.19353	1.47593
0.03	8.41311	5.23194	3.18184	2.05438	1.41151
0.05	5.43099	3.94797	2.6598	1.82332	1.29834
0.07	3.98885	3.16408	2.2842	1.63904	1.20206
0.09	3.14321	2.63577	2.0007	1.4885	1.11911
0.10	2.84003	2.43142	1.88347	1.42309	1.08179

```
ListTime = {{0.20, 0.02, 6.240660698136456`}, {0.20, 0.03, 5.231936777148886`},
   {0.20, 0.05, 3.9479712100901128`}, {0.20, 0.07, 3.164080739705138`},
   {0.20, 0.09, 2.6357686545645267`}, {0.20, 0.10, 2.4314208959186323`},
   {0.30, 0.02, 3.527989458252755`}, {0.3, 0.03, 3.181837396962509`},
   {0.30, 0.05, 2.659798738425263`}, {0.30, 0.07, 2.284203838480975`},
   {0.30, 0.09, 2.0007043759295184`}, {0.30, 0.10, 1.883473961166831`},
   {0.40, 0.02, 2.19352983145628`}, {0.40, 0.03, 2.054378680216739`},
   {0.40, 0.05, 1.8233200657326074`}, {0.40, 0.07, 1.6390389150300761`},
   {0.40, 0.09, 1.4884985378254998`}, {0.40, 0.10, 1.4230852975302104`},
   {0.50, 0.02, 1.475926037243902`}, {0.50, 0.03, 1.4115130285324415`},
   {0.50, 0.05, 1.2983402643432527`}, {0.50, 0.07, 1.2020648277934975`},
   {0.50, 0.09, 1.11911`}, {0.50, 0.10, 1.081788987028241`}}

{{0.2, 0.02, 6.24066}, {0.2, 0.03, 5.23194}, {0.2, 0.05, 3.94797},
 {0.2, 0.07, 3.16408}, {0.2, 0.09, 2.63577}, {0.2, 0.1, 2.43142},
 {0.3, 0.02, 3.52799}, {0.3, 0.03, 3.18184}, {0.3, 0.05, 2.6598},
 {0.3, 0.07, 2.2842}, {0.3, 0.09, 2.0007}, {0.3, 0.1, 1.88347},
 {0.4, 0.02, 2.19353}, {0.4, 0.03, 2.05438}, {0.4, 0.05, 1.82332},
 {0.4, 0.07, 1.63904}, {0.4, 0.09, 1.4885}, {0.4, 0.1, 1.42309},
 {0.5, 0.02, 1.47593}, {0.5, 0.03, 1.41151}, {0.5, 0.05, 1.29834},
 {0.5, 0.07, 1.20206}, {0.5, 0.09, 1.11911}, {0.5, 0.1, 1.08179}}
```

```
plotListTime = ListPointPlot3D[ListTime,
  PlotStyle → {Orange, PointSize[0.03`], PlotPoints → 50},
  Axes → True, PlotRange → Automatic, BoxRatios → {5, 5, 8},
  AxesLabel → {Style["σ", FontSize → 25, Bold], Style["r", FontSize → 25, Bold],
    Style["T", FontSize → 25, Bold]}, BoxStyle → Directive[Orange, Dashed],
  ColorFunction → "NeonColors", AxesStyle → Directive[Orange, Dashed]]
```

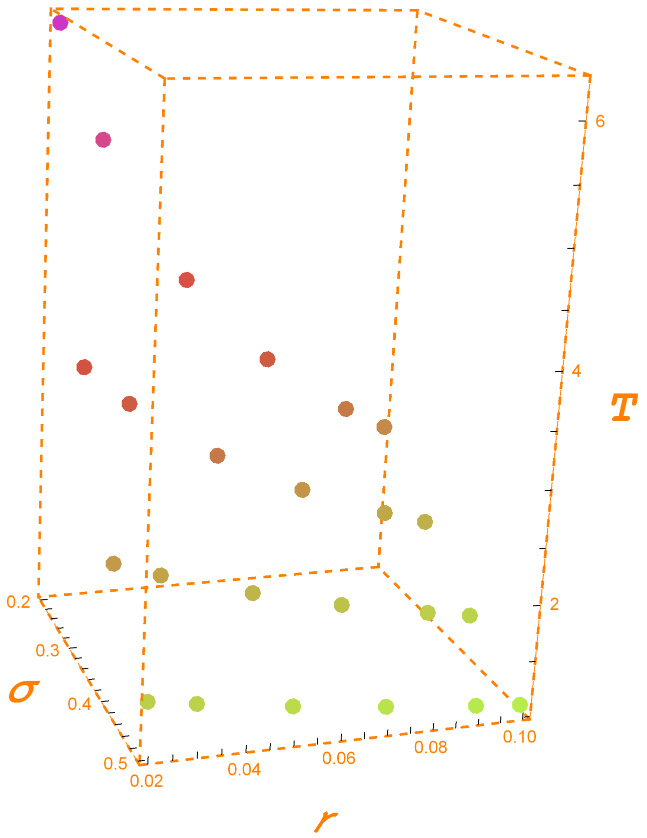

Supplement: S4 Fig — (PDF) [file pone.0125972.s004.pdf]
