# Supplementary material for: Corporate Social Responsibility: A Real Options Approach to the Challenge of Financial Sustainability
Source: PLoS One. 2015 May 4;10(5):e0125972. doi: 10.1371/journal.pone.0125972 (PMC4418608; doi:10.1371/journal.pone.0125972)

S5 Fig: *Mathematica* code for Figure 6

```
Clear[A, K, a, d1, d2, ct, σ, v, y, T, r, ϖ, pik, ct, tabley]

ndist = NormalDistribution[0, 1]

NormalDistribution[0, 1]

$Assumptions = σ > 0

σ > 0
```

$$d1 = \frac{\text{Log}[a] + \left(r + \frac{\sigma^2}{2}\right) * T}{\sigma * \sqrt{T}}$$

$$\frac{T\left(r + \frac{\sigma^2}{2}\right) + \text{Log}[a]}{\sqrt{T}\ \sigma}$$

$$d2 = d1 - \sigma * \sqrt{T}$$

$$-\sqrt{T}\ \sigma + \frac{T\left(r + \frac{\sigma^2}{2}\right) + \text{Log}[a]}{\sqrt{T}\ \sigma}$$

```
ct = a * CDF[ndist, d1] - Exp[-r * T] CDF[ndist, d2]
```

$$\frac{1}{2}\, a\, \text{Erfc}\left[-\frac{T\left(r + \frac{\sigma^2}{2}\right) + \text{Log}[a]}{\sqrt{2}\ \sqrt{T}\ \sigma}\right] - \frac{1}{2}\, e^{-r\,T}\, \text{Erfc}\left[\frac{\sqrt{T}\ \sigma - \frac{T\left(r+\frac{\sigma^2}{2}\right)+\text{Log}[a]}{\sqrt{T}\ \sigma}}{\sqrt{2}}\right]$$

```
Simplify[%]
```

$$\frac{1}{2}\left(-e^{-r\,T}\, \text{Erfc}\left[\frac{T\left(-2\,r + \sigma^2\right) - 2\,\text{Log}[a]}{2\sqrt{2}\ \sqrt{T}\ \sigma}\right] + a\, \text{Erfc}\left[-\frac{T\left(r + \frac{\sigma^2}{2}\right) + \text{Log}[a]}{\sqrt{2}\ \sqrt{T}\ \sigma}\right]\right)$$

```
r = 0.02

0.02

Tbvol020 = Table[FindRoot[ct == 0.2, {σ, 0.6}],
   {a, {0.30, 0.50, 0.60, 0.75, 0.90}}, {T, 2, 7, 1}]

{{{σ → 1.81981}, {σ → 1.48048}, {σ → 1.27743}, {σ → 1.13835},
   {σ → 1.0353}, {σ → 0.954908}}, {{σ → 1.06652}, {σ → 0.863899},
   {σ → 0.742125}, {σ → 0.658336}, {σ → 0.59597}, {σ → 0.547087}},
 {{σ → 0.863256}, {σ → 0.697221}, {σ → 0.597141}, {σ → 0.528071},
   {σ → 0.476499}, {σ → 0.435947}}, {{σ → 0.631471}, {σ → 0.506705},
   {σ → 0.431002}, {σ → 0.378395}, {σ → 0.338832}, {σ → 0.307486}},
 {{σ → 0.443805}, {σ → 0.351551}, {σ → 0.294829},
   {σ → 0.254843}, {σ → 0.224293}, {σ → 0.199659}}}
```

```
TableForm[Tbvol020]
```

σ → 1.81981	σ → 1.48048	σ → 1.27743	σ → 1.13835	σ → 1.0353	σ → 0.[illegible]
σ → 1.06652	σ → 0.863899	σ → 0.742125	σ → 0.658336	σ → 0.59597	σ → 0.[illegible]
σ → 0.863256	σ → 0.697221	σ → 0.597141	σ → 0.528071	σ → 0.476499	σ → 0.[illegible]
σ → 0.631471	σ → 0.506705	σ → 0.431002	σ → 0.378395	σ → 0.338832	σ → 0.[illegible]
σ → 0.443805	σ → 0.351551	σ → 0.294829	σ → 0.254843	σ → 0.224293	σ → 0.[illegible]

```
Tablevol020 = σ /. Tbvol020
```

```
{{1.81981, 1.48048, 1.27743, 1.13835, 1.0353, 0.954908},
 {1.06652, 0.863899, 0.742125, 0.658336, 0.59597, 0.547087},
 {0.863256, 0.697221, 0.597141, 0.528071, 0.476499, 0.435947},
 {0.631471, 0.506705, 0.431002, 0.378395, 0.338832, 0.307486},
 {0.443805, 0.351551, 0.294829, 0.254843, 0.224293, 0.199659}}
```

```
TableForm[Tablevol020, TableHeadings →
  {{"0.3", "0.5", "0.6", "0.75", "0.9"}, {"2", "3", "4", "5", "6", "7"}}]
```

	2	3	4	5	6	7
0.3	1.81981	1.48048	1.27743	1.13835	1.0353	0.954908
0.5	1.06652	0.863899	0.742125	0.658336	0.59597	0.547087
0.6	0.863256	0.697221	0.597141	0.528071	0.476499	0.435947
0.75	0.631471	0.506705	0.431002	0.378395	0.338832	0.307486
0.9	0.443805	0.351551	0.294829	0.254843	0.224293	0.199659

```
PointsListσ = {{2, 0.3, 1.8198}, {3, 0.3, 1.4805}, {4, 0.3, 1.2774},
  {5, 0.3, 1.1384}, {6, 0.3, 1.0353}, {7, 0.3, 0.9549}, {2, 0.5, 1.0665},
  {3, 0.5, 0.8639}, {4, 0.5, 0.7421}, {5, 0.5, 0.6583}, {6, 0.5, 0.5960},
  {7, 0.5, 0.5471}, {2, 0.6, 0.8633}, {3, 0.6, 0.6972}, {4, 0.6, 0.5971},
  {5, 0.6, 0.5281}, {6, 0.6, 0.4765}, {7, 0.6, 0.4359}, {2, 0.75, 0.6315},
  {3, 0.75, 0.5067}, {4, 0.75, 0.4310}, {5, 0.75, 0.3784},
  {6, 0.75, 0.3388}, {7, 0.75, 0.3075}, {2, 0.9, 0.4483}, {3, 0.9, 0.3516},
  {4, 0.9, 0.2948}, {5, 0.9, 0.2548}, {6 , 0.9, 0.2243}, {7, 0.9, 0.1997}}
```

```
{{2, 0.3, 1.8198}, {3, 0.3, 1.4805}, {4, 0.3, 1.2774},
 {5, 0.3, 1.1384}, {6, 0.3, 1.0353}, {7, 0.3, 0.9549}, {2, 0.5, 1.0665},
 {3, 0.5, 0.8639}, {4, 0.5, 0.7421}, {5, 0.5, 0.6583}, {6, 0.5, 0.596},
 {7, 0.5, 0.5471}, {2, 0.6, 0.8633}, {3, 0.6, 0.6972}, {4, 0.6, 0.5971},
 {5, 0.6, 0.5281}, {6, 0.6, 0.4765}, {7, 0.6, 0.4359}, {2, 0.75, 0.6315},
 {3, 0.75, 0.5067}, {4, 0.75, 0.431}, {5, 0.75, 0.3784},
 {6, 0.75, 0.3388}, {7, 0.75, 0.3075}, {2, 0.9, 0.4483}, {3, 0.9, 0.3516},
 {4, 0.9, 0.2948}, {5, 0.9, 0.2548}, {6, 0.9, 0.2243}, {7, 0.9, 0.1997}}
```

```
BoxPointsσ = ListPointPlot3D[PointsListσ,
  PlotStyle → {Blue, PointSize[0.03`], PlotPoints → 50},
  Axes → {True, True, True}, PlotRange → Automatic, BoxRatios → {5, 5, 7},
  AxesLabel → {Style["T", FontSize → 18, Bold], Style["a", FontSize → 25, Bold],
    Style["σ", FontSize → 18, Bold]}, ColorFunction → "NeonColors",
  BoxStyle → Directive[Orange, Dashed], AxesStyle → Directive[Orange, Dashed]]
```

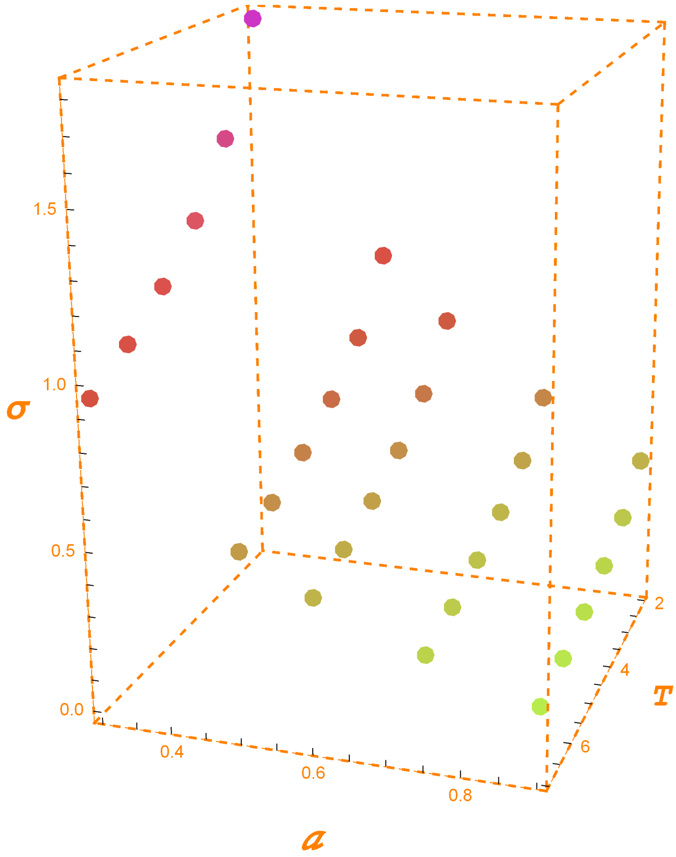

Supplement: S5 Fig — (PDF) [file pone.0125972.s005.pdf]
