# Supplementary material for: Corporate Social Responsibility: A Real Options Approach to the Challenge of Financial Sustainability
Source: PLoS One. 2015 May 4;10(5):e0125972. doi: 10.1371/journal.pone.0125972 (PMC4418608; doi:10.1371/journal.pone.0125972)

S6Fig: *Mathematica* code for Figure 7

```
Clear[ndist, υ, T, sbs, dx1, dx2, θ, osr, csv, SPV, CPV]
```

SPV = Savings Present Value

CPV = Cost Present Value

sbs = Substitution option

osr = opportunity savings ratio

```
$Aborted
```

```
$Aborted
```

csv = cost/savings ratio

```
ndist = NormalDistribution[0, 1]
```

```
NormalDistribution[0, 1]
```

$$\theta = \sqrt{\sigma SPV^2 + \sigma CPV^2 - 2 * \rho * \sigma SPV * \sigma CPV}$$

$$\sqrt{\sigma CPV^2 - 2\,\rho\,\sigma CPV\,\sigma SPV + \sigma SPV^2}$$

Let us apply Margrabe's formula:

$$dx1 = \frac{\text{Log}\left[\frac{1}{csv}\right] + \left(\frac{\upsilon^2}{2}\right) * T}{\upsilon * \sqrt{T}}$$

$$\frac{\frac{T\,\upsilon^2}{2} + \text{Log}\left[\frac{1}{csv}\right]}{\sqrt{T}\;\upsilon}$$

$$dx2 = dx1 - \upsilon * \sqrt{T}$$

$$-\sqrt{T}\;\upsilon + \frac{\frac{T\,\upsilon^2}{2} + \text{Log}\left[\frac{1}{csv}\right]}{\sqrt{T}\;\upsilon}$$

```
osr = CDF[ndist, dx1] - csv * CDF[ndist, dx2]
```

$$\frac{1}{2}\,\text{Erfc}\left[-\frac{\frac{T\,\upsilon^2}{2} + \text{Log}\left[\frac{1}{csv}\right]}{\sqrt{2}\;\sqrt{T}\;\upsilon}\right] - \frac{1}{2}\,csv\;\text{Erfc}\left[\frac{\sqrt{T}\;\upsilon - \frac{\frac{T\,\upsilon^2}{2}+\text{Log}\left[\frac{1}{csv}\right]}{\sqrt{T}\;\upsilon}}{\sqrt{2}}\right]$$

```
csv = 1
```

```
1
```

```
PlotOSR = Plot3D[{osr}, {T, 1, 20},
  {υ, 0.5`, 2}, AxesLabel → {Style["T", FontSize → 16, Bold],
    Style["υ", FontSize → 16, Bold], Style["OSR", FontSize → 16, Bold]},
  BoxStyle → Directive[Orange], PlotPoints → 50, PlotRange → All,
  MaxRecursion → 15, ColorFunction → "TemperatureMap",
  AxesStyle → Directive[Orange], ImageSize → {500, 500}]
```

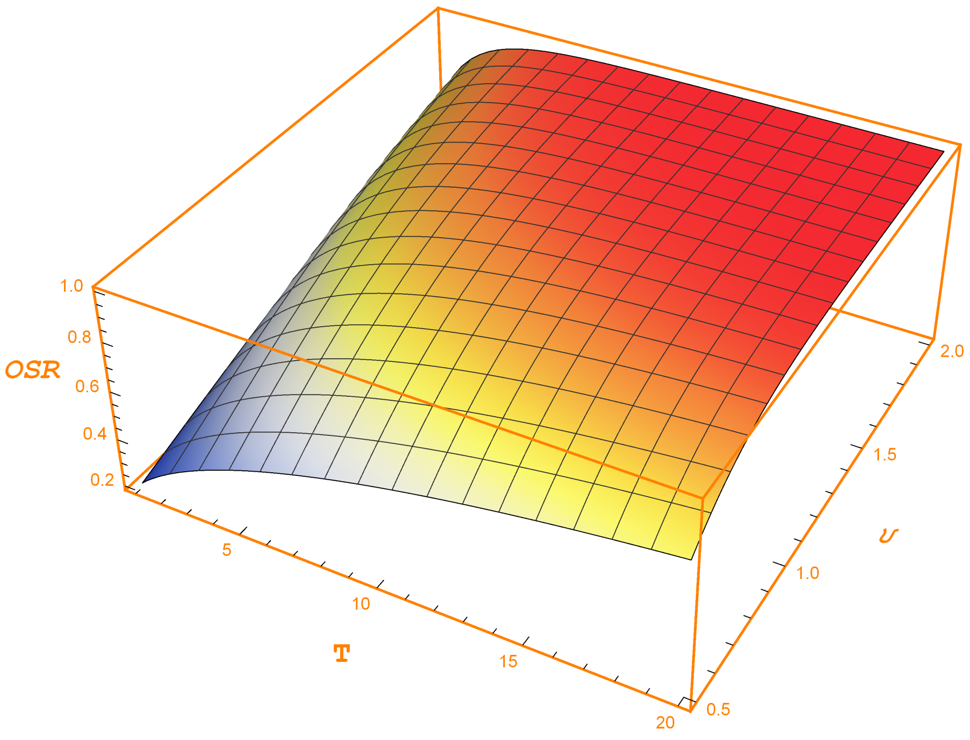

```
T = 5
osra = Plot[{osr}, {υ, 0, 2}, AxesLabel →
    {Style["υ", FontSize → 16, Bold], Style["OSR", FontSize → 16, Bold]},
   AxesOrigin → {0, 0}, AxesStyle → Directive[Orange, Thick], PlotPoints → 50,
   ColorFunction → "TemperatureMap", ImageSize → {300, 250}]
```

5

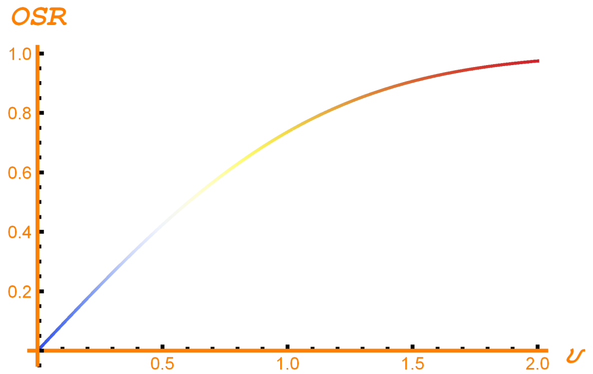


```
Clear[T]
```

```
υ = 0.2
```

0.2

```
osrT = Plot[{osr}, {T, 0, 20}, AxesLabel →
    {Style["T", FontSize → 16, Bold], Style["OSR", FontSize → 16, Bold]},
   AxesOrigin → {0, 0}, AxesStyle → Directive[Orange, Thick], PlotPoints → 50,
   ColorFunction → "TemperatureMap", ImageSize → {300, 250}]
```

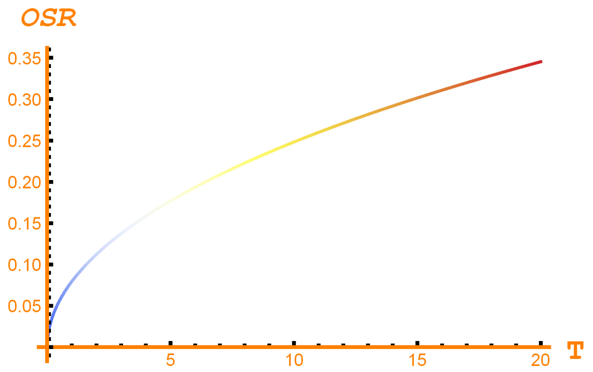

```
RowosraT = Row[{osra, osrT}]
```

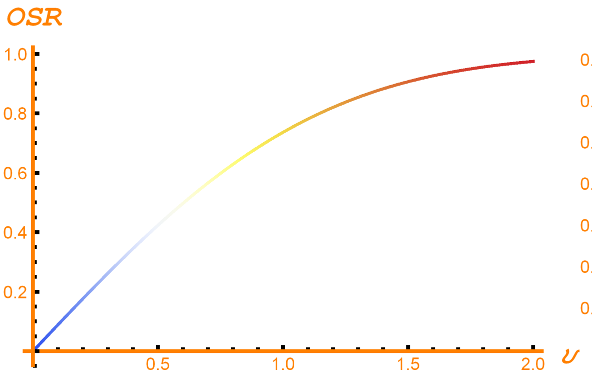

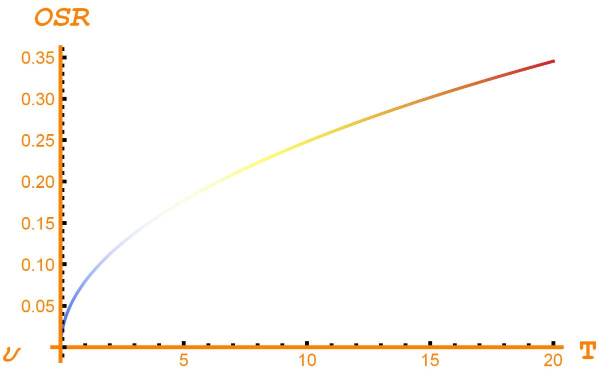

```
Column[{RowosraT, PlotOSR}]
```

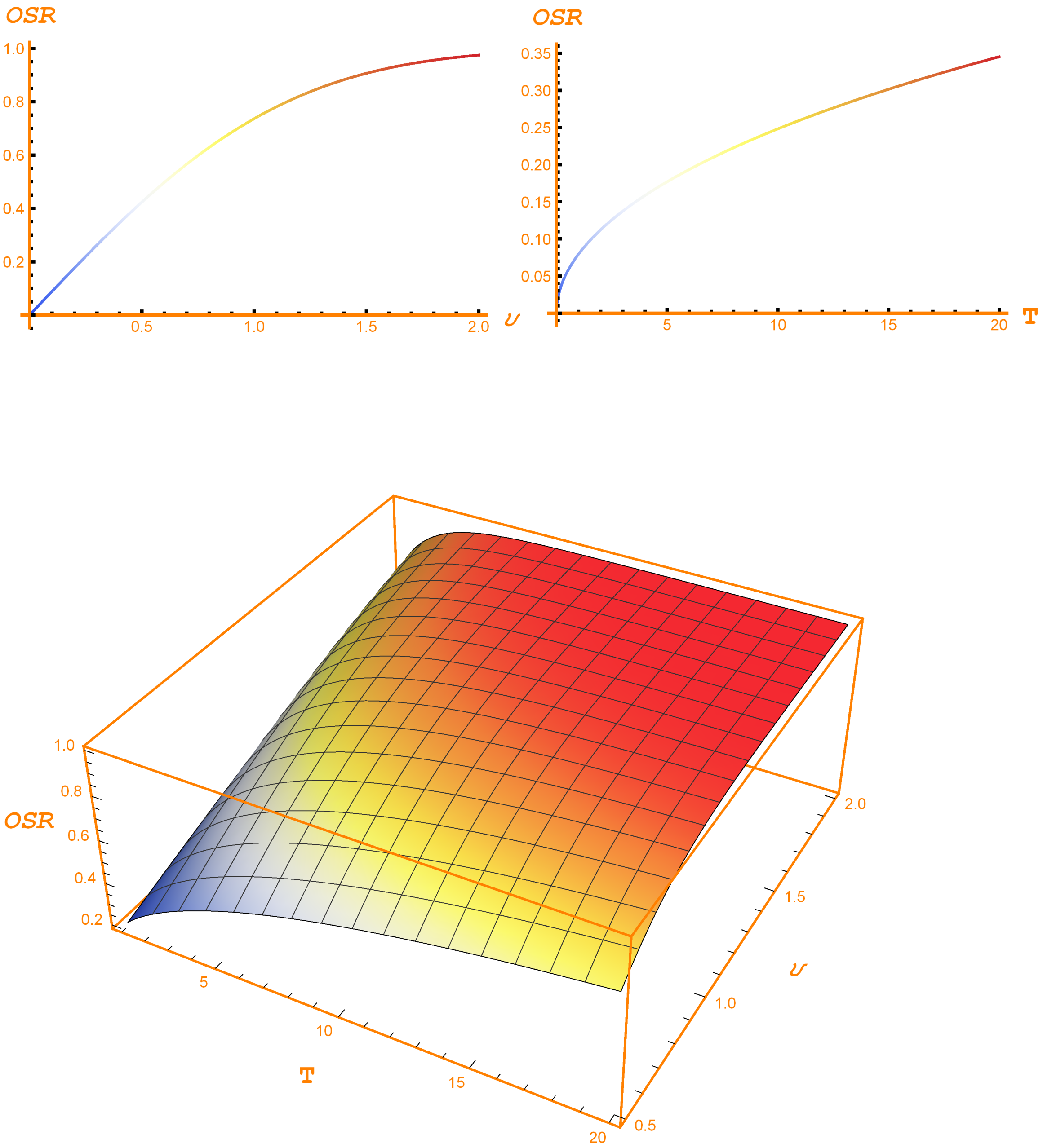

Supplement: S6 Fig — (PDF) [file pone.0125972.s006.pdf]
