# Supplementary material for: Corporate Social Responsibility: A Real Options Approach to the Challenge of Financial Sustainability
Source: PLoS One. 2015 May 4;10(5):e0125972. doi: 10.1371/journal.pone.0125972 (PMC4418608; doi:10.1371/journal.pone.0125972)
Supplement: S7 Fig — (PDF) [file pone.0125972.s007.pdf]

S7Fig: *Mathematica* code for Figure 8

```
Clear[ndist, υ, T, sbs, dx1, dx2, θ, osr, csv, SPV, CPV]
```

SPV = Savings Present Value

CPV = Cost Present Value

sbs = Substitution option

osr = opportunity savings ratio

csv = cost/savings ratio

```
ndist = NormalDistribution[0, 1]
```

```
NormalDistribution[0, 1]
```

Let us apply Margrabe's formula:

$$dx1 = \frac{\text{Log}\left[\frac{1}{csv}\right] + \left(\frac{\upsilon^2}{2}\right) * T}{\upsilon * \sqrt{T}}$$

$$\frac{\frac{T\,\upsilon^2}{2} + \text{Log}\left[\frac{1}{csv}\right]}{\sqrt{T}\ \upsilon}$$

$$dx2 = dx1 - \upsilon * \sqrt{T}$$

$$-\sqrt{T}\ \upsilon + \frac{\frac{T\,\upsilon^2}{2} + \text{Log}\left[\frac{1}{csv}\right]}{\sqrt{T}\ \upsilon}$$

```
osr = CDF[ndist, dx1] - csv * CDF[ndist, dx2]
```

$$\frac{1}{2}\,\text{Erfc}\left[-\frac{\frac{T\,\upsilon^2}{2} + \text{Log}\left[\frac{1}{csv}\right]}{\sqrt{2}\ \sqrt{T}\ \upsilon}\right] - \frac{1}{2}\,csv\ \text{Erfc}\left[\frac{\sqrt{T}\ \upsilon - \frac{\frac{T\,\upsilon^2}{2}+\text{Log}\left[\frac{1}{csv}\right]}{\sqrt{T}\ \upsilon}}{\sqrt{2}}\right]$$

```
ξ = osr - 0.20
```

$$-0.2 + \frac{1}{2}\,\text{Erfc}\left[-\frac{\frac{T\,\upsilon^2}{2} + \text{Log}\left[\frac{1}{csv}\right]}{\sqrt{2}\ \sqrt{T}\ \upsilon}\right] - \frac{1}{2}\,csv\ \text{Erfc}\left[\frac{\sqrt{T}\ \upsilon - \frac{\frac{T\,\upsilon^2}{2}+\text{Log}\left[\frac{1}{csv}\right]}{\sqrt{T}\ \upsilon}}{\sqrt{2}}\right]$$

```
Z = Table[FindRoot[ξ, {csv, 1}],
  {T, {1, 2, 3, 4, 5}}, {υ, {0.10, 0.20, 0.30, 0.40, 0.50}}]
```

```
{{{csv → 0.800405}, {csv → 0.814215}, {csv → 0.851429},
  {csv → 0.911003}, {csv → 0.993592}}, {{csv → 0.803316},
  {csv → 0.843438}, {csv → 0.928855}, {csv → 1.06162}, {csv → 1.25205}},
 {{csv → 0.8082}, {csv → 0.876311}, {csv → 1.0127}, {csv → 1.22973},
  {csv → 1.55822}}, {{csv → 0.814215}, {csv → 0.911003},
  {csv → 1.10197}, {csv → 1.41703}, {csv → 1.92197}}, {{csv → 0.820931},
  {csv → 0.947004}, {csv → 1.19682}, {csv → 1.62602}, {csv → 2.35478}}}
```

```
Zclean = csv /. Z
```

```
{{0.800405, 0.814215, 0.851429, 0.911003, 0.993592},
 {0.803316, 0.843438, 0.928855, 1.06162, 1.25205},
 {0.8082, 0.876311, 1.0127, 1.22973, 1.55822},
 {0.814215, 0.911003, 1.10197, 1.41703, 1.92197},
 {0.820931, 0.947004, 1.19682, 1.62602, 2.35478}}
```

```
csv /. Table[FindRoot[ξ, {csv, 1}], {T, 1, 10, 1}, {υ, 0.1`, 0.5`, 0.1`}]
```

```
{{0.800405, 0.814215, 0.851429, 0.911003, 0.993592},
 {0.803316, 0.843438, 0.928855, 1.06162, 1.25205},
 {0.8082, 0.876311, 1.0127, 1.22973, 1.55822},
 {0.814215, 0.911003, 1.10197, 1.41703, 1.92197},
 {0.820931, 0.947004, 1.19682, 1.62602, 2.35478},
 {0.828121, 0.984139, 1.29759, 1.85945, 2.86987},
 {0.83565, 1.02235, 1.40472, 2.12033, 3.48267},
 {0.843438, 1.06162, 1.51864, 2.41196, 4.21122},
 {0.851429, 1.10197, 1.63986, 2.73796, 5.07667},
 {0.859588, 1.14342, 1.76886, 3.10232, 6.10382}}
```

```
TableForm[Zclean]
```

0.800405	0.814215	0.851429	0.911003	0.993592
0.803316	0.843438	0.928855	1.06162	1.25205
0.8082	0.876311	1.0127	1.22973	1.55822
0.814215	0.911003	1.10197	1.41703	1.92197
0.820931	0.947004	1.19682	1.62602	2.35478

```
Tz = TableForm[Zclean, TableHeadings →
   {{"1", "2", "3", "4", "5"}, {"0.10", "0.20", "0.30", "0.40", "0.50"}}]
```

	0.10	0.20	0.30	0.40	0.50
1	0.800405	0.814215	0.851429	0.911003	0.993592
2	0.803316	0.843438	0.928855	1.06162	1.25205
3	0.8082	0.876311	1.0127	1.22973	1.55822
4	0.814215	0.911003	1.10197	1.41703	1.92197
5	0.820931	0.947004	1.19682	1.62602	2.35478

```
ListPointPlot3D[{
  {0.1`, 1, 0.8004050858331518`},
  {0.1`, 2, 0.8033159269246134`},
  {0.1`, 3, 0.8082003269581902`},
  {0.1`, 4, 0.8142148968730688`},
  {0.1`, 5, 0.8209312885048027`},
  {0.2`, 1, 0.8142148968730688`},
  {0.2`, 2, 0.8434380185848872`},
  {0.2`, 3, 0.8763111361689002`},
  {0.2`, 4, 0.9110027083001335`},
  {0.2`, 5, 0.9470039553121227`},
  {0.3`, 1, 0.8514294939505`},
  {0.3`, 2, 0.9288546334401951`},
  {0.3`, 3, 1.012697378136277`},
  {0.3`, 4, 1.1019720124348444`},
  {0.3`, 5, 1.1968179440522906`},
  {0.4`, 1, 0.9110027083001335`},
  {0.4`, 2, 1.0616232294135968`},
  {0.4`, 3, 1.2297304536779954`},
  {0.4`, 4, 1.4170294908197523`},
  {0.4`, 5, 1.6260153066471248`},
  {0.5`, 1, 0.9935921713955802`},
  {0.5`, 2, 1.2520452707411047`},
  {0.5`, 3, 1.5582156054709906`},
  {0.5`, 4, 1.9219701289949322`},
  {0.5`, 5, 2.3547750318723626`}},
 PlotStyle → {PointSize[0.03`]}, Axes → {True, True, True},
 PlotRange → {{0, 0.6}, {0, 6}, {0.7`, 2.5}}, BoxRatios → {5,
   5, 8}, AxesLabel → {Style["U", FontSize → 16, Bold],
   Style["T", FontSize → 16, Bold], Style["CSV", FontSize → 16, Bold]},
 BoxStyle → Directive[Orange, Dashed], PlotRange → All,
 ColorFunction → "ThermometerColors", AxesStyle → Directive[Orange, Dashed]]
```

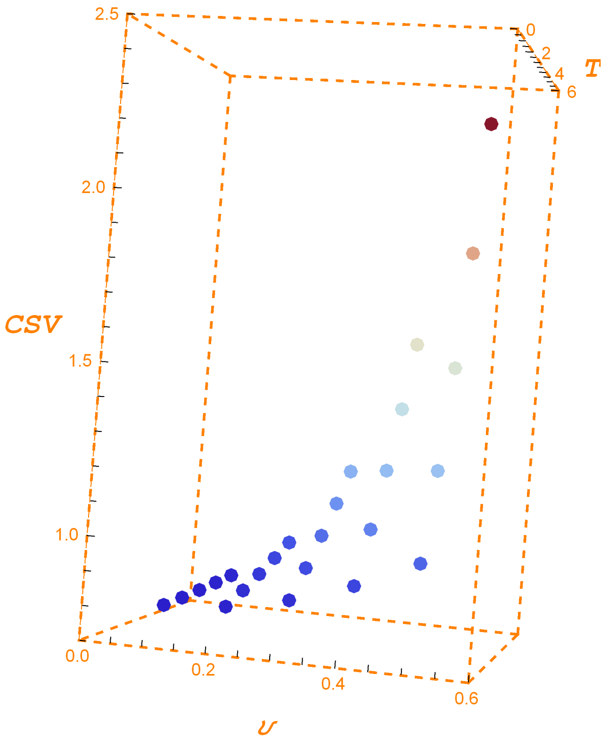
2.5
2.0
1.5
1.0
0.0
CSV
0.2
0.4
0.6
υ
0
2
4
6
T
