# Supplementary material for: Corporate Social Responsibility: A Real Options Approach to the Challenge of Financial Sustainability
Source: PLoS One. 2015 May 4;10(5):e0125972. doi: 10.1371/journal.pone.0125972 (PMC4418608; doi:10.1371/journal.pone.0125972)

S9 Fig: Mathematica code for Figure 12

call CSR option

```
ndist = NormalDistribution[0, 1]
```

```
NormalDistribution[0, 1]
```

```
Clear[K, A, σ, v, u, T, r, a, c, npv]
```

```
a = npv + 1
```

```
1 + npv
```

$$d1 = \frac{Log[a] + \left(r + \frac{\sigma^2}{2}\right) * T}{\sigma * \sqrt{T}}$$

$$\frac{T\left(r + \frac{\sigma^2}{2}\right) + Log[1 + npv]}{\sqrt{T}\ \sigma}$$

$$d2 = d1 - \sigma * \sqrt{T}$$

$$-\sqrt{T}\ \sigma + \frac{T\left(r + \frac{\sigma^2}{2}\right) + Log[1 + npv]}{\sqrt{T}\ \sigma}$$

```
oprplot = a * CDF[ndist, d1] - Exp[-r * T] CDF[ndist, d2]
```

$$\frac{1}{2}(1 + npv)\ Erfc\left[-\frac{T\left(r + \frac{\sigma^2}{2}\right) + Log[1 + npv]}{\sqrt{2}\sqrt{T}\ \sigma}\right] - \frac{1}{2}e^{-r\,T}\ Erfc\left[\frac{\sqrt{T}\ \sigma - \frac{T\left(r+\frac{\sigma^2}{2}\right)+Log[1+npv]}{\sqrt{T}\ \sigma}}{\sqrt{2}}\right]$$

```
r = 0.02
```

```
0.02
```

```
T = 5
```

```
5
```

```
σ = 0.20
```

```
0.2
```

```
npv = 0
```

```
0
```

```
N[oprplot]
```

```
0.220221
```

```
Clear[σ, npv]
```

```
y = Plot3D[{oprplot}, {σ, 0.01, 0.4},
   {npv, -1, 1}, AxesLabel → {Style["σ", FontSize → 20, Bold],
     Style["NPVR", FontSize → 18, Bold], Style["OPR", FontSize → 18, Bold]},
   PlotPoints → 50, PlotRange → Automatic, BaselinePosition → Center,
   ImageSize → {500, 400}, ColorFunction → "NeonColors",
   BoxStyle → Directive[Orange, Thick], AxesStyle → Directive[Orange]]
```

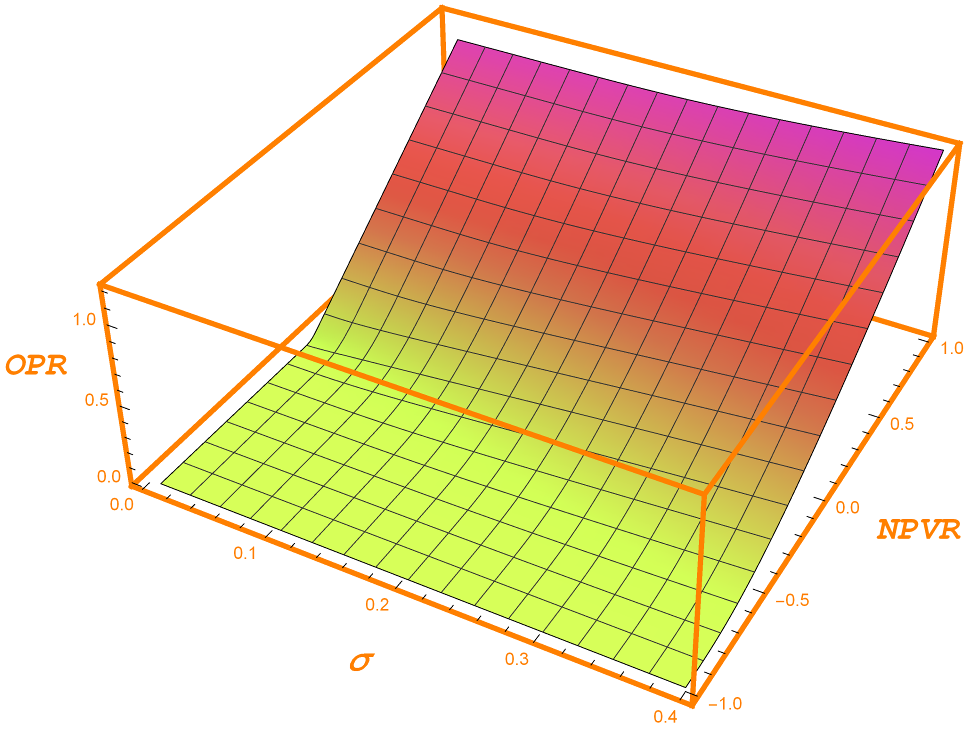


```
Clear[plotrect]

plotrect = RectangleChart3D[{{2, 0, 2}, {2, 0, 2}}, ColorFunction → "MintColors"]
```

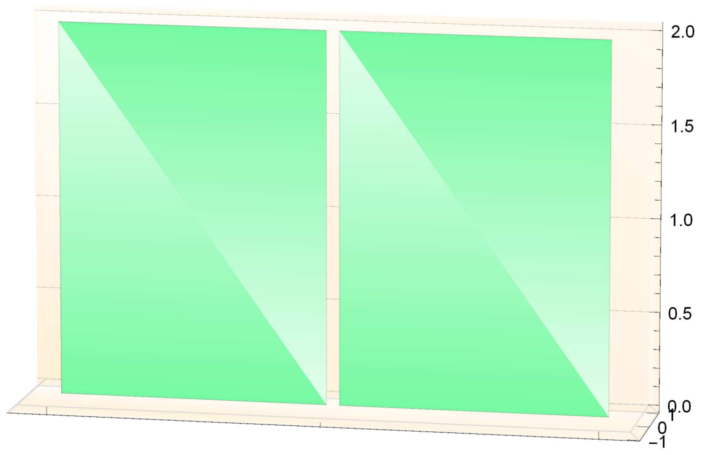

```
Arrow1 = Graphics3D[{White, Thickness[0.015],
   Arrowheads[.07], Arrow[{{0.15, -0.4, 0.22022086797273177}, {0.2`, 0,
      0.22022086797273177}}]}]
```

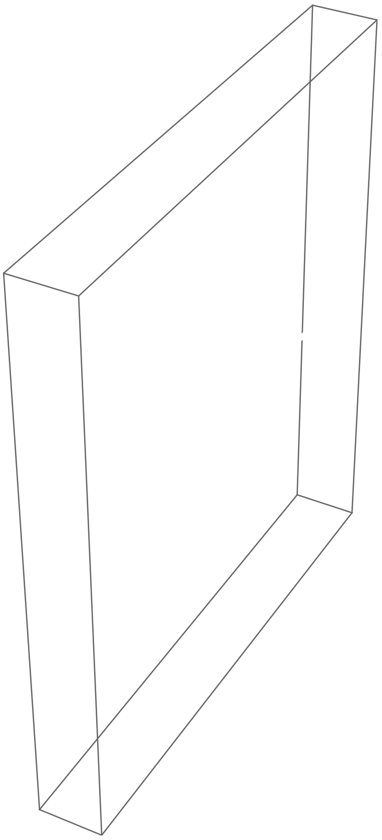

```
vcr1 = Show[y, Arrow1, plotrect]
```

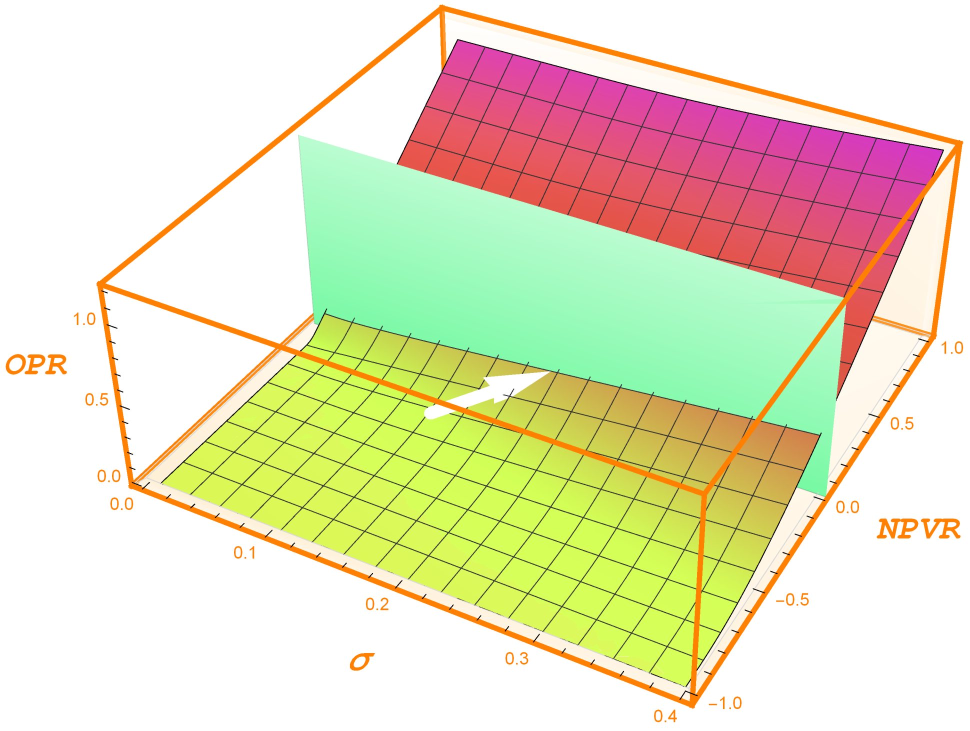

CSR call option

call CSR option

```
ndist = NormalDistribution[0, 1]
```

NormalDistribution[0, 1]

```
Clear[T, σ, υ, u, T, c, snpv]
```

$$dx1 = \frac{\text{Log}[\text{snpv}+1] + \left(\frac{\upsilon^2}{2}\right) * T}{\upsilon * \sqrt{T}}$$

$$\frac{\frac{T\,\upsilon^2}{2} + \text{Log}[1+\text{snpv}]}{\sqrt{T}\ \upsilon}$$

$$dx2 = dx1 - \upsilon * \sqrt{T}$$

$$-\sqrt{T}\ \upsilon + \frac{\frac{T\,\upsilon^2}{2} + \text{Log}[1+\text{snpv}]}{\sqrt{T}\ \upsilon}$$

$$\text{osrplot} = \text{CDF}[\text{ndist}, dx1] - \left(\frac{1}{\text{snpv}+1}\right) * \text{CDF}[\text{ndist}, dx2]$$

$$\frac{1}{2}\,\text{Erfc}\left[-\frac{\frac{T\,\upsilon^2}{2} + \text{Log}[1+\text{snpv}]}{\sqrt{2}\ \sqrt{T}\ \upsilon}\right] - \frac{\text{Erfc}\left[\frac{\sqrt{T}\ \upsilon - \frac{\frac{T\,\upsilon^2}{2}+\text{Log}[1+\text{snpv}]}{\sqrt{T}\ \upsilon}}{\sqrt{2}}\right]}{2\ (1+\text{snpv})}$$

```
FullSimplify[%]
```

$$\frac{\text{snpv} + \text{Erf}\left[\frac{T\,\upsilon^2 - 2\,\text{Log}[1+\text{snpv}]}{2\sqrt{2}\,\sqrt{T}\,\upsilon}\right] + (1+\text{snpv})\,\text{Erf}\left[\frac{\frac{T\,\upsilon^2}{2} + \text{Log}[1+\text{snpv}]}{\sqrt{2}\,\sqrt{T}\,\upsilon}\right]}{2\,(1+\text{snpv})}$$

```
T = 5
5

υ = 0.20
0.2

snpv = 0
0

N[osrplot]
0.176937

Clear[snpv, υ]

ySbs = Plot3D[{osrplot, GrayLevel[s]},
  {υ, 0.01, 0.4}, {snpv, -0.99, 1}, AxesLabel →
   {Style["σ", FontSize → 20, Bold], Style["SNPVR", FontSize → 18, Bold],
    Style["OSR", FontSize → 18, Bold]}, ColorFunction → "TemperatureMap",
  BoxStyle → Directive[Orange, Thick], AxesStyle → Directive[Orange],
  PlotPoints → 50, ImageSize → {500, 400}]
```

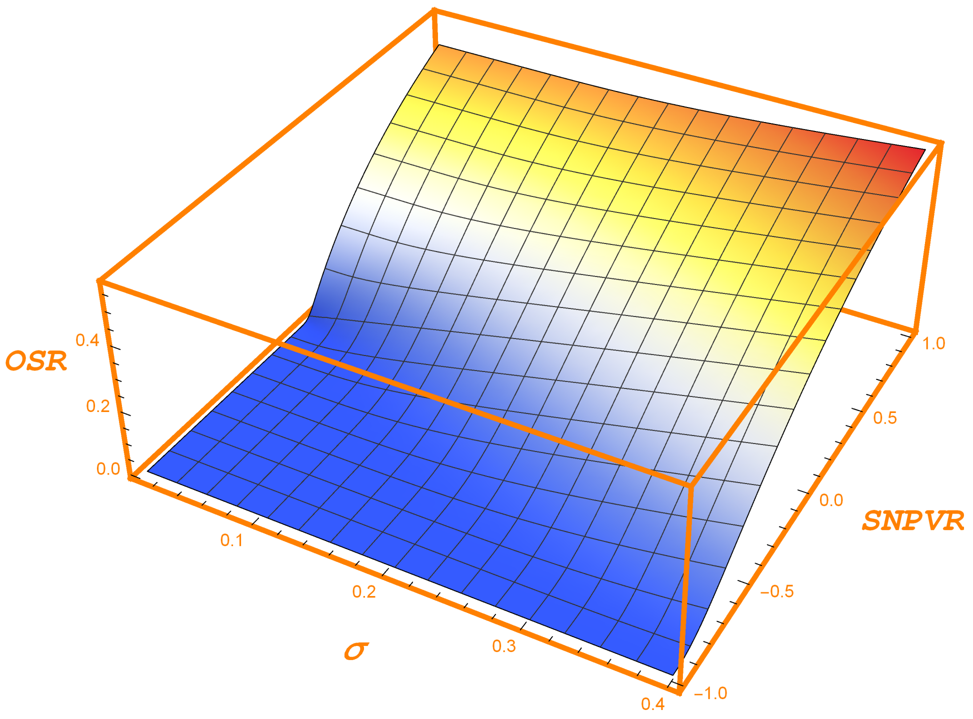

```
PlotArrow2 = Graphics3D[{White, Thickness[0.0145], Arrowheads[0.05],
   Arrow[{{0.14, -0.4, 0.17693672624187856`}, {0.2`, 0, 0.17693672624187856`}}]}]
```

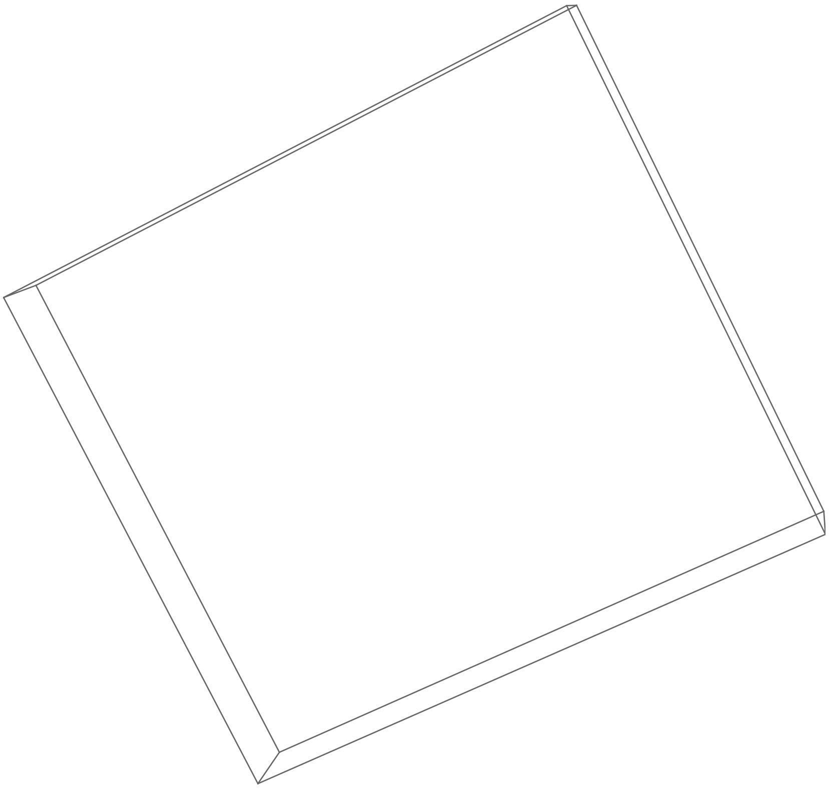

```
plotrect = RectangleChart3D[{{2, 0, 2}, {2, 0, 2}}, ColorFunction → "Aquamarine"]
```

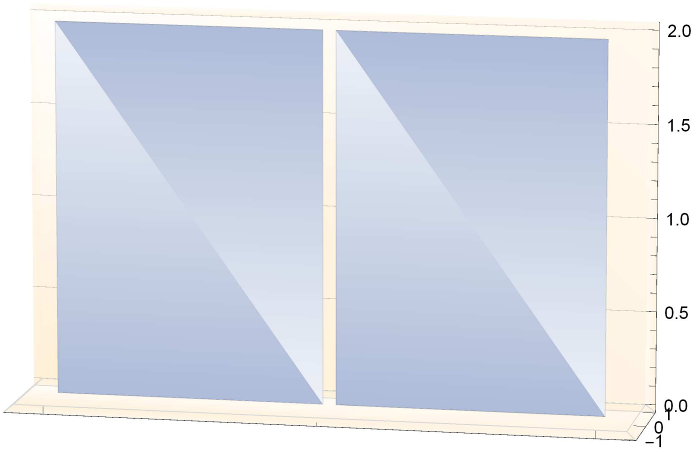

```
vcr2 = Show[ySbs, plotrect, PlotArrow2]
```

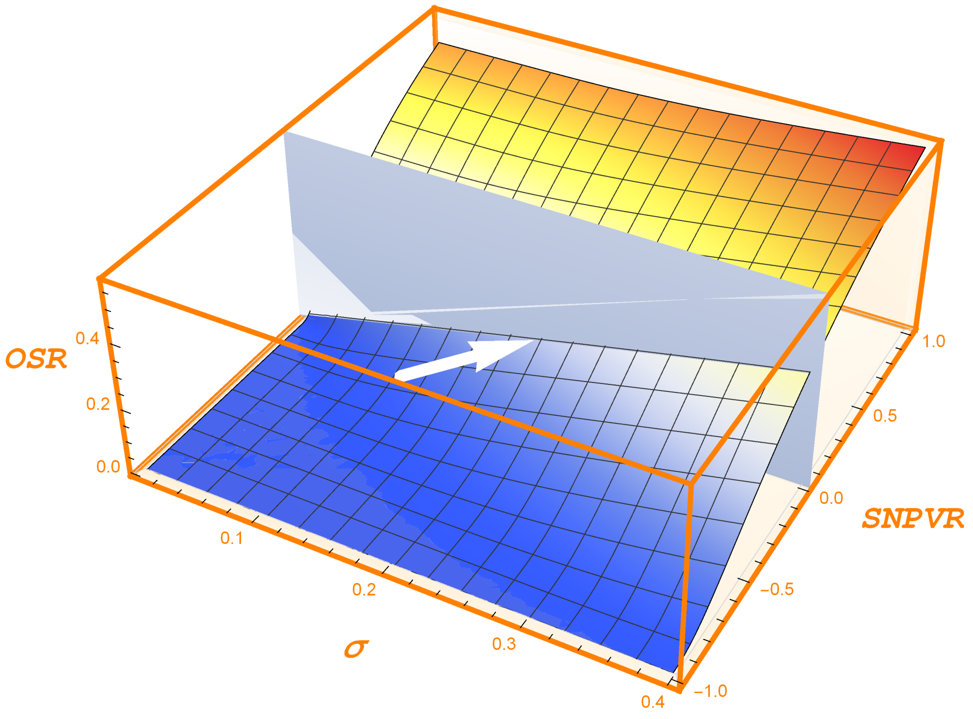

Supplement: S9 Fig — (PDF) [file pone.0125972.s009.pdf]
