# Supplementary material for: Corporate Social Responsibility: A Real Options Approach to the Challenge of Financial Sustainability
Source: PLoS One. 2015 May 4;10(5):e0125972. doi: 10.1371/journal.pone.0125972 (PMC4418608; doi:10.1371/journal.pone.0125972)
Supplement: S1 Table — (PDF) [file pone.0125972.s010.pdf]

S1 Table: *Mathematica* code for Table 1

```
ndist = NormalDistribution[0, 1]
```

NormalDistribution[0, 1]

```
Clear[K, A, σ, v, u, T, r, a, c]
```

$$d1 = \frac{Log[a] + \left(r + \frac{\sigma^2}{2}\right) * T}{\sigma * \sqrt{T}}$$

$$\frac{T\left(r + \frac{\sigma^2}{2}\right) + Log[a]}{\sqrt{T}\,\sigma}$$

$$d2 = d1 - \sigma * \sqrt{T}$$

$$-\sqrt{T}\,\sigma + \frac{T\left(r + \frac{\sigma^2}{2}\right) + Log[a]}{\sqrt{T}\,\sigma}$$

```
OPR = a * CDF[ndist, d1] - Exp[-r * T] * CDF[ndist, d2]
```

$$\frac{1}{2}\,a\,Erfc\left[-\frac{T\left(r + \frac{\sigma^2}{2}\right) + Log[a]}{\sqrt{2}\,\sqrt{T}\,\sigma}\right] - \frac{1}{2}\,e^{-r\,T}\,Erfc\left[\frac{\sqrt{T}\,\sigma - \frac{T\left(r+\frac{\sigma^2}{2}\right)+Log[a]}{\sqrt{T}\,\sigma}}{\sqrt{2}}\right]$$

```
r = 0.02
```

0.02

```
σ = 0.10
```

0.1

```
Clear[tableOPR010]
```

```
tableOPR010 =
 Table[OPR, {T, {3, 5, 10, 15, 20, 25, 50}}, {a, {0.5, 0.75, 1, 1.25, 1.5}}]
```

{{3.69177×10^{-6}, 0.00640864, 0.10009, 0.312247, 0.558462},
{0.000184998, 0.0205712, 0.140663, 0.353016, 0.596227},
{0.00512366, 0.0679065, 0.226724, 0.444577, 0.684962},
{0.0188537, 0.119105, 0.300623, 0.524201, 0.764623},
{0.039314, 0.169365, 0.366266, 0.594644, 0.835944},
{0.0638088, 0.217227, 0.425276, 0.657556, 0.899936},
{0.196527, 0.411447, 0.646701, 0.889898, 1.13651}}

```
Export["OPR010.xls", tableOPR010]
```

OPR010.xls

```
σ = 0.15
```

0.15

```
Clear[tableOPR015]
```

```
tableOPR015 =
 Table[OPR, {T, {3, 5, 10, 15, 20, 25, 50}}, {a, {0.5, 0.75, 1, 1.25, 1.5}}]
{{0.000430591, 0.022779, 0.132114, 0.327863, 0.562709},
 {0.00344861, 0.0493787, 0.179967, 0.376731, 0.606319},
 {0.0230141, 0.115141, 0.275713, 0.479335, 0.705945},
 {0.0513167, 0.174989, 0.353514, 0.563846, 0.791604},
 {0.0823432, 0.228897, 0.420113, 0.63615, 0.865963},
 {0.113545, 0.277513, 0.478365, 0.699186, 0.931179},
 {0.247988, 0.459374, 0.686841, 0.922558, 1.16297}}

Export["OPR015.xls", tableOPR015]
OPR015.xls

σ = 0.20
0.2

Clear[tableOPR020]

tableOPR020 =
 Table[OPR, {T, {3, 5, 10, 15, 20, 25, 50}}, {a, {0.5, 0.75, 1, 1.25, 1.5}}]
{{0.00312746, 0.044322, 0.1646, 0.351661, 0.575128},
 {0.0127233, 0.0814324, 0.220221, 0.409633, 0.628276},
 {0.0489545, 0.162096, 0.32709, 0.524936, 0.74262},
 {0.088698, 0.229332, 0.410302, 0.615658, 0.835969},
 {0.126852, 0.286847, 0.479293, 0.690896, 0.914336},
 {0.162202, 0.336796, 0.538079, 0.754888, 0.981335},
 {0.296353, 0.51097, 0.737411, 0.97053, 1.20783}}

Export["OPR020.xls", tableOPR020]
OPR020.xls

σ = 0.30
0.3

Clear[tableOPR030]

tableOPR030 =
 Table[OPR, {T, {3, 5, 10, 15, 20, 25, 50}}, {a, {0.5, 0.75, 1, 1.25, 1.5}}]
{{0.0188225, 0.0934465, 0.229432, 0.410321, 0.619464},
 {0.0450733, 0.148041, 0.300436, 0.486296, 0.693799},
 {0.109876, 0.252858, 0.429101, 0.626519, 0.837924},
 {0.165336, 0.331636, 0.522653, 0.729078, 0.945752},
 {0.211947, 0.394323, 0.59596, 0.809474, 1.03092},
 {0.251291, 0.445623, 0.655383, 0.874597, 1.10014},
 {0.376823, 0.602851, 0.835033, 1.07094, 1.30934}}

Export["OPR030.xls", tableOPR030]
OPR030.xls
```

```
σ = 0.50
0.5

Clear[tableOPR050]

tableOPR050 =
 Table[OPR, {T, {3, 5, 10, 15, 20, 25, 50}}, {a, {0.5, 0.75, 1, 1.25, 1.5}}]
{{0.0763357, 0.196578, 0.35509, 0.539344, 0.741089},
 {0.130853, 0.277704, 0.452715, 0.646112, 0.852132},
 {0.231322, 0.41391, 0.613044, 0.822737, 1.03974},
 {0.299515, 0.501844, 0.715215, 0.935577, 1.16079},
 {0.348156, 0.563263, 0.786151, 1.01395, 1.24515},
 {0.383846, 0.607794, 0.837396, 1.07056, 1.30619},
 {0.467022, 0.710081, 0.954544, 1.19989, 1.44584}}

Export["OPR050.xls", tableOPR050]
OPR050.xls
```
