# Supplementary material for: Corporate Social Responsibility: A Real Options Approach to the Challenge of Financial Sustainability
Source: PLoS One. 2015 May 4;10(5):e0125972. doi: 10.1371/journal.pone.0125972 (PMC4418608; doi:10.1371/journal.pone.0125972)
Supplement: S2 Table — (PDF) [file pone.0125972.s011.pdf]

S2Table: *Mathematica* code for Table 2

```
ndist = NormalDistribution[0, 1]
```

NormalDistribution[0, 1]

```
Clear[K, A, σ, v, u, T, r, a, c]
```

$$d1 = \frac{\text{Log}[a] + \left(r + \frac{\sigma^2}{2}\right) * T}{\sigma * \sqrt{T}}$$

$$\frac{T\left(r + \frac{\sigma^2}{2}\right) + \text{Log}[a]}{\sqrt{T}\ \sigma}$$

$$d2 = d1 - \sigma * \sqrt{T}$$

$$-\sqrt{T}\ \sigma + \frac{T\left(r + \frac{\sigma^2}{2}\right) + \text{Log}[a]}{\sqrt{T}\ \sigma}$$

```
newprojectratio = CDF[ndist, d1] - (1/a) * Exp[-r * T] CDF[ndist, d2]
```

$$\frac{1}{2}\,\text{Erfc}\left[-\frac{T\left(r + \frac{\sigma^2}{2}\right) + \text{Log}[a]}{\sqrt{2}\ \sqrt{T}\ \sigma}\right] - \frac{e^{-r\,T}\,\text{Erfc}\left[\frac{\sqrt{T}\ \sigma - \frac{T\left(r+\frac{\sigma^2}{2}\right)+\text{Log}[a]}{\sqrt{T}\ \sigma}}{\sqrt{2}}\right]}{2\,a}$$

```
r = 0.02
```

0.02

```
σ = 0.10
```

0.1

```
Clear[tableOP]
```

```
NPR010 = Table[newprojectratio,
   {T, {3, 5, 10, 15, 20, 25, 50}}, {a, {0.5, 0.75, 1, 1.25, 1.5}}]
```

{{7.38354×10^{-6}, 0.00854485, 0.10009, 0.249797, 0.372308},
{0.000369997, 0.0274283, 0.140663, 0.282413, 0.397485},
{0.0102473, 0.0905419, 0.226724, 0.355661, 0.456641},
{0.0377074, 0.158807, 0.300623, 0.419361, 0.509749},
{0.0786281, 0.22582, 0.366266, 0.475715, 0.557296},
{0.127618, 0.289635, 0.425276, 0.526045, 0.599957},
{0.393054, 0.548596, 0.646701, 0.711918, 0.757673}}

```
Export["NPR010.xls", NPR010]
```

NPR010.xls

```
σ = 0.15
```

0.15

```
Clear[tableOP]
```

```
NPR015 = Table[newprojectratio,
   {T, {3, 5, 10, 15, 20, 25, 50}}, {a, {0.5, 0.75, 1, 1.25, 1.5}}]
{{0.000861182, 0.030372, 0.132114, 0.26229, 0.375139},
 {0.00689723, 0.0658383, 0.179967, 0.301385, 0.404212},
 {0.0460282, 0.153521, 0.275713, 0.383468, 0.47063},
 {0.102633, 0.233318, 0.353514, 0.451077, 0.527736},
 {0.164686, 0.305196, 0.420113, 0.50892, 0.577309},
 {0.22709, 0.370017, 0.478365, 0.559349, 0.620786},
 {0.495976, 0.612499, 0.686841, 0.738047, 0.77531}}

Export["NPR015.xls", NPR015]
NPR015.xls

σ = 0.20
0.2

Clear[tableOP]

NPR020 = Table[newprojectratio,
   {T, {3, 5, 10, 15, 20, 25, 50}}, {a, {0.5, 0.75, 1, 1.25, 1.5}}]
{{0.00625492, 0.059096, 0.1646, 0.281329, 0.383418},
 {0.0254466, 0.108577, 0.220221, 0.327706, 0.418851},
 {0.0979091, 0.216128, 0.32709, 0.419949, 0.49508},
 {0.177396, 0.305777, 0.410302, 0.492527, 0.557312},
 {0.253704, 0.382462, 0.479293, 0.552717, 0.609557},
 {0.324404, 0.449062, 0.538079, 0.60391, 0.654223},
 {0.592706, 0.681293, 0.737411, 0.776424, 0.805222}}

Export["NPR020.xls", NPR020]
NPR020.xls

σ = 0.30
0.3

Clear[tableOP]

NPR030 = Table[newprojectratio,
   {T, {3, 5, 10, 15, 20, 25, 50}}, {a, {0.5, 0.75, 1, 1.25, 1.5}}]
{{0.0376451, 0.124595, 0.229432, 0.328257, 0.412976},
 {0.0901466, 0.197388, 0.300436, 0.389037, 0.462533},
 {0.219751, 0.337144, 0.429101, 0.501215, 0.558616},
 {0.330673, 0.442182, 0.522653, 0.583262, 0.630501},
 {0.423895, 0.525765, 0.59596, 0.647579, 0.687279},
 {0.502582, 0.594164, 0.655383, 0.699677, 0.733423},
 {0.753646, 0.803802, 0.835033, 0.85675, 0.872895}}

Export["NPR030.xls", NPR030]
NPR030.xls
```

```
σ = 0.50
0.5

NPR050 = Table[newprojectratio,
  {T, {3, 5, 10, 15, 20, 25, 50}}, {a, {0.5, 0.75, 1, 1.25, 1.5}}]
{{0.152671, 0.262104, 0.35509, 0.431475, 0.494059},
 {0.261707, 0.370272, 0.452715, 0.51689, 0.568088},
 {0.462645, 0.55188, 0.613044, 0.658189, 0.693158},
 {0.59903, 0.669126, 0.715215, 0.748462, 0.77386},
 {0.696312, 0.751017, 0.786151, 0.811158, 0.830103},
 {0.767692, 0.810392, 0.837396, 0.856446, 0.870796},
 {0.934043, 0.946774, 0.954544, 0.95991, 0.963896}}

Export["NPR050.xls", NPR050]
NPR050.xls
```
