# Supplementary material for: Corporate Social Responsibility: A Real Options Approach to the Challenge of Financial Sustainability
Source: PLoS One. 2015 May 4;10(5):e0125972. doi: 10.1371/journal.pone.0125972 (PMC4418608; doi:10.1371/journal.pone.0125972)
Supplement: S3 Table — (PDF) [file pone.0125972.s012.pdf]

S3Table: *Mathematica* code forTable 3

```
Clear[A, K, σ, a, u, T, r, ck, z, d1, d2]
```

```
ndist = NormalDistribution[0, 1]
```

```
NormalDistribution[0, 1]
```

$$d1 = \frac{\mathrm{Log}[a] + \left(r + \frac{\sigma^2}{2}\right) * T}{\sigma * \sqrt{T}}$$

$$\frac{T\left(r + \frac{\sigma^2}{2}\right) + \mathrm{Log}[a]}{\sqrt{T}\ \sigma}$$

$$d2 = d1 - \sigma * \sqrt{T}$$

$$-\sqrt{T}\ \sigma + \frac{T\left(r + \frac{\sigma^2}{2}\right) + \mathrm{Log}[a]}{\sqrt{T}\ \sigma}$$

```
ck = a * CDF[ndist, d1] - Exp[-r * T] CDF[ndist, d2]
```

$$\frac{1}{2}\, a\, \mathrm{Erfc}\left[-\frac{T\left(r + \frac{\sigma^2}{2}\right) + \mathrm{Log}[a]}{\sqrt{2}\ \sqrt{T}\ \sigma}\right] - \frac{1}{2}\, e^{-r\,T}\, \mathrm{Erfc}\left[\frac{\sqrt{T}\ \sigma - \frac{T\left(r+\frac{\sigma^2}{2}\right)+\mathrm{Log}[a]}{\sqrt{T}\ \sigma}}{\sqrt{2}}\right]$$

```
T = 1
```

```
1
```

```
Clear[u]
```

```
u = ck - 0.25
```

$$-0.25 + \frac{1}{2}\, a\, \mathrm{Erfc}\left[-\frac{r + \frac{\sigma^2}{2} + \mathrm{Log}[a]}{\sqrt{2}\ \sigma}\right] - \frac{1}{2}\, e^{-r}\, \mathrm{Erfc}\left[\frac{\sigma - \frac{r+\frac{\sigma^2}{2}+\mathrm{Log}[a]}{\sigma}}{\sqrt{2}}\right]$$

```
z1 = Table[FindRoot[u, {a, 10}],
   {r, {0.02, 0.03, 0.05, 0.07, 0.09, 0.10}}, {σ, {0.10, 0.20, 0.30, 0.40, 0.5}}]
```

```
{{{a → 1.22976}, {a → 1.21441}, {a → 1.17705}, {a → 1.12654}, {a → 1.06954}},
 {{a → 1.22003}, {a → 1.20515}, {a → 1.16854}, {a → 1.11884}, {a → 1.06264}},
 {{a → 1.20087}, {a → 1.18689}, {a → 1.15173}, {a → 1.10362}, {a → 1.04899}},
 {{a → 1.18208}, {a → 1.16896}, {a → 1.13521}, {a → 1.08865}, {a → 1.03556}},
 {{a → 1.16366}, {a → 1.15135}, {a → 1.11898}, {a → 1.07393}, {a → 1.02233}},
 {{a → 1.15459}, {a → 1.14267}, {a → 1.11097}, {a → 1.06666}, {a → 1.0158}}}
```

```
T31 = a /. z1
```

```
{{1.22976, 1.21441, 1.17705, 1.12654, 1.06954},
 {1.22003, 1.20515, 1.16854, 1.11884, 1.06264},
 {1.20087, 1.18689, 1.15173, 1.10362, 1.04899},
 {1.18208, 1.16896, 1.13521, 1.08865, 1.03556},
 {1.16366, 1.15135, 1.11898, 1.07393, 1.02233},
 {1.15459, 1.14267, 1.11097, 1.06666, 1.0158}}
```

```
T3H1 = TableForm[T31, TableHeadings →
   {{0.01, 0.02, 0.03, 0.04, 0.05, 0.06, 0.07, 0.08, 0.09, 0.10}, None}]
```

0.01	1.22976	1.21441	1.17705	1.12654	1.06954
0.02	1.22003	1.20515	1.16854	1.11884	1.06264
0.03	1.20087	1.18689	1.15173	1.10362	1.04899
0.04	1.18208	1.16896	1.13521	1.08865	1.03556
0.05	1.16366	1.15135	1.11898	1.07393	1.02233
0.06	1.15459	1.14267	1.11097	1.06666	1.0158

```
Export["Table3GPVT1.xls", T3H1]
```

```
Table3GPVT1.xls
```

```
T = 2
```

```
2
```

```
Clear[u]
```

```
u = ck - 0.25
```

$$-0.25 + \frac{1}{2}\, a\, \mathrm{Erfc}\left[-\frac{2\left(r + \frac{\sigma^2}{2}\right) + \mathrm{Log}[a]}{2\,\sigma}\right] - \frac{1}{2}\, e^{-2\,r}\, \mathrm{Erfc}\left[\frac{\sqrt{2}\,\sigma - \frac{2\left(r+\frac{\sigma^2}{2}\right)+\mathrm{Log}[a]}{\sqrt{2}\,\sigma}}{\sqrt{2}}\right]$$

```
z2 = Table[FindRoot[u, {a, 10}],
  {r, {0.02, 0.03, 0.05, 0.07, 0.09, 0.10}}, {σ, {0.10, 0.20, 0.30, 0.40, 0.5}}]
```

```
{{{a → 1.20738}, {a → 1.16741}, {a → 1.09816}, {a → 1.0179}, {a → 0.936251}},
 {{a → 1.18865}, {a → 1.15048}, {a → 1.08345}, {a → 1.00533}, {a → 0.925611}},
 {{a → 1.15226}, {a → 1.11751}, {a → 1.05474}, {a → 0.980769}, {a → 0.90479}},
 {{a → 1.11724}, {a → 1.08568}, {a → 1.02696}, {a → 0.956951}, {a → 0.884564}},
 {{a → 1.08354}, {a → 1.05495}, {a → 1.00007}, {a → 0.933855}, {a → 0.864916}},
 {{a → 1.06717}, {a → 1.03998}, {a → 0.986958}, {a → 0.92257}, {a → 0.855304}}}
```

```
T32 = a /. z2
```

```
{{1.20738, 1.16741, 1.09816, 1.0179, 0.936251},
 {1.18865, 1.15048, 1.08345, 1.00533, 0.925611},
 {1.15226, 1.11751, 1.05474, 0.980769, 0.90479},
 {1.11724, 1.08568, 1.02696, 0.956951, 0.884564},
 {1.08354, 1.05495, 1.00007, 0.933855, 0.864916},
 {1.06717, 1.03998, 0.986958, 0.92257, 0.855304}}
```

```
T3H2 = TableForm[T32, TableHeadings →
   {{0.01, 0.02, 0.03, 0.04, 0.05, 0.06, 0.07, 0.08, 0.09, 0.10}, None}]
```

0.01	1.20738	1.16741	1.09816	1.0179	0.936251
0.02	1.18865	1.15048	1.08345	1.00533	0.925611
0.03	1.15226	1.11751	1.05474	0.980769	0.90479
0.04	1.11724	1.08568	1.02696	0.956951	0.884564
0.05	1.08354	1.05495	1.00007	0.933855	0.864916
0.06	1.06717	1.03998	0.986958	0.92257	0.855304

```
Export["Table3GPVT2.xls", T3H2]
```

Table3GPVT2.xls

```
Clear[u]
```

```
u = ck - 0.25
```

$$-0.25 + \frac{1}{2} a\,\text{Erfc}\left[-\frac{2\left(r+\frac{\sigma^2}{2}\right)+\text{Log}[a]}{2\,\sigma}\right] - \frac{1}{2} e^{-2r}\,\text{Erfc}\left[\frac{\sqrt{2}\,\sigma - \frac{2\left(r+\frac{\sigma^2}{2}\right)+\text{Log}[a]}{\sqrt{2}\,\sigma}}{\sqrt{2}}\right]$$

```
z3 = Table[FindRoot[u, {a, 10}],
  {r, {0.02, 0.03, 0.05, 0.07, 0.09, 0.10}}, {σ, {0.10, 0.20, 0.30, 0.40, 0.5}}]
```

{{{a → 1.20738}, {a → 1.16741}, {a → 1.09816}, {a → 1.0179}, {a → 0.936251}},
 {{a → 1.18865}, {a → 1.15048}, {a → 1.08345}, {a → 1.00533}, {a → 0.925611}},
 {{a → 1.15226}, {a → 1.11751}, {a → 1.05474}, {a → 0.980769}, {a → 0.90479}},
 {{a → 1.11724}, {a → 1.08568}, {a → 1.02696}, {a → 0.956951}, {a → 0.884564}},
 {{a → 1.08354}, {a → 1.05495}, {a → 1.00007}, {a → 0.933855}, {a → 0.864916}},
 {{a → 1.06717}, {a → 1.03998}, {a → 0.986958}, {a → 0.92257}, {a → 0.855304}}}

```
T33 = a /. z3
```

{{1.20738, 1.16741, 1.09816, 1.0179, 0.936251},
 {1.18865, 1.15048, 1.08345, 1.00533, 0.925611},
 {1.15226, 1.11751, 1.05474, 0.980769, 0.90479},
 {1.11724, 1.08568, 1.02696, 0.956951, 0.884564},
 {1.08354, 1.05495, 1.00007, 0.933855, 0.864916},
 {1.06717, 1.03998, 0.986958, 0.92257, 0.855304}}

```
T3H3 = TableForm[T33, TableHeadings →
   {{0.01, 0.02, 0.03, 0.04, 0.05, 0.06, 0.07, 0.08, 0.09, 0.10}, None}]
```

0.01	1.20738	1.16741	1.09816	1.0179	0.936251
0.02	1.18865	1.15048	1.08345	1.00533	0.925611
0.03	1.15226	1.11751	1.05474	0.980769	0.90479
0.04	1.11724	1.08568	1.02696	0.956951	0.884564
0.05	1.08354	1.05495	1.00007	0.933855	0.864916
0.06	1.06717	1.03998	0.986958	0.92257	0.855304

```
Export["Table3GPVT3.xls", T3H3]
```

Table3GPVT3.xls

```
T = 4
```

4

```
Clear[u]

u = ck - 0.25
```

$$-0.25+\frac{1}{2}\,a\,\mathrm{Erfc}\Big[-\frac{4\left(r+\frac{\sigma^2}{2}\right)+\mathrm{Log}[a]}{2\sqrt{2}\,\sigma}\Big]-\frac{1}{2}\,e^{-4\,r}\,\mathrm{Erfc}\Big[\frac{2\,\sigma-\frac{4\left(r+\frac{\sigma^2}{2}\right)+\mathrm{Log}[a]}{2\,\sigma}}{\sqrt{2}}\Big]$$

```
z4 = Table[FindRoot[u, {a, 10}],
  {r, {0.02, 0.03, 0.05, 0.07, 0.09, 0.10}}, {σ, {0.10, 0.20, 0.30, 0.40, 0.5}}]

{{{a → 1.16011}, {a → 1.08126}, {a → 0.973999}, {a → 0.865443}, {a → 0.766023}},
 {{a → 1.12555}, {a → 1.0523}, {a → 0.950742}, {a → 0.847076}, {a → 0.75162}},
 {{a → 1.06013}, {a → 0.997182}, {a → 0.906291}, {a → 0.811849}, {a → 0.723912}},
 {{a → 0.99939}, {a → 0.945599}, {a → 0.864456}, {a → 0.778539}, {a → 0.697605}},
 {{a → 0.94301}, {a → 0.897333}, {a → 0.825086}, {a → 0.747042}, {a → 0.67263}},
 {{a → 0.916364}, {a → 0.87438}, {a → 0.80628}, {a → 0.731944}, {a → 0.66062}}}

T34 = a /. z4

{{1.16011, 1.08126, 0.973999, 0.865443, 0.766023},
 {1.12555, 1.0523, 0.950742, 0.847076, 0.75162},
 {1.06013, 0.997182, 0.906291, 0.811849, 0.723912},
 {0.99939, 0.945599, 0.864456, 0.778539, 0.697605},
 {0.94301, 0.897333, 0.825086, 0.747042, 0.67263},
 {0.916364, 0.87438, 0.80628, 0.731944, 0.66062}}

T3H4 = TableForm[T34, TableHeadings →
   {{0.01, 0.02, 0.03, 0.04, 0.05, 0.06, 0.07, 0.08, 0.09, 0.10}, None}]
```

0.01	1.16011	1.08126	0.973999	0.865443	0.766023
0.02	1.12555	1.0523	0.950742	0.847076	0.75162
0.03	1.06013	0.997182	0.906291	0.811849	0.723912
0.04	0.99939	0.945599	0.864456	0.778539	0.697605
0.05	0.94301	0.897333	0.825086	0.747042	0.67263
0.06	0.916364	0.87438	0.80628	0.731944	0.66062

```
Export["Table3GPVT4.xls", T3H4]

Table3GPVT4.xls

T = 5

5

Clear[u]

u = ck - 0.25
```

$$-0.25+\frac{1}{2}\,a\,\mathrm{Erfc}\Big[-\frac{5\left(r+\frac{\sigma^2}{2}\right)+\mathrm{Log}[a]}{\sqrt{10}\,\sigma}\Big]-\frac{1}{2}\,e^{-5\,r}\,\mathrm{Erfc}\Big[\frac{\sqrt{5}\,\sigma-\frac{5\left(r+\frac{\sigma^2}{2}\right)+\mathrm{Log}[a]}{\sqrt{5}\,\sigma}}{\sqrt{2}}\Big]$$

```
z5 = Table[FindRoot[u, {a, 10}],
  {r, {0.02, 0.03, 0.05, 0.07, 0.09, 0.10}}, {σ, {0.10, 0.20, 0.30, 0.40, 0.5}}]

{{{a → 1.13674}, {a → 1.04319}, {a → 0.924159}, {a → 0.80881}, {a → 0.706875}},
 {{a → 1.0952}, {a → 1.00942}, {a → 0.897873}, {a → 0.788673}, {a → 0.691537}},
 {{a → 1.01759}, {a → 0.945857}, {a → 0.848117}, {a → 0.75038}, {a → 0.662256}},
 {{a → 0.946765}, {a → 0.887251}, {a → 0.801895}, {a → 0.714586}, {a → 0.634741}},
 {{a → 0.882172}, {a → 0.833225}, {a → 0.758959}, {a → 0.68113}, {a → 0.608888}},
 {{a → 0.852051}, {a → 0.807821}, {a → 0.738651}, {a → 0.665231}, {a → 0.596553}}}

T35 = a /. z5
{{1.13674, 1.04319, 0.924159, 0.80881, 0.706875},
 {1.0952, 1.00942, 0.897873, 0.788673, 0.691537},
 {1.01759, 0.945857, 0.848117, 0.75038, 0.662256},
 {0.946765, 0.887251, 0.801895, 0.714586, 0.634741},
 {0.882172, 0.833225, 0.758959, 0.68113, 0.608888},
 {0.852051, 0.807821, 0.738651, 0.665231, 0.596553}}

T3H5 = TableForm[T35, TableHeadings →
   {{0.01, 0.02, 0.03, 0.04, 0.05, 0.06, 0.07, 0.08, 0.09, 0.10}, None}]
```

0.01	1.13674	1.04319	0.924159	0.80881	0.706875
0.02	1.0952	1.00942	0.897873	0.788673	0.691537
0.03	1.01759	0.945857	0.848117	0.75038	0.662256
0.04	0.946765	0.887251	0.801895	0.714586	0.634741
0.05	0.882172	0.833225	0.758959	0.68113	0.608888
0.06	0.852051	0.807821	0.738651	0.665231	0.596553

```
Export["Table3GPVT5.xls", T3H5]
Table3GPVT5.xls
```
