# Supplementary material for: Corporate Social Responsibility: A Real Options Approach to the Challenge of Financial Sustainability
Source: PLoS One. 2015 May 4;10(5):e0125972. doi: 10.1371/journal.pone.0125972 (PMC4418608; doi:10.1371/journal.pone.0125972)
Supplement: S4 Table — (PDF) [file pone.0125972.s013.pdf]

S4 Table: *Mathematica* code for Table 4

```
Clear[A, K, a, d1, d2, ct, σ, v, y, T, r, ϖ, tabley]
```

```
ndist = NormalDistribution[0, 1]
```

```
NormalDistribution[0, 1]
```

$$d1 = \frac{Log[a] + \left(r + \frac{\sigma^2}{2}\right) * T}{\sigma * \sqrt{T}}$$

$$\frac{T\left(r + \frac{\sigma^2}{2}\right) + Log[a]}{\sqrt{T}\ \sigma}$$

$$d2 = d1 - \sigma * \sqrt{T}$$

$$-\sqrt{T}\ \sigma + \frac{T\left(r + \frac{\sigma^2}{2}\right) + Log[a]}{\sqrt{T}\ \sigma}$$

```
ct = a * CDF[ndist, d1] - Exp[-r * T] CDF[ndist, d2]
```

$$\frac{1}{2}\, a\, Erfc\left[-\frac{T\left(r + \frac{\sigma^2}{2}\right) + Log[a]}{\sqrt{2}\ \sqrt{T}\ \sigma}\right] - \frac{1}{2}\, e^{-r\,T}\, Erfc\left[\frac{\sqrt{T}\ \sigma - \frac{T\left(r+\frac{\sigma^2}{2}\right)+Log[a]}{\sqrt{T}\ \sigma}}{\sqrt{2}}\right]$$

```
Simplify[%]
```

$$\frac{1}{2}\left(-e^{-r\,T}\, Erfc\left[\frac{T\left(-2\,r + \sigma^2\right) - 2\, Log[a]}{2\sqrt{2}\ \sqrt{T}\ \sigma}\right] + a\, Erfc\left[-\frac{T\left(r + \frac{\sigma^2}{2}\right) + Log[a]}{\sqrt{2}\ \sqrt{T}\ \sigma}\right]\right)$$

```
a = 0.5
```

```
0.5
```

```
y = ct - 0.25
```

$$-0.25 + 0.25\, Erfc\left[-\frac{-0.693147 + T\left(r + \frac{\sigma^2}{2}\right)}{\sqrt{2}\ \sqrt{T}\ \sigma}\right] - \frac{1}{2}\, e^{-r\,T}\, Erfc\left[\frac{\sqrt{T}\ \sigma - \frac{-0.693147+T\left(r+\frac{\sigma^2}{2}\right)}{\sqrt{T}\ \sigma}}{\sqrt{2}}\right]$$

```
tabley05 = Table[FindRoot[y, {T, 60}],
   {r, {0.02, 0.03, 0.05, 0.07, 0.09, 0.10}}, {σ, {0.10, 0.20, 0.30, 0.40, 0.50}}]
```

```
{{{T → 61.5407}, {T → 39.8998}, {T → 24.8221}, {T → 16.2054}, {T → 11.2004}},
 {{T → 43.3122}, {T → 31.6593}, {T → 21.4134}, {T → 14.6901}, {T → 10.4576}},
 {{T → 27.0228}, {T → 22.314}, {T → 16.7675}, {T → 12.3642}, {T → 9.22862}},
 {{T → 19.5635}, {T → 17.1726}, {T → 13.7534}, {T → 10.664}, {T → 8.25379}},
 {{T → 15.3051}, {T → 13.9285}, {T → 11.6427}, {T → 9.36783}, {T → 7.46195}},
 {{T → 13.7975}, {T → 12.719}, {T → 10.8087}, {T → 8.82897}, {T → 7.11934}}}
```

```
T4a05 = T /. tabley05
```

```
{{61.5407, 39.8998, 24.8221, 16.2054, 11.2004},
 {43.3122, 31.6593, 21.4134, 14.6901, 10.4576},
 {27.0228, 22.314, 16.7675, 12.3642, 9.22862},
 {19.5635, 17.1726, 13.7534, 10.664, 8.25379},
 {15.3051, 13.9285, 11.6427, 9.36783, 7.46195},
 {13.7975, 12.719, 10.8087, 8.82897, 7.11934}}
```

```
T4TFa05 = TableForm[T4a05]
```

61.5407	39.8998	24.8221	16.2054	11.2004
43.3122	31.6593	21.4134	14.6901	10.4576
27.0228	22.314	16.7675	12.3642	9.22862
19.5635	17.1726	13.7534	10.664	8.25379
15.3051	13.9285	11.6427	9.36783	7.46195
13.7975	12.719	10.8087	8.82897	7.11934

```
T4Fa05hd = TableForm[T4a05,
  TableHeadings → {{"0.02", "0.03", "0.05", "0.07", ".09", "0.10"},
    {"0.10", "0.20", "0.30", "0.40", "0.50"}}]
```

	0.10	0.20	0.30	0.40	0.50
0.02	61.5407	39.8998	24.8221	16.2054	11.2004
0.03	43.3122	31.6593	21.4134	14.6901	10.4576
0.05	27.0228	22.314	16.7675	12.3642	9.22862
0.07	19.5635	17.1726	13.7534	10.664	8.25379
.09	15.3051	13.9285	11.6427	9.36783	7.46195
0.10	13.7975	12.719	10.8087	8.82897	7.11934

```
Export["Table4a05.xls", T4Fa05hd]
```

Table4a05.xls

```
a = 0.75
```

0.75

```
y = ct - 0.25
```

$$-0.25 + 0.375\,\mathrm{Erfc}\left[-\frac{-0.287682 + T\left(r + \frac{\sigma^2}{2}\right)}{\sqrt{2}\sqrt{T}\,\sigma}\right] - \frac{1}{2}\,e^{-r\,T}\,\mathrm{Erfc}\left[\frac{\sqrt{T}\,\sigma - \frac{-0.287682+T\left(r+\frac{\sigma^2}{2}\right)}{\sqrt{T}\,\sigma}}{\sqrt{2}}\right]$$

```
tabley075 = Table[FindRoot[y, {T, 20}],
  {r, {0.02, 0.03, 0.05, 0.07, 0.09, 0.10}}, {σ, {0.10, 0.20, 0.30, 0.40, 0.50}}]
```

```
{{{T → 28.6126}, {T → 16.7129}, {T → 9.84074}, {T → 6.24641}, {T → 4.25078}},
 {{T → 20.6202}, {T → 13.6885}, {T → 8.70908}, {T → 5.76986}, {T → 4.02431}},
 {{T → 13.1588}, {T → 10.0388}, {T → 7.0806}, {T → 5.00679}, {T → 3.63724}},
 {{T → 9.6267}, {T → 7.91407}, {T → 5.96368}, {T → 4.42221}, {T → 3.31841}},
 {{T → 7.57447}, {T → 6.52384}, {T → 5.14932}, {T → 3.95969}, {T → 3.05108}},
 {{T → 6.84147}, {T → 5.9948}, {T → 4.81952}, {T → 3.76278}, {T → 2.93295}}}
```

```
T4a075 = T /. tabley075
```

```
{{28.6126, 16.7129, 9.84074, 6.24641, 4.25078},
 {20.6202, 13.6885, 8.70908, 5.76986, 4.02431},
 {13.1588, 10.0388, 7.0806, 5.00679, 3.63724},
 {9.6267, 7.91407, 5.96368, 4.42221, 3.31841},
 {7.57447, 6.52384, 5.14932, 3.95969, 3.05108},
 {6.84147, 5.9948, 4.81952, 3.76278, 2.93295}}
```

```
T4TFa075 = TableForm[T4a075]
```

28.6126	16.7129	9.84074	6.24641	4.25078
20.6202	13.6885	8.70908	5.76986	4.02431
13.1588	10.0388	7.0806	5.00679	3.63724
9.6267	7.91407	5.96368	4.42221	3.31841
7.57447	6.52384	5.14932	3.95969	3.05108
6.84147	5.9948	4.81952	3.76278	2.93295

```
T4Fa075hd = TableForm[T4a075,
  TableHeadings → {{"0.02", "0.03", "0.05", "0.07", ".09", "0.10"},
    {"0.10", "0.20", "0.30", "0.40", "0.50"}}]
```

	0.10	0.20	0.30	0.40	0.50
0.02	28.6126	16.7129	9.84074	6.24641	4.25078
0.03	20.6202	13.6885	8.70908	5.76986	4.02431
0.05	13.1588	10.0388	7.0806	5.00679	3.63724
0.07	9.6267	7.91407	5.96368	4.42221	3.31841
.09	7.57447	6.52384	5.14932	3.95969	3.05108
0.10	6.84147	5.9948	4.81952	3.76278	2.93295

```
Export["Table4a075.xls", T4Fa075hd]
```

```
Table4a075.xls
```

```
a = 1
```

```
1
```

```
y = ct - 0.25
```

$$-0.25 + \frac{1}{2}\,\mathrm{Erfc}\left[-\frac{\sqrt{T}\,\left(r + \frac{\sigma^2}{2}\right)}{\sqrt{2}\,\sigma}\right] - \frac{1}{2}\,e^{-r\,T}\,\mathrm{Erfc}\left[\frac{\sqrt{T}\,\sigma - \frac{\sqrt{T}\,\left(r+\frac{\sigma^2}{2}\right)}{\sigma}}{\sqrt{2}}\right]$$

```
tabley1 = Table[FindRoot[y, {T, 2}],
  {r, {0.02, 0.03, 0.05, 0.07, 0.09, 0.10}}, {σ, {0.10, 0.20, 0.30, 0.40, 0.50}}]
```

```
{{{T → 11.5058}, {T → 6.24066}, {T → 3.52799}, {T → 2.19353}, {T → 1.47593}},
 {{T → 8.41311}, {T → 5.23194}, {T → 3.18184}, {T → 2.05438}, {T → 1.41151}},
 {{T → 5.43099}, {T → 3.94797}, {T → 2.6598}, {T → 1.82332}, {T → 1.29834}},
 {{T → 3.98885}, {T → 3.16408}, {T → 2.2842}, {T → 1.63904}, {T → 1.20206}},
 {{T → 3.14321}, {T → 2.63577}, {T → 2.0007}, {T → 1.4885}, {T → 1.11911}},
 {{T → 2.84003}, {T → 2.43142}, {T → 1.88347}, {T → 1.42309}, {T → 1.08179}}}
```

```
T4a1 = T /. tabley1
```

```
{{11.5058, 6.24066, 3.52799, 2.19353, 1.47593},
 {8.41311, 5.23194, 3.18184, 2.05438, 1.41151},
 {5.43099, 3.94797, 2.6598, 1.82332, 1.29834},
 {3.98885, 3.16408, 2.2842, 1.63904, 1.20206},
 {3.14321, 2.63577, 2.0007, 1.4885, 1.11911},
 {2.84003, 2.43142, 1.88347, 1.42309, 1.08179}}
```

```
T4TFa1 = TableForm[T4a1]
```

11.5058	6.24066	3.52799	2.19353	1.47593
8.41311	5.23194	3.18184	2.05438	1.41151
5.43099	3.94797	2.6598	1.82332	1.29834
3.98885	3.16408	2.2842	1.63904	1.20206
3.14321	2.63577	2.0007	1.4885	1.11911
2.84003	2.43142	1.88347	1.42309	1.08179

```
T4Fa1hd = TableForm[T4a1,
  TableHeadings → {{"0.02", "0.03", "0.05", "0.07", "0.09", "0.10"},
    {"0.10", "0.20", "0.30", "0.40", "0.50"}}]
```

	0.10	0.20	0.30	0.40	0.50
0.02	11.5058	6.24066	3.52799	2.19353	1.47593
0.03	8.41311	5.23194	3.18184	2.05438	1.41151
0.05	5.43099	3.94797	2.6598	1.82332	1.29834
0.07	3.98885	3.16408	2.2842	1.63904	1.20206
0.09	3.14321	2.63577	2.0007	1.4885	1.11911
0.10	2.84003	2.43142	1.88347	1.42309	1.08179

```
Export["Table4a1.xls", T4a1hd]
```

Table4a1.xls

```
a = 1.1
```

1.1

```
y = ct - 0.25
```

$$-0.25 + 0.55\,\mathrm{Erfc}\left[-\frac{0.0953102 + T\left(r + \frac{\sigma^2}{2}\right)}{\sqrt{2}\,\sqrt{T}\,\sigma}\right] - \frac{1}{2}\,e^{-r\,T}\,\mathrm{Erfc}\left[\frac{\sqrt{T}\,\sigma - \frac{0.0953102 + T\left(r + \frac{\sigma^2}{2}\right)}{\sqrt{T}\,\sigma}}{\sqrt{2}}\right]$$

```
tabley11 = Table[FindRoot[y, {T, 2}],
  {r, {0.02, 0.03, 0.05, 0.07, 0.09, 0.10}}, {σ, {0.10, 0.20, 0.30, 0.40, 0.50}}]
```

```
{{{T → 6.6228}, {T → 3.53666}, {T → 1.97469}, {T → 1.21967}, {T → 0.817662}},
 {{T → 4.83946}, {T → 2.98408}, {T → 1.79147}, {T → 1.14737}, {T → 0.784543}},
 {{T → 3.11069}, {T → 2.26623}, {T → 1.50993}, {T → 1.02556}, {T → 0.72571}},
 {{T → 2.27561}, {T → 1.82051}, {T → 1.30352}, {T → 0.926843}, {T → 0.675013}},
 {{T → 1.78789}, {T → 1.51731}, {T → 1.14564}, {T → 0.845167}, {T → 0.630856}},
 {{T → 1.6136}, {T → 1.39952}, {T → 1.07985}, {T → 0.809391}, {T → 0.610842}}}
```

```
T4a1 = T /. tabley11
```

```
{{6.6228, 3.53666, 1.97469, 1.21967, 0.817662},
 {4.83946, 2.98408, 1.79147, 1.14737, 0.784543},
 {3.11069, 2.26623, 1.50993, 1.02556, 0.72571},
 {2.27561, 1.82051, 1.30352, 0.926843, 0.675013},
 {1.78789, 1.51731, 1.14564, 0.845167, 0.630856},
 {1.6136, 1.39952, 1.07985, 0.809391, 0.610842}}
```

```
T4TFa1 = TableForm[T4a1]
```

6.6228	3.53666	1.97469	1.21967	0.817662
4.83946	2.98408	1.79147	1.14737	0.784543
3.11069	2.26623	1.50993	1.02556	0.72571
2.27561	1.82051	1.30352	0.926843	0.675013
1.78789	1.51731	1.14564	0.845167	0.630856
1.6136	1.39952	1.07985	0.809391	0.610842

```
T4Fa1hd = TableForm[T4a1,
  TableHeadings → {{"0.02", "0.03", "0.05", "0.07", "0.09", "0.10"},
    {"0.10", "0.20", "0.30", "0.40", "0.50"}}]
```

	0.10	0.20	0.30	0.40	0.50
0.02	6.6228	3.53666	1.97469	1.21967	0.817662
0.03	4.83946	2.98408	1.79147	1.14737	0.784543
0.05	3.11069	2.26623	1.50993	1.02556	0.72571
0.07	2.27561	1.82051	1.30352	0.926843	0.675013
0.09	1.78789	1.51731	1.14564	0.845167	0.630856
0.10	1.6136	1.39952	1.07985	0.809391	0.610842

```
Export["Table4a11.xls", T4Fa1hd]
```

```
Table4a11.xls
```

```
a = 1.2
```

```
1.2
```

```
y = ct - 0.25
```

$$-0.25 + 0.6\,\mathrm{Erfc}\left[-\frac{0.182322 + T\left(r + \frac{\sigma^2}{2}\right)}{\sqrt{2}\,\sqrt{T}\,\sigma}\right] - \frac{1}{2}\,e^{-r\,T}\,\mathrm{Erfc}\left[\frac{\sqrt{T}\,\sigma - \frac{0.182322 + T\left(r + \frac{\sigma^2}{2}\right)}{\sqrt{T}\,\sigma}}{\sqrt{2}}\right]$$

```
tabley12 = Table[FindRoot[y, {T, 0.5}],
  {r, {0.02, 0.03, 0.05, 0.07, 0.09, 0.10}}, {σ, {0.10, 0.20, 0.30, 0.40, 0.50}}]
```

```
{{{T → 2.31593}, {T → 1.30691}, {T → 0.733576}, {T → 0.453105}, {T → 0.303608}},
 {{T → 1.64255}, {T → 1.09378}, {T → 0.664585}, {T → 0.426304}, {T → 0.291445}},
 {{T → 1.01785}, {T → 0.81488}, {T → 0.557058}, {T → 0.380589}, {T → 0.269625}},
 {{T → 0.731445}, {T → 0.642279}, {T → 0.477309}, {T → 0.343065}, {T → 0.250616}},
 {{T → 0.569669}, {T → 0.526199}, {T → 0.41599}, {T → 0.311742}, {T → 0.233913}},
 {{T → 0.512815}, {T → 0.481605}, {T → 0.390405}, {T → 0.297953}, {T → 0.226301}}}
```

```
T4a12 = T /. tabley12

{{2.31593, 1.30691, 0.733576, 0.453105, 0.303608},
 {1.64255, 1.09378, 0.664585, 0.426304, 0.291445},
 {1.01785, 0.81488, 0.557058, 0.380589, 0.269625},
 {0.731445, 0.642279, 0.477309, 0.343065, 0.250616},
 {0.569669, 0.526199, 0.41599, 0.311742, 0.233913},
 {0.512815, 0.481605, 0.390405, 0.297953, 0.226301}}

T4TFa12 = TableForm[T4a12]
```

2.31593	1.30691	0.733576	0.453105	0.303608
1.64255	1.09378	0.664585	0.426304	0.291445
1.01785	0.81488	0.557058	0.380589	0.269625
0.731445	0.642279	0.477309	0.343065	0.250616
0.569669	0.526199	0.41599	0.311742	0.233913
0.512815	0.481605	0.390405	0.297953	0.226301

```
T4Fa12hd = TableForm[T4a012,
  TableHeadings → {{"0.02", "0.03", "0.05", "0.07", ".09", "0.10"},
    {"0.10", "0.20", "0.30", "0.40", "0.50"}}]

T4a012

Export["Table4a12.xls", T4Fa12hd]

Table4a12.xls
```
