# Supplementary material for: Corporate Social Responsibility: A Real Options Approach to the Challenge of Financial Sustainability
Source: PLoS One. 2015 May 4;10(5):e0125972. doi: 10.1371/journal.pone.0125972 (PMC4418608; doi:10.1371/journal.pone.0125972)
Supplement: S5 Table — (PDF) [file pone.0125972.s014.pdf]

S5: *Mathematica* code for Table 5

```
Clear[A, K, a, d1, d2, ct, σ, v, y, T, r, ϖ, pik, ct, tabley]
```

```
ndist = NormalDistribution[0, 1]
```

NormalDistribution[0, 1]

```
$Assumptions = σ > 0
```

$\sigma > 0$

$$d1 = \frac{\text{Log}[a] + \left(r + \frac{\sigma^2}{2}\right) * T}{\sigma * \sqrt{T}}$$

$$\frac{T\left(r + \frac{\sigma^2}{2}\right) + \text{Log}[a]}{\sqrt{T}\ \sigma}$$

$$d2 = d1 - \sigma * \sqrt{T}$$

$$-\sqrt{T}\ \sigma + \frac{T\left(r + \frac{\sigma^2}{2}\right) + \text{Log}[a]}{\sqrt{T}\ \sigma}$$

```
ct = a * CDF[ndist, d1] - Exp[-r * T] CDF[ndist, d2]
```

$$\frac{1}{2}\, a\, \text{Erfc}\left[-\frac{T\left(r + \frac{\sigma^2}{2}\right) + \text{Log}[a]}{\sqrt{2}\ \sqrt{T}\ \sigma}\right] - \frac{1}{2}\, e^{-r\,T}\, \text{Erfc}\left[\frac{\sqrt{T}\ \sigma - \frac{T\left(r+\frac{\sigma^2}{2}\right)+\text{Log}[a]}{\sqrt{T}\ \sigma}}{\sqrt{2}}\right]$$

```
Simplify[%]
```

$$\frac{1}{2}\left(-e^{-r\,T}\, \text{Erfc}\left[\frac{T\left(-2\, r + \sigma^2\right) - 2\, \text{Log}[a]}{2\sqrt{2}\ \sqrt{T}\ \sigma}\right] + a\, \text{Erfc}\left[-\frac{T\left(r + \frac{\sigma^2}{2}\right) + \text{Log}[a]}{\sqrt{2}\ \sqrt{T}\ \sigma}\right]\right)$$

```
r = 0.02
```

0.02

```
Tbvol010 = Table[FindRoot[ct == 0.1, {σ, 0.6}],
  {a, {0.30, 0.50, 0.60, 0.75, 0.90}}, {T, 2, 7, 1}]
```

```
{{{σ → 1.14421}, {σ → 0.928346}, {σ → 0.798833}, {σ → 0.709875},
  {σ → 0.643781}, {σ → 0.592072}}, {{σ → 0.707019}, {σ → 0.570245},
  {σ → 0.487699}, {σ → 0.430661}, {σ → 0.388022}, {σ → 0.354455}},
 {{σ → 0.565619}, {σ → 0.454164}, {σ → 0.386603}, {σ → 0.339709},
  {σ → 0.304489}, {σ → 0.27663}}, {{σ → 0.394033}, {σ → 0.312817},
  {σ → 0.26304}, {σ → 0.228089}, {σ → 0.201515}, {σ → 0.180219}},
 {{σ → 0.245361}, {σ → 0.189025}, {σ → 0.153466},
  {σ → 0.127636}, {σ → 0.107187}, {σ → 0.0899517}}}
```

```
Tablevol010 = σ /. Tbvol010
```

```
{{1.14421, 0.928346, 0.798833, 0.709875, 0.643781, 0.592072},
 {0.707019, 0.570245, 0.487699, 0.430661, 0.388022, 0.354455},
 {0.565619, 0.454164, 0.386603, 0.339709, 0.304489, 0.27663},
 {0.394033, 0.312817, 0.26304, 0.228089, 0.201515, 0.180219},
 {0.245361, 0.189025, 0.153466, 0.127636, 0.107187, 0.0899517}}
```

```
TableForm[Tablebvol010]
```

```
Tablebvol010
```

```
TableForm[Tablevol010, TableHeadings →
  {{"0.3", "0.5", "0.6", "0.75", "0.9"}, {"2", "3", "4", "5", "6", "7"}}]
```

	2	3	4	5	6	7
0.3	1.14421	0.928346	0.798833	0.709875	0.643781	0.592072
0.5	0.707019	0.570245	0.487699	0.430661	0.388022	0.354455
0.6	0.565619	0.454164	0.386603	0.339709	0.304489	0.27663
0.75	0.394033	0.312817	0.26304	0.228089	0.201515	0.180219
0.9	0.245361	0.189025	0.153466	0.127636	0.107187	0.0899517

```
Tablevol010
```


```
{{1.14421, 0.928346, 0.798833, 0.709875, 0.643781, 0.592072},
 {0.707019, 0.570245, 0.487699, 0.430661, 0.388022, 0.354455},
 {0.565619, 0.454164, 0.386603, 0.339709, 0.304489, 0.27663},
 {0.394033, 0.312817, 0.26304, 0.228089, 0.201515, 0.180219},
 {0.245361, 0.189025, 0.153466, 0.127636, 0.107187, 0.0899517}}
```


```
Export["Tableminvol010.xls", Tablevol010]
```

```
Tableminvol010.xls
```

```
Tbvol020 = Table[FindRoot[ct == 0.2, {σ, 0.6}],
  {a, {0.30, 0.50, 0.60, 0.75, 0.90}}, {T, 2, 7, 1}]
```

```
{{{σ → 1.81981}, {σ → 1.48048}, {σ → 1.27743}, {σ → 1.13835},
  {σ → 1.0353}, {σ → 0.954908}}, {{σ → 1.06652}, {σ → 0.863899},
  {σ → 0.742125}, {σ → 0.658336}, {σ → 0.59597}, {σ → 0.547087}},
 {{σ → 0.863256}, {σ → 0.697221}, {σ → 0.597141}, {σ → 0.528071},
  {σ → 0.476499}, {σ → 0.435947}}, {{σ → 0.631471}, {σ → 0.506705},
  {σ → 0.431002}, {σ → 0.378395}, {σ → 0.338832}, {σ → 0.307486}},
 {{σ → 0.443805}, {σ → 0.351551}, {σ → 0.294829},
  {σ → 0.254843}, {σ → 0.224293}, {σ → 0.199659}}}
```

```
TableForm[Tbvol020]
```

σ → 1.81981	σ → 1.48048	σ → 1.27743	σ → 1.13835	σ → 1.0353	σ → 0.[illegible]
σ → 1.06652	σ → 0.863899	σ → 0.742125	σ → 0.658336	σ → 0.59597	σ → 0.[illegible]
σ → 0.863256	σ → 0.697221	σ → 0.597141	σ → 0.528071	σ → 0.476499	σ → 0.[illegible]
σ → 0.631471	σ → 0.506705	σ → 0.431002	σ → 0.378395	σ → 0.338832	σ → 0.[illegible]
σ → 0.443805	σ → 0.351551	σ → 0.294829	σ → 0.254843	σ → 0.224293	σ → 0.[illegible]

```
Tablevol020 = σ /. Tbvol020
```

```
{{1.81981, 1.48048, 1.27743, 1.13835, 1.0353, 0.954908},
 {1.06652, 0.863899, 0.742125, 0.658336, 0.59597, 0.547087},
 {0.863256, 0.697221, 0.597141, 0.528071, 0.476499, 0.435947},
 {0.631471, 0.506705, 0.431002, 0.378395, 0.338832, 0.307486},
 {0.443805, 0.351551, 0.294829, 0.254843, 0.224293, 0.199659}}
```

```
TableForm[Tablevol020, TableHeadings →
  {{"0.3", "0.5", "0.6", "0.75", "0.9"}, {"2", "3", "4", "5", "6", "7"}}]
```

	2	3	4	5	6	7
0.3	1.81981	1.48048	1.27743	1.13835	1.0353	0.954908
0.5	1.06652	0.863899	0.742125	0.658336	0.59597	0.547087
0.6	0.863256	0.697221	0.597141	0.528071	0.476499	0.435947
0.75	0.631471	0.506705	0.431002	0.378395	0.338832	0.307486
0.9	0.443805	0.351551	0.294829	0.254843	0.224293	0.199659

```
Export["Tableminvol020.xls", Tablevol020]
```

```
Tableminvol020.xls
```

```
Tb025 = Table[FindRoot[ct == 0.25, {σ, 0.6}],
  {a, {0.30, 0.50, 0.60, 0.75, 0.90}}, {T, 2, 7, 1}]
```

```
{{{σ → 2.34584}, {σ → 1.91051}, {σ → 1.65033}, {σ → 1.47231},
  {σ → 1.34056}, {σ → 1.23789}}, {{σ → 1.2608}, {σ → 1.02276},
  {σ → 0.879911}, {σ → 0.781769}, {σ → 0.70883}, {σ → 0.651749}},
 {{σ → 1.01843}, {σ → 0.82412}, {σ → 0.707222}, {σ → 0.626703},
  {σ → 0.566702}, {σ → 0.519619}}, {{σ → 0.753049}, {σ → 0.60619},
  {σ → 0.517362}, {σ → 0.455836}, {σ → 0.409722}, {σ → 0.373316}},
 {{σ → 0.545589}, {σ → 0.435045}, {σ → 0.367524},
  {σ → 0.320264}, {σ → 0.284439}, {σ → 0.255805}}}
```

```
TableForm[Tb025]
```

σ → 2.34584	σ → 1.91051	σ → 1.65033	σ → 1.47231	σ → 1.34056	σ → 1.[illegible]
σ → 1.2608	σ → 1.02276	σ → 0.879911	σ → 0.781769	σ → 0.70883	σ → 0.[illegible]
σ → 1.01843	σ → 0.82412	σ → 0.707222	σ → 0.626703	σ → 0.566702	σ → 0.[illegible]
σ → 0.753049	σ → 0.60619	σ → 0.517362	σ → 0.455836	σ → 0.409722	σ → 0.[illegible]
σ → 0.545589	σ → 0.435045	σ → 0.367524	σ → 0.320264	σ → 0.284439	σ → 0.[illegible]

```
Table025 = σ /. Tb025
```

```
{{2.34584, 1.91051, 1.65033, 1.47231, 1.34056, 1.23789},
 {1.2608, 1.02276, 0.879911, 0.781769, 0.70883, 0.651749},
 {1.01843, 0.82412, 0.707222, 0.626703, 0.566702, 0.519619},
 {0.753049, 0.60619, 0.517362, 0.455836, 0.409722, 0.373316},
 {0.545589, 0.435045, 0.367524, 0.320264, 0.284439, 0.255805}}
```

```
TableForm[Table025, TableHeadings →
  {{"0.30", "0.50", "0.60", "0.75", "0.90"}, {"2", "3", "4", "5", "6", "7"}}]
```

	2	3	4	5	6	7
0.30	2.34584	1.91051	1.65033	1.47231	1.34056	1.23789
0.50	1.2608	1.02276	0.879911	0.781769	0.70883	0.651749
0.60	1.01843	0.82412	0.707222	0.626703	0.566702	0.519619
0.75	0.753049	0.60619	0.517362	0.455836	0.409722	0.373316
0.90	0.545589	0.435045	0.367524	0.320264	0.284439	0.255805

```
Export["Tableminvol025.xls", Table025]
```

```
Tableminvol025.xls
```

```
Tbvol030 =
 Table[FindRoot[ct == 0.3, {σ, 0.6}], {a, {0.50, 0.60, 0.75, 0.90}}, {T, 2, 7, 1}]
```

```
{{{σ → 1.47717}, {σ → 1.19974}, {σ → 1.03345}, {σ → 0.919349},
  {σ → 0.834655}, {σ → 0.768462}}, {{σ → 1.18397}, {σ → 0.959555},
  {σ → 0.824753}, {σ → 0.732048}, {σ → 0.663079}, {σ → 0.609048}},
 {{σ → 0.879288}, {σ → 0.70954}, {σ → 0.607121}, {σ → 0.536361},
  {σ → 0.483465}, {σ → 0.44182}}, {{σ → 0.650135}, {σ → 0.520817},
  {σ → 0.442199}, {σ → 0.387446}, {σ → 0.346165}, {σ → 0.313365}}}
```

```
TableForm[Tbvol030]
```

```
σ → 1.47717    σ → 1.19974    σ → 1.03345    σ → 0.919349   σ → 0.834655   σ → 0.7
σ → 1.18397    σ → 0.959555   σ → 0.824753   σ → 0.732048   σ → 0.663079   σ → 0.6
σ → 0.879288   σ → 0.70954    σ → 0.607121   σ → 0.536361   σ → 0.483465   σ → 0.4
σ → 0.650135   σ → 0.520817   σ → 0.442199   σ → 0.387446   σ → 0.346165   σ → 0.3
```

```
Tablevol030 = σ /. Tbvol030
```

```
{{1.47717, 1.19974, 1.03345, 0.919349, 0.834655, 0.768462},
 {1.18397, 0.959555, 0.824753, 0.732048, 0.663079, 0.609048},
 {0.879288, 0.70954, 0.607121, 0.536361, 0.483465, 0.44182},
 {0.650135, 0.520817, 0.442199, 0.387446, 0.346165, 0.313365}}
```

```
TableForm[Tablevol030,
 TableHeadings → {{"0.5", "0.6", "0.75", "0.9"}, {"2", "3", "4", "5", "6", "7"}}]
```

	2	3	4	5	6	7
0.5	1.47717	1.19974	1.03345	0.919349	0.834655	0.768462
0.6	1.18397	0.959555	0.824753	0.732048	0.663079	0.609048
0.75	0.879288	0.70954	0.607121	0.536361	0.483465	0.44182
0.9	0.650135	0.520817	0.442199	0.387446	0.346165	0.313365

```
Export["Tableminvol030.xls", Tablevol030]
```

```
Tableminvol030.xls
```

```
Tbvol050 = Table[FindRoot[ct == 0.5, {σ, 0.6}], {a, {0.60, 0.75, 0.90}}, {T, 2, 7, 1}]
```

```
{{{σ → 2.12591}, {σ → 1.73023},
  {σ → 1.49357}, {σ → 1.33153}, {σ → 1.21152}, {σ → 1.11793}},
 {{σ → 1.47908}, {σ → 1.20067}, {σ → 1.03371}, {σ → 0.919086}, {σ → 0.833951},
  {σ → 0.767371}}, {{σ → 1.11531}, {σ → 0.902298}, {σ → 0.774102},
  {σ → 0.685763}, {σ → 0.619904}, {σ → 0.568194}}}
```

```
TableForm[Tbvol050]
```

```
σ → 2.12591   σ → 1.73023    σ → 1.49357    σ → 1.33153    σ → 1.21152    σ → 1.1
σ → 1.47908   σ → 1.20067    σ → 1.03371    σ → 0.919086   σ → 0.833951   σ → 0.76
σ → 1.11531   σ → 0.902298   σ → 0.774102   σ → 0.685763   σ → 0.619904   σ → 0.56
```

```
Tablevol050 = σ /. Tbvol050
```

```
{{2.12591, 1.73023, 1.49357, 1.33153, 1.21152, 1.11793},
 {1.47908, 1.20067, 1.03371, 0.919086, 0.833951, 0.767371},
 {1.11531, 0.902298, 0.774102, 0.685763, 0.619904, 0.568194}}
```

```
TableForm[Tablevol050,
 TableHeadings → {{"0.6", "0.75", "0.9"}, {"2", "3", "4", "5", "6", "7"}}]
```

	2	3	4	5	6	7
0.6	2.12591	1.73023	1.49357	1.33153	1.21152	1.11793
0.75	1.47908	1.20067	1.03371	0.919086	0.833951	0.767371
0.9	1.11531	0.902298	0.774102	0.685763	0.619904	0.568194

```
Export["Tableminvol050.xls", Tablevol050]
```

Tableminvol050.xls

```
Tbvol075 = Table[FindRoot[ct == 0.75, {σ, 0.6}], {a, {0.8, 0.9}}, {T, 2, 7, 1}]
```

```
{{{σ → 2.69083}, {σ → 2.19209}, {σ → 1.89409}, {σ → 1.69025},
  {σ → 1.53943}, {σ → 1.42194}}, {{σ → 1.98074}, {σ → 1.61109},
  {σ → 1.38986}, {σ → 1.23829}, {σ → 1.12595}, {σ → 1.03829}}}
```

```
TableForm[Tbvol075]
```

σ → 2.69083	σ → 2.19209	σ → 1.89409	σ → 1.69025	σ → 1.53943	σ → 1.42194
σ → 1.98074	σ → 1.61109	σ → 1.38986	σ → 1.23829	σ → 1.12595	σ → 1.03829

```
Tablevol075 = σ /. Tbvol075
```

```
{{2.69083, 2.19209, 1.89409, 1.69025, 1.53943, 1.42194},
 {1.98074, 1.61109, 1.38986, 1.23829, 1.12595, 1.03829}}
```

```
TableForm[Tablevol075,
 TableHeadings → {{"0.8", "0.9"}, {"2", "3", "4", "5", "6", "7"}}]
```

	2	3	4	5	6	7
0.8	2.69083	2.19209	1.89409	1.69025	1.53943	1.42194
0.9	1.98074	1.61109	1.38986	1.23829	1.12595	1.03829

```
Export["Tableminvol075.xls", Tablevol075]
```

Tableminvol075.xls
