# Supplementary material for: Corporate Social Responsibility: A Real Options Approach to the Challenge of Financial Sustainability
Source: PLoS One. 2015 May 4;10(5):e0125972. doi: 10.1371/journal.pone.0125972 (PMC4418608; doi:10.1371/journal.pone.0125972)
Supplement: S6 Table — (PDF) [file pone.0125972.s015.pdf]

S6 Table: *Mathematica* code for Table 6

```
Clear[ndist, υ, T, sbs, dx1, dx2, θ, osr, csv, SPV, CPV]
```

SPV = Savings Present Value

CPV = Cost Present Value

sbs = Substitution option

osr = opportunity savings ratio

csv = cost/savings ratio

```
ndist = NormalDistribution[0, 1]
NormalDistribution[0, 1]
```

$$\theta = \sqrt{\sigma SPV^2 + \sigma CPV^2 - 2 * \rho * \sigma SPV * \sigma CPV}$$

$$\sqrt{\sigma CPV^2 - 2\ \rho\ \sigma CPV\ \sigma SPV + \sigma SPV^2}$$

Let us apply Margrabe's formula:

$$dx1 = \frac{\mathrm{Log}\left[\frac{1}{csv}\right] + \left(\frac{\upsilon^2}{2}\right) * T}{\upsilon * \sqrt{T}}$$

$$\frac{\frac{T\,\upsilon^2}{2} + \mathrm{Log}\left[\frac{1}{csv}\right]}{\sqrt{T}\ \upsilon}$$

$$dx2 = dx1 - \upsilon * \sqrt{T}$$

$$-\sqrt{T}\ \upsilon + \frac{\frac{T\,\upsilon^2}{2} + \mathrm{Log}\left[\frac{1}{csv}\right]}{\sqrt{T}\ \upsilon}$$

```
osr = CDF[ndist, dx1] - csv * CDF[ndist, dx2]
```

$$\frac{1}{2}\ \mathrm{Erfc}\left[-\frac{\frac{T\,\upsilon^2}{2} + \mathrm{Log}\left[\frac{1}{csv}\right]}{\sqrt{2}\ \sqrt{T}\ \upsilon}\right] - \frac{1}{2}\ csv\ \mathrm{Erfc}\left[\frac{\sqrt{T}\ \upsilon - \frac{\frac{T\,\upsilon^2}{2}+\mathrm{Log}\left[\frac{1}{csv}\right]}{\sqrt{T}\ \upsilon}}{\sqrt{2}}\right]$$

```
υ = 0.1
0.1
```

```
Clear[tableOsR010]
```

```
TableOSR010 =
 Table[osr, {T, {3, 5, 10, 15, 20, 25, 50}}, {csv, {0.25, 0.5, 0.75, 1, 1.25}}]
```

```
{{0.75, 0.500001, 0.253007, 0.0690126, 0.00901434},
 {0.75, 0.500042, 0.259033, 0.0890207, 0.0208285},
 {0.75, 0.501107, 0.27681, 0.125633, 0.0496817},
 {0.750008, 0.503955, 0.294067, 0.153549, 0.0751581},
 {0.750059, 0.508113, 0.31002, 0.176937, 0.0978062},
 {0.750203, 0.513069, 0.324757, 0.197413, 0.118287},
 {0.753176, 0.54158, 0.385554, 0.276326, 0.200514}}
```

```
TableForm[TableOSR010]
```

0.75	0.500001	0.253007	0.0690126	0.00901434
0.75	0.500042	0.259033	0.0890207	0.0208285
0.75	0.501107	0.27681	0.125633	0.0496817
0.750008	0.503955	0.294067	0.153549	0.0751581
0.750059	0.508113	0.31002	0.176937	0.0978062
0.750203	0.513069	0.324757	0.197413	0.118287
0.753176	0.54158	0.385554	0.276326	0.200514

```
Export["OSR010.xls", TableOSR010]
```

OSR010.xls

```
TableOSR020 =
 Table[osr, {T, {3, 5, 10, 15, 20, 25, 50}}, {csv, {0.25, 0.5, 0.75, 1, 1.25}}]
```

```
{{0.75, 0.500001, 0.253007, 0.0690126, 0.00901434},
 {0.75, 0.500042, 0.259033, 0.0890207, 0.0208285},
 {0.75, 0.501107, 0.27681, 0.125633, 0.0496817},
 {0.750008, 0.503955, 0.294067, 0.153549, 0.0751581},
 {0.750059, 0.508113, 0.31002, 0.176937, 0.0978062},
 {0.750203, 0.513069, 0.324757, 0.197413, 0.118287},
 {0.753176, 0.54158, 0.385554, 0.276326, 0.200514}}
```

```
TableForm[TableOSR020]
```

0.75	0.500001	0.253007	0.0690126	0.00901434
0.75	0.500042	0.259033	0.0890207	0.0208285
0.75	0.501107	0.27681	0.125633	0.0496817
0.750008	0.503955	0.294067	0.153549	0.0751581
0.750059	0.508113	0.31002	0.176937	0.0978062
0.750203	0.513069	0.324757	0.197413	0.118287
0.753176	0.54158	0.385554	0.276326	0.200514

```
Export["OSR020.xls", TableOSR020]
```

OSR020.xls

```
υ = 0.30
```

0.3

```
TableOSR030 =
 Table[osr, {T, {3, 5, 10, 15, 20, 25, 50}}, {csv, {0.25, 0.5, 0.75, 1, 1.25}}]
```

```
{{0.750297, 0.515195, 0.330352, 0.204988, 0.125982},
 {0.752275, 0.535767, 0.374727, 0.262684, 0.186042},
 {0.764002, 0.585325, 0.457538, 0.364744, 0.295759},
 {0.779527, 0.627389, 0.519243, 0.438724, 0.376622},
 {0.795445, 0.66311, 0.568948, 0.497665, 0.441474},
 {0.810668, 0.693907, 0.610587, 0.546745, 0.495672},
 {0.871264, 0.801591, 0.751102, 0.711156, 0.678033}}
```

```
TableForm[TableOSR030]
```

0.750297	0.515195	0.330352	0.204988	0.125982
0.752275	0.535767	0.374727	0.262684	0.186042
0.764002	0.585325	0.457538	0.364744	0.295759
0.779527	0.627389	0.519243	0.438724	0.376622
0.795445	0.66311	0.568948	0.497665	0.441474
0.810668	0.693907	0.610587	0.546745	0.495672
0.871264	0.801591	0.751102	0.711156	0.678033

```
Export["OSR030.xls", TableOSR030]
```

```
OSR030.xls
```

```
υ = 0.50
```

```
0.5
```

```
TableOSR050 =
 Table[osr, {T, {3, 5, 10, 15, 20, 25, 50}}, {csv, {0.25, 0.5, 0.75, 1, 1.25}}]
```

```
{{0.759376, 0.569622, 0.43304, 0.334994, 0.263492},
 {0.775977, 0.618647, 0.506765, 0.42385, 0.360307},
 {0.81869, 0.709281, 0.631063, 0.570805, 0.52229},
 {0.853827, 0.772196, 0.713313, 0.667078, 0.629051},
 {0.88159, 0.818591, 0.772815, 0.736448, 0.706162},
 {0.903581, 0.853981, 0.817733, 0.7887, 0.764322},
 {0.963493, 0.94622, 0.933386, 0.9229, 0.913921}}
```

```
TableForm[TableOSR050]
```

0.759376	0.569622	0.43304	0.334994	0.263492
0.775977	0.618647	0.506765	0.42385	0.360307
0.81869	0.709281	0.631063	0.570805	0.52229
0.853827	0.772196	0.713313	0.667078	0.629051
0.88159	0.818591	0.772815	0.736448	0.706162
0.903581	0.853981	0.817733	0.7887	0.764322
0.963493	0.94622	0.933386	0.9229	0.913921

```
Export["OSR050.xls", TableOSR050]
```

```
OSR050.xls
```
