# Supplementary material for: Corporate Social Responsibility: A Real Options Approach to the Challenge of Financial Sustainability
Source: PLoS One. 2015 May 4;10(5):e0125972. doi: 10.1371/journal.pone.0125972 (PMC4418608; doi:10.1371/journal.pone.0125972)
Supplement: S7 Table — (PDF) [file pone.0125972.s016.pdf]

S7Table: *Mathematica* code for Table 7

```
Clear[ndist, υ, T, sbs, dx1, dx2, θ, osr, csv, SPV, CPV]
```

SPV = Savings Present Value

CPV = Cost Present Value

sbs = Substitution option

osr = opportunity savings ratio

csv = cost/savings ratio

```
ndist = NormalDistribution[0, 1]
```

```
NormalDistribution[0, 1]
```

$$\theta = \sqrt{\sigma SPV^2 + \sigma CPV^2 - 2 * \rho * \sigma SPV * \sigma CPV}$$

$$\sqrt{\sigma CPV^2 - 2\,\rho\,\sigma CPV\,\sigma SPV + \sigma SPV^2}$$

Let us apply Margrabe's formula:

$$dx1 = \frac{Log\left[\frac{1}{csv}\right] + \left(\frac{\upsilon^2}{2}\right) * T}{\upsilon * \sqrt{T}}$$

$$\frac{\frac{T\,\upsilon^2}{2} + Log\left[\frac{1}{csv}\right]}{\sqrt{T}\,\upsilon}$$

$$dx2 = dx1 - \upsilon * \sqrt{T}$$

$$-\sqrt{T}\,\upsilon + \frac{\frac{T\,\upsilon^2}{2} + Log\left[\frac{1}{csv}\right]}{\sqrt{T}\,\upsilon}$$

```
osr = CDF[ndist, dx1] - csv * CDF[ndist, dx2]
```

$$\frac{1}{2}\,Erfc\left[-\frac{\frac{T\,\upsilon^2}{2} + Log\left[\frac{1}{csv}\right]}{\sqrt{2}\,\sqrt{T}\,\upsilon}\right] - \frac{1}{2}\,csv\,Erfc\left[\frac{\sqrt{T}\,\upsilon - \frac{\frac{T\,\upsilon^2}{2}+Log\left[\frac{1}{csv}\right]}{\sqrt{T}\,\upsilon}}{\sqrt{2}}\right]$$

```
Clear[m]
```

```
m = osr - 0.10
```

$$-0.1 + \frac{1}{2}\,Erfc\left[-\frac{\frac{T\,\upsilon^2}{2} + Log\left[\frac{1}{csv}\right]}{\sqrt{2}\,\sqrt{T}\,\upsilon}\right] - \frac{1}{2}\,csv\,Erfc\left[\frac{\sqrt{T}\,\upsilon - \frac{\frac{T\,\upsilon^2}{2}+Log\left[\frac{1}{csv}\right]}{\sqrt{T}\,\upsilon}}{\sqrt{2}}\right]$$

```
m1 = Table[FindRoot[m, {csv, 1}],
  {T, {1, 2, 3, 4, 5}}, {υ, {0.10, 0.20, 0.30, 0.40, 0.5}}]
```

```
{{{csv → 0.908579}, {csv → 0.959405}, {csv → 1.04688},
  {csv → 1.16953}, {csv → 1.33175}}, {{csv → 0.924681},
  {csv → 1.02943}, {csv → 1.205}, {csv → 1.46371}, {csv → 1.8342}},
 {{csv → 0.941914}, {csv → 1.09926}, {csv → 1.36887}, {csv → 1.79052},
  {csv → 2.44324}}, {{csv → 0.959405}, {csv → 1.16953},
  {csv → 1.5419}, {csv → 2.16005}, {csv → 3.191}}, {{csv → 0.976942},
  {csv → 1.24076}, {csv → 1.72625}, {csv → 2.58066}, {csv → 4.11153}}}
```

```
T71 = csv /. m1
```

```
{{0.908579, 0.959405, 1.04688, 1.16953, 1.33175},
 {0.924681, 1.02943, 1.205, 1.46371, 1.8342},
 {0.941914, 1.09926, 1.36887, 1.79052, 2.44324},
 {0.959405, 1.16953, 1.5419, 2.16005, 3.191},
 {0.976942, 1.24076, 1.72625, 2.58066, 4.11153}}
```

```
T7csv010 =
 TableForm[T71, TableHeadings → {{1, 2, 3, 4, 5}, {0.10, 0.20, 0.30, 0.40, 0.50}}]
```

	0.1	0.2	0.3	0.4	0.5
1	0.908579	0.959405	1.04688	1.16953	1.33175
2	0.924681	1.02943	1.205	1.46371	1.8342
3	0.941914	1.09926	1.36887	1.79052	2.44324
4	0.959405	1.16953	1.5419	2.16005	3.191
5	0.976942	1.24076	1.72625	2.58066	4.11153

```
Export["Table7csv010.xls", T7csv010]
```

```
Table7csv010.xls
```

```
Clear[m]
```

```
m = osr - 0.20
```

$$-0.2+\frac{1}{2}\,\mathrm{Erfc}\left[-\frac{\frac{T\upsilon^2}{2}+\mathrm{Log}\left[\frac{1}{csv}\right]}{\sqrt{2}\,\sqrt{T}\,\upsilon}\right]-\frac{1}{2}\,csv\,\mathrm{Erfc}\left[\frac{\sqrt{T}\,\upsilon-\frac{\frac{T\upsilon^2}{2}+\mathrm{Log}\left[\frac{1}{csv}\right]}{\sqrt{T}\,\upsilon}}{\sqrt{2}}\right]$$

```
m2 = Table[FindRoot[m, {csv, 1}],
  {T, {1, 2, 3, 4, 5}}, {υ, {0.10, 0.20, 0.30, 0.40, 0.5}}]
```

```
{{{csv → 0.800405}, {csv → 0.814215}, {csv → 0.851429},
  {csv → 0.911003}, {csv → 0.993592}}, {{csv → 0.803316},
  {csv → 0.843438}, {csv → 0.928855}, {csv → 1.06162}, {csv → 1.25205}},
 {{csv → 0.8082}, {csv → 0.876311}, {csv → 1.0127}, {csv → 1.22973},
  {csv → 1.55822}}, {{csv → 0.814215}, {csv → 0.911003},
  {csv → 1.10197}, {csv → 1.41703}, {csv → 1.92197}}, {{csv → 0.820931},
  {csv → 0.947004}, {csv → 1.19682}, {csv → 1.62602}, {csv → 2.35478}}}
```

T72 = csv /. m2

```
{{0.800405, 0.814215, 0.851429, 0.911003, 0.993592},
 {0.803316, 0.843438, 0.928855, 1.06162, 1.25205},
 {0.8082, 0.876311, 1.0127, 1.22973, 1.55822},
 {0.814215, 0.911003, 1.10197, 1.41703, 1.92197},
 {0.820931, 0.947004, 1.19682, 1.62602, 2.35478}}
```

T7csv020 =
TableForm[T72, TableHeadings → {{1, 2, 3, 4, 5}, {0.10, 0.20, 0.30, 0.40, 0.50}}]

	0.1	0.2	0.3	0.4	0.5
1	0.800405	0.814215	0.851429	0.911003	0.993592
2	0.803316	0.843438	0.928855	1.06162	1.25205
3	0.8082	0.876311	1.0127	1.22973	1.55822
4	0.814215	0.911003	1.10197	1.41703	1.92197
5	0.820931	0.947004	1.19682	1.62602	2.35478

Export["Table7csv020.xls", T7csv020]

Table7csv020.xls

Clear[m]

m = osr - 0.30

$$-0.3+\frac{1}{2}\,\mathrm{Erfc}\left[-\frac{\frac{T\upsilon^2}{2}+\mathrm{Log}\left[\frac{1}{csv}\right]}{\sqrt{2}\,\sqrt{T}\,\upsilon}\right]-\frac{1}{2}\,csv\,\mathrm{Erfc}\left[\frac{\sqrt{T}\,\upsilon-\frac{\frac{T\upsilon^2}{2}+\mathrm{Log}\left[\frac{1}{csv}\right]}{\sqrt{T}\,\upsilon}}{\sqrt{2}}\right]$$

m3 = Table[FindRoot[m, {csv, 1}],
{T, {1, 2, 3, 4, 5}}, {υ, {0.10, 0.20, 0.30, 0.40, 0.5}}]

```
{{{csv → 0.700004}, {csv → 0.702604}, {csv → 0.717},
  {csv → 0.746234}, {csv → 0.790778}}, {{csv → 0.700223},
  {csv → 0.713491}, {csv → 0.75561}, {csv → 0.828846}, {csv → 0.937523}},
 {{csv → 0.701071}, {csv → 0.728696}, {csv → 0.801387}, {csv → 0.924725},
  {csv → 1.11284}}, {{csv → 0.702604}, {csv → 0.746234},
  {csv → 0.851707}, {csv → 1.03215}, {csv → 1.31866}}, {{csv → 0.704713},
  {csv → 0.765313}, {csv → 0.905862}, {csv → 1.15145}, {csv → 1.55932}}}
```

T73 = csv /. m3

```
{{0.700004, 0.702604, 0.717, 0.746234, 0.790778},
 {0.700223, 0.713491, 0.75561, 0.828846, 0.937523},
 {0.701071, 0.728696, 0.801387, 0.924725, 1.11284},
 {0.702604, 0.746234, 0.851707, 1.03215, 1.31866},
 {0.704713, 0.765313, 0.905862, 1.15145, 1.55932}}
```

```
T7csv030 =
  TableForm[T73, TableHeadings → {{1, 2, 3, 4, 5}, {0.10, 0.20, 0.30, 0.40, 0.50}}]
```

	0.1	0.2	0.3	0.4	0.5
1	0.700004	0.702604	0.717	0.746234	0.790778
2	0.700223	0.713491	0.75561	0.828846	0.937523
3	0.701071	0.728696	0.801387	0.924725	1.11284
4	0.702604	0.746234	0.851707	1.03215	1.31866
5	0.704713	0.765313	0.905862	1.15145	1.55932

```
Export["Table7csv030.xls", T7csv030]
```

Table7csv030.xls

```
Clear[m]
```

```
m = osr - 0.40
```

$$-0.4+\frac{1}{2}\operatorname{Erfc}\left[-\frac{\frac{T\,\upsilon^2}{2}+\operatorname{Log}\left[\frac{1}{csv}\right]}{\sqrt{2}\,\sqrt{T}\,\upsilon}\right]-\frac{1}{2}\,csv\,\operatorname{Erfc}\left[\frac{\sqrt{T}\,\upsilon-\frac{\frac{T\,\upsilon^2}{2}+\operatorname{Log}\left[\frac{1}{csv}\right]}{\sqrt{T}\,\upsilon}}{\sqrt{2}}\right]$$

```
m4 = Table[FindRoot[m, {csv, 1}],
   {T, {1, 2, 3, 4, 5}}, {υ, {0.10, 0.20, 0.30, 0.40, 0.5}}]
```

```
{{{csv → 0.6}, {csv → 0.600263}, {csv → 0.604461},
   {csv → 0.61716}, {csv → 0.639936}}, {{csv → 0.600004},
   {csv → 0.603214}, {csv → 0.62173}, {csv → 0.660749}, {csv → 0.722824}},
  {{csv → 0.600061}, {csv → 0.60916}, {csv → 0.645651}, {csv → 0.715403},
   {csv → 0.8255}}, {{csv → 0.600263}, {csv → 0.61716}, {csv → 0.673569},
   {csv → 0.778099}, {csv → 0.946346}}, {{csv → 0.60067},
   {csv → 0.62661}, {csv → 0.704503}, {csv → 0.848197}, {csv → 1.0866}}}
```

```
T74 = csv /. m4
```

```
{{0.6, 0.600263, 0.604461, 0.61716, 0.639936},
 {0.600004, 0.603214, 0.62173, 0.660749, 0.722824},
 {0.600061, 0.60916, 0.645651, 0.715403, 0.8255},
 {0.600263, 0.61716, 0.673569, 0.778099, 0.946346},
 {0.60067, 0.62661, 0.704503, 0.848197, 1.0866}}
```

```
T7csv040 =
  TableForm[T74, TableHeadings → {{1, 2, 3, 4, 5}, {0.10, 0.20, 0.30, 0.40, 0.50}}]
```

	0.1	0.2	0.3	0.4	0.5
1	0.6	0.600263	0.604461	0.61716	0.639936
2	0.600004	0.603214	0.62173	0.660749	0.722824
3	0.600061	0.60916	0.645651	0.715403	0.8255
4	0.600263	0.61716	0.673569	0.778099	0.946346
5	0.60067	0.62661	0.704503	0.848197	1.0866

```
Export["Table7csv040.xls", T7csv040]
```

Table7csv040.xls

```
Clear[m]
```

m = osr - 0.50

$$-0.5+\frac{1}{2}\,\mathrm{Erfc}\left[-\frac{\frac{T\,\upsilon^2}{2}+\mathrm{Log}\left[\frac{1}{\mathrm{csv}}\right]}{\sqrt{2}\,\sqrt{T}\,\upsilon}\right]-\frac{1}{2}\,\mathrm{csv}\,\mathrm{Erfc}\left[\frac{\sqrt{T}\,\upsilon-\frac{\frac{T\,\upsilon^2}{2}+\mathrm{Log}\left[\frac{1}{\mathrm{csv}}\right]}{\sqrt{T}\,\upsilon}}{\sqrt{2}}\right]$$

m5 = Table[FindRoot[m, {csv, 1}],
{T, {1, 2, 3, 4, 5}}, {υ, {0.10, 0.20, 0.30, 0.40, 0.5}}]

```
{{{csv → 0.5}, {csv → 0.500009}, {csv → 0.500758},
  {csv → 0.505023}, {csv → 0.515097}}, {{csv → 0.5}, {csv → 0.500468},
  {csv → 0.50688}, {csv → 0.525424}, {csv → 0.558771}}, {{csv → 0.500001},
  {csv → 0.502123}, {csv → 0.517859}, {csv → 0.554667}, {csv → 0.616954}},
 {{csv → 0.500009}, {csv → 0.505023}, {csv → 0.532075}, {csv → 0.589855},
  {csv → 0.686741}}, {{csv → 0.500042}, {csv → 0.508972},
  {csv → 0.548683}, {csv → 0.63001}, {csv → 0.767925}}}
```

T75 = csv /. m5

```
{{0.5, 0.500009, 0.500758, 0.505023, 0.515097},
 {0.5, 0.500468, 0.50688, 0.525424, 0.558771},
 {0.500001, 0.502123, 0.517859, 0.554667, 0.616954},
 {0.500009, 0.505023, 0.532075, 0.589855, 0.686741},
 {0.500042, 0.508972, 0.548683, 0.63001, 0.767925}}
```

T7csv050 =
TableForm[T75, TableHeadings → {{1, 2, 3, 4, 5}, {0.10, 0.20, 0.30, 0.40, 0.50}}]

	0.1	0.2	0.3	0.4	0.5
1	0.5	0.500009	0.500758	0.505023	0.515097
2	0.5	0.500468	0.50688	0.525424	0.558771
3	0.500001	0.502123	0.517859	0.554667	0.616954
4	0.500009	0.505023	0.532075	0.589855	0.686741
5	0.500042	0.508972	0.548683	0.63001	0.767925

Export["Table7csv050.xls", T7csv050]

Table7csv050.xls
